# Performance of missing transverse momentum reconstruction with the ATLAS detector using proton–proton collisions at $\sqrt{s}=13~\hbox {TeV}$

**DOI:** 10.1140/epjc/s10052-018-6288-9

**Published:** 2018-11-08

**Authors:** M. Aaboud, G. Aad, B. Abbott, O. Abdinov, B. Abeloos, S. H. Abidi, O. S. AbouZeid, N. L. Abraham, H. Abramowicz, H. Abreu, R. Abreu, Y. Abulaiti, B. S. Acharya, S. Adachi, L. Adamczyk, J. Adelman, M. Adersberger, T. Adye, A. A. Affolder, Y. Afik, T. Agatonovic-Jovin, C. Agheorghiesei, J. A. Aguilar-Saavedra, F. Ahmadov, G. Aielli, S. Akatsuka, H. Akerstedt, T. P. A. Åkesson, E. Akilli, A. V. Akimov, G. L. Alberghi, J. Albert, P. Albicocco, M. J. Alconada Verzini, S. Alderweireldt, M. Aleksa, I. N. Aleksandrov, C. Alexa, G. Alexander, T. Alexopoulos, M. Alhroob, B. Ali, G. Alimonti, J. Alison, S. P. Alkire, B. M. M. Allbrooke, B. W. Allen, P. P. Allport, A. Aloisio, A. Alonso, F. Alonso, C. Alpigiani, A. A. Alshehri, M. I. Alstaty, B. Alvarez Gonzalez, D. Álvarez Piqueras, M. G. Alviggi, B. T. Amadio, Y. Amaral Coutinho, C. Amelung, D. Amidei, S. P. Amor Dos Santos, S. Amoroso, G. Amundsen, C. Anastopoulos, L. S. Ancu, N. Andari, T. Andeen, C. F. Anders, J. K. Anders, K. J. Anderson, A. Andreazza, V. Andrei, S. Angelidakis, I. Angelozzi, A. Angerami, A. V. Anisenkov, N. Anjos, A. Annovi, C. Antel, M. Antonelli, A. Antonov, D. J. A. Antrim, F. Anulli, M. Aoki, L. Aperio Bella, G. Arabidze, Y. Arai, J. P. Araque, V. Araujo Ferraz, A. T. H. Arce, R. E. Ardell, F. A. Arduh, J-F. Arguin, S. Argyropoulos, M. Arik, A. J. Armbruster, L. J. Armitage, O. Arnaez, H. Arnold, M. Arratia, O. Arslan, A. Artamonov, G. Artoni, S. Artz, S. Asai, N. Asbah, A. Ashkenazi, L. Asquith, K. Assamagan, R. Astalos, M. Atkinson, N. B. Atlay, K. Augsten, G. Avolio, B. Axen, M. K. Ayoub, G. Azuelos, A. E. Baas, M. J. Baca, H. Bachacou, K. Bachas, M. Backes, P. Bagnaia, M. Bahmani, H. Bahrasemani, J. T. Baines, M. Bajic, O. K. Baker, E. M. Baldin, P. Balek, F. Balli, W. K. Balunas, E. Banas, A. Bandyopadhyay, S. Banerjee, A. A. E. Bannoura, L. Barak, E. L. Barberio, D. Barberis, M. Barbero, T. Barillari, M-S. Barisits, J. Barkeloo, T. Barklow, N. Barlow, S. L. Barnes, B. M. Barnett, R. M. Barnett, Z. Barnovska-Blenessy, A. Baroncelli, G. Barone, A. J. Barr, L. Barranco Navarro, F. Barreiro, J. Barreiro Guimarães da Costa, R. Bartoldus, A. E. Barton, P. Bartos, A. Basalaev, A. Bassalat, R. L. Bates, S. J. Batista, J. R. Batley, M. Battaglia, M. Bauce, F. Bauer, H. S. Bawa, J. B. Beacham, M. D. Beattie, T. Beau, P. H. Beauchemin, P. Bechtle, H. C. Beck, H. P. Beck, K. Becker, M. Becker, C. Becot, A. Beddall, A. J. Beddall, V. A. Bednyakov, M. Bedognetti, C. P. Bee, T. A. Beermann, M. Begalli, M. Begel, J. K. Behr, A. S. Bell, G. Bella, L. Bellagamba, A. Bellerive, M. Bellomo, K. Belotskiy, O. Beltramello, N. L. Belyaev, O. Benary, D. Benchekroun, M. Bender, K. Bendtz, N. Benekos, Y. Benhammou, E. Benhar Noccioli, J. Benitez, D. P. Benjamin, M. Benoit, J. R. Bensinger, S. Bentvelsen, L. Beresford, M. Beretta, D. Berge, E. Bergeaas Kuutmann, N. Berger, J. Beringer, S. Berlendis, N. R. Bernard, G. Bernardi, C. Bernius, F. U. Bernlochner, T. Berry, P. Berta, C. Bertella, G. Bertoli, F. Bertolucci, I. A. Bertram, C. Bertsche, D. Bertsche, G. J. Besjes, O. Bessidskaia Bylund, M. Bessner, N. Besson, A. Bethani, S. Bethke, A. J. Bevan, J. Beyer, R. M. Bianchi, O. Biebel, D. Biedermann, R. Bielski, K. Bierwagen, N. V. Biesuz, M. Biglietti, T. R. V. Billoud, H. Bilokon, M. Bindi, A. Bingul, C. Bini, S. Biondi, T. Bisanz, C. Bittrich, D. M. Bjergaard, J. E. Black, K. M. Black, R. E. Blair, T. Blazek, I. Bloch, C. Blocker, A. Blue, W. Blum, U. Blumenschein, Dr. Blunier, G. J. Bobbink, V. S. Bobrovnikov, S. S. Bocchetta, A. Bocci, C. Bock, M. Boehler, D. Boerner, D. Bogavac, A. G. Bogdanchikov, C. Bohm, V. Boisvert, P. Bokan, T. Bold, A. S. Boldyrev, A. E. Bolz, M. Bomben, M. Bona, M. Boonekamp, A. Borisov, G. Borissov, J. Bortfeldt, D. Bortoletto, V. Bortolotto, D. Boscherini, M. Bosman, J. D. Bossio Sola, J. Boudreau, J. Bouffard, E. V. Bouhova-Thacker, D. Boumediene, C. Bourdarios, S. K. Boutle, A. Boveia, J. Boyd, I. R. Boyko, J. Bracinik, A. Brandt, G. Brandt, O. Brandt, U. Bratzler, B. Brau, J. E. Brau, W. D. Breaden Madden, K. Brendlinger, A. J. Brennan, L. Brenner, R. Brenner, S. Bressler, D. L. Briglin, T. M. Bristow, D. Britton, D. Britzger, I. Brock, R. Brock, G. Brooijmans, T. Brooks, W. K. Brooks, J. Brosamer, E. Brost, J. H Broughton, P. A. Bruckman de Renstrom, D. Bruncko, A. Bruni, G. Bruni, L. S. Bruni, B. H. Brunt, M. Bruschi, N. Bruscino, P. Bryant, L. Bryngemark, T. Buanes, Q. Buat, P. Buchholz, A. G. Buckley, I. A. Budagov, M. K. Bugge, F. Bührer, O. Bulekov, D. Bullock, T. J. Burch, S. Burdin, C. D. Burgard, A. M. Burger, B. Burghgrave, K. Burka, S. Burke, I. Burmeister, J. T. P. Burr, E. Busato, D. Büscher, V. Büscher, P. Bussey, J. M. Butler, C. M. Buttar, J. M. Butterworth, P. Butti, W. Buttinger, A. Buzatu, A. R. Buzykaev, S. Cabrera Urbán, D. Caforio, V. M. M. Cairo, O. Cakir, N. Calace, P. Calafiura, A. Calandri, G. Calderini, P. Calfayan, G. Callea, L. P. Caloba, S. Calvente Lopez, D. Calvet, S. Calvet, T. P. Calvet, R. Camacho Toro, S. Camarda, P. Camarri, D. Cameron, R. Caminal Armadans, C. Camincher, S. Campana, M. Campanelli, A. Camplani, A. Campoverde, V. Canale, M. Cano Bret, J. Cantero, T. Cao, M. D. M. Capeans Garrido, I. Caprini, M. Caprini, M. Capua, R. M. Carbone, R. Cardarelli, F. C. Cardillo, I. Carli, T. Carli, G. Carlino, B. T. Carlson, L. Carminati, R. M. D. Carney, S. Caron, E. Carquin, S. Carrá, G. D. Carrillo-Montoya, D. Casadei, M. P. Casado, M. Casolino, D. W. Casper, R. Castelijn, V. Castillo Gimenez, N. F. Castro, A. Catinaccio, J. R. Catmore, A. Cattai, J. Caudron, V. Cavaliere, E. Cavallaro, D. Cavalli, M. Cavalli-Sforza, V. Cavasinni, E. Celebi, F. Ceradini, L. Cerda Alberich, A. S. Cerqueira, A. Cerri, L. Cerrito, F. Cerutti, A. Cervelli, S. A. Cetin, A. Chafaq, D. Chakraborty, S. K. Chan, W. S. Chan, Y. L. Chan, P. Chang, J. D. Chapman, D. G. Charlton, C. C. Chau, C. A. Chavez Barajas, S. Che, S. Cheatham, A. Chegwidden, S. Chekanov, S. V. Chekulaev, G. A. Chelkov, M. A. Chelstowska, C. H. Chen, H. Chen, J. Chen, S. Chen, S. J. Chen, X. Chen, Y. Chen, H. C. Cheng, H. J. Cheng, A. Cheplakov, E. Cheremushkina, R. Cherkaoui El Moursli, E. Cheu, K. Cheung, L. Chevalier, V. Chiarella, G. Chiarelli, G. Chiodini, A. S. Chisholm, A. Chitan, Y. H. Chiu, M. V. Chizhov, K. Choi, A. R. Chomont, S. Chouridou, Y. S. Chow, V. Christodoulou, M. C. Chu, J. Chudoba, A. J. Chuinard, J. J. Chwastowski, L. Chytka, A. K. Ciftci, D. Cinca, V. Cindro, I. A. Cioară, C. Ciocca, A. Ciocio, F. Cirotto, Z. H. Citron, M. Citterio, M. Ciubancan, A. Clark, B. L. Clark, M. R. Clark, P. J. Clark, R. N. Clarke, C. Clement, Y. Coadou, M. Cobal, A. Coccaro, J. Cochran, L. Colasurdo, B. Cole, A. P. Colijn, J. Collot, T. Colombo, P. Conde Muiño, E. Coniavitis, S. H. Connell, I. A. Connelly, S. Constantinescu, G. Conti, F. Conventi, M. Cooke, A. M. Cooper-Sarkar, F. Cormier, K. J. R. Cormier, M. Corradi, F. Corriveau, A. Cortes-Gonzalez, G. Cortiana, G. Costa, M. J. Costa, D. Costanzo, G. Cottin, G. Cowan, B. E. Cox, K. Cranmer, S. J. Crawley, R. A. Creager, G. Cree, S. Crépé-Renaudin, F. Crescioli, W. A. Cribbs, M. Cristinziani, V. Croft, G. Crosetti, A. Cueto, T. Cuhadar Donszelmann, A. R. Cukierman, J. Cummings, M. Curatolo, J. Cúth, S. Czekierda, P. Czodrowski, M. J. Da Cunha Sargedas De Sousa, C. Da Via, W. Dabrowski, T. Dado, T. Dai, O. Dale, F. Dallaire, C. Dallapiccola, M. Dam, G. D’amen, J. R. Dandoy, M. F. Daneri, N. P. Dang, A. C. Daniells, N. D. Dann, M. Danninger, M. Dano Hoffmann, V. Dao, G. Darbo, S. Darmora, J. Dassoulas, A. Dattagupta, T. Daubney, S. D’Auria, W. Davey, C. David, T. Davidek, D. R. Davis, P. Davison, E. Dawe, I. Dawson, K. De, R. De Asmundis, A. De Benedetti, S. De Castro, S. De Cecco, N. De Groot, P. de Jong, H. De la Torre, F. De Lorenzi, A. De Maria, D. De Pedis, A. De Salvo, U. De Sanctis, A. De Santo, K. De Vasconcelos Corga, J. B. De Vivie De Regie, R. Debbe, C. Debenedetti, D. V. Dedovich, N. Dehghanian, I. Deigaard, M. Del Gaudio, J. Del Peso, D. Delgove, F. Deliot, C. M. Delitzsch, M. Della Pietra, D. Della Volpe, A. Dell’Acqua, L. Dell’Asta, M. Dell’Orso, M. Delmastro, C. Delporte, P. A. Delsart, D. A. DeMarco, S. Demers, M. Demichev, A. Demilly, S. P. Denisov, D. Denysiuk, L. D’Eramo, D. Derendarz, J. E. Derkaoui, F. Derue, P. Dervan, K. Desch, C. Deterre, K. Dette, M. R. Devesa, P. O. Deviveiros, A. Dewhurst, S. Dhaliwal, F. A. Di Bello, A. Di Ciaccio, L. Di Ciaccio, W. K. Di Clemente, C. Di Donato, A. Di Girolamo, B. Di Girolamo, B. Di Micco, R. Di Nardo, K. F. Di Petrillo, A. Di Simone, R. Di Sipio, D. Di Valentino, C. Diaconu, M. Diamond, F. A. Dias, M. A. Diaz, E. B. Diehl, J. Dietrich, S. Díez Cornell, A. Dimitrievska, J. Dingfelder, P. Dita, S. Dita, F. Dittus, F. Djama, T. Djobava, J. I. Djuvsland, M. A. B. Do Vale, D. Dobos, M. Dobre, C. Doglioni, J. Dolejsi, Z. Dolezal, M. Donadelli, S. Donati, P. Dondero, J. Donini, M. D’Onofrio, J. Dopke, A. Doria, M. T. Dova, A. T. Doyle, E. Drechsler, M. Dris, Y. Du, J. Duarte-Campderros, A. Dubreuil, E. Duchovni, G. Duckeck, A. Ducourthial, O. A. Ducu, D. Duda, A. Dudarev, A. C. Dudder, E. M. Duffield, L. Duflot, M. Dührssen, C. Dülsen, M. Dumancic, A. E. Dumitriu, A. K. Duncan, M. Dunford, H. Duran Yildiz, M. Düren, A. Durglishvili, D. Duschinger, B. Dutta, D. Duvnjak, M. Dyndal, B. S. Dziedzic, C. Eckardt, K. M. Ecker, R. C. Edgar, T. Eifert, G. Eigen, K. Einsweiler, T. Ekelof, M. El Kacimi, R. El Kosseifi, V. Ellajosyula, M. Ellert, S. Elles, F. Ellinghaus, A. A. Elliot, N. Ellis, J. Elmsheuser, M. Elsing, D. Emeliyanov, Y. Enari, O. C. Endner, J. S. Ennis, J. Erdmann, A. Ereditato, M. Ernst, S. Errede, M. Escalier, C. Escobar, B. Esposito, O. Estrada Pastor, A. I. Etienvre, E. Etzion, H. Evans, A. Ezhilov, M. Ezzi, F. Fabbri, L. Fabbri, V. Fabiani, G. Facini, R. M. Fakhrutdinov, S. Falciano, R. J. Falla, J. Faltova, Y. Fang, M. Fanti, A. Farbin, A. Farilla, C. Farina, E. M. Farina, T. Farooque, S. Farrell, S. M. Farrington, P. Farthouat, F. Fassi, P. Fassnacht, D. Fassouliotis, M. Faucci Giannelli, A. Favareto, W. J. Fawcett, L. Fayard, O. L. Fedin, W. Fedorko, S. Feigl, L. Feligioni, C. Feng, E. J. Feng, H. Feng, M. J. Fenton, A. B. Fenyuk, L. Feremenga, P. Fernandez Martinez, S. Fernandez Perez, J. Ferrando, A. Ferrari, P. Ferrari, R. Ferrari, D. E. Ferreira de Lima, A. Ferrer, D. Ferrere, C. Ferretti, F. Fiedler, M. Filipuzzi, A. Filipčič, F. Filthaut, M. Fincke-Keeler, K. D. Finelli, M. C. N. Fiolhais, L. Fiorini, A. Fischer, C. Fischer, J. Fischer, W. C. Fisher, N. Flaschel, I. Fleck, P. Fleischmann, R. R. M. Fletcher, T. Flick, B. M. Flierl, L. R. Flores Castillo, M. J. Flowerdew, G. T. Forcolin, A. Formica, F. A. Förster, A. C. Forti, A. G. Foster, D. Fournier, H. Fox, S. Fracchia, P. Francavilla, M. Franchini, S. Franchino, D. Francis, L. Franconi, M. Franklin, M. Frate, M. Fraternali, D. Freeborn, S. M. Fressard-Batraneanu, B. Freund, D. Froidevaux, J. A. Frost, C. Fukunaga, T. Fusayasu, J. Fuster, C. Gabaldon, O. Gabizon, A. Gabrielli, A. Gabrielli, G. P. Gach, S. Gadatsch, S. Gadomski, G. Gagliardi, L. G. Gagnon, C. Galea, B. Galhardo, E. J. Gallas, B. J. Gallop, P. Gallus, G. Galster, K. K. Gan, S. Ganguly, Y. Gao, Y. S. Gao, C. García, J. E. García Navarro, J. A. García Pascual, M. Garcia-Sciveres, R. W. Gardner, N. Garelli, V. Garonne, A. Gascon Bravo, K. Gasnikova, C. Gatti, A. Gaudiello, G. Gaudio, I. L. Gavrilenko, C. Gay, G. Gaycken, E. N. Gazis, C. N. P. Gee, J. Geisen, M. Geisen, M. P. Geisler, K. Gellerstedt, C. Gemme, M. H. Genest, C. Geng, S. Gentile, C. Gentsos, S. George, D. Gerbaudo, A. Gershon, G. Gessner, S. Ghasemi, M. Ghneimat, B. Giacobbe, S. Giagu, N. Giangiacomi, P. Giannetti, S. M. Gibson, M. Gignac, M. Gilchriese, D. Gillberg, G. Gilles, D. M. Gingrich, M. P. Giordani, F. M. Giorgi, P. F. Giraud, P. Giromini, G. Giugliarelli, D. Giugni, F. Giuli, C. Giuliani, M. Giulini, B. K. Gjelsten, S. Gkaitatzis, I. Gkialas, E. L. Gkougkousis, P. Gkountoumis, L. K. Gladilin, C. Glasman, J. Glatzer, P. C. F. Glaysher, A. Glazov, M. Goblirsch-Kolb, J. Godlewski, S. Goldfarb, T. Golling, D. Golubkov, A. Gomes, R. Goncalves Gama, J. Goncalves Pinto Firmino Da Costa, R. Gonçalo, G. Gonella, L. Gonella, A. Gongadze, S. González de la Hoz, S. Gonzalez-Sevilla, L. Goossens, P. A. Gorbounov, H. A. Gordon, I. Gorelov, B. Gorini, E. Gorini, A. Gorišek, A. T. Goshaw, C. Gössling, M. I. Gostkin, C. A. Gottardo, C. R. Goudet, D. Goujdami, A. G. Goussiou, N. Govender, E. Gozani, L. Graber, I. Grabowska-Bold, P. O. J. Gradin, J. Gramling, E. Gramstad, S. Grancagnolo, V. Gratchev, P. M. Gravila, C. Gray, H. M. Gray, Z. D. Greenwood, C. Grefe, K. Gregersen, I. M. Gregor, P. Grenier, K. Grevtsov, J. Griffiths, A. A. Grillo, K. Grimm, S. Grinstein, Ph. Gris, J.-F. Grivaz, S. Groh, E. Gross, J. Grosse-Knetter, G. C. Grossi, Z. J. Grout, A. Grummer, L. Guan, W. Guan, J. Guenther, F. Guescini, D. Guest, O. Gueta, B. Gui, E. Guido, T. Guillemin, S. Guindon, U. Gul, C. Gumpert, J. Guo, W. Guo, Y. Guo, R. Gupta, S. Gupta, G. Gustavino, B. J. Gutelman, P. Gutierrez, N. G. Gutierrez Ortiz, C. Gutschow, C. Guyot, M. P. Guzik, C. Gwenlan, C. B. Gwilliam, A. Haas, C. Haber, H. K. Hadavand, N. Haddad, A. Hadef, S. Hageböck, M. Hagihara, H. Hakobyan, M. Haleem, J. Haley, G. Halladjian, G. D. Hallewell, K. Hamacher, P. Hamal, K. Hamano, A. Hamilton, G. N. Hamity, P. G. Hamnett, L. Han, S. Han, K. Hanagaki, K. Hanawa, M. Hance, B. Haney, P. Hanke, J. B. Hansen, J. D. Hansen, M. C. Hansen, P. H. Hansen, K. Hara, A. S. Hard, T. Harenberg, F. Hariri, S. Harkusha, R. D. Harrington, P. F. Harrison, N. M. Hartmann, Y. Hasegawa, A. Hasib, S. Hassani, S. Haug, R. Hauser, L. Hauswald, L. B. Havener, M. Havranek, C. M. Hawkes, R. J. Hawkings, D. Hayakawa, D. Hayden, C. P. Hays, J. M. Hays, H. S. Hayward, S. J. Haywood, S. J. Head, T. Heck, V. Hedberg, L. Heelan, S. Heer, K. K. Heidegger, S. Heim, T. Heim, B. Heinemann, J. J. Heinrich, L. Heinrich, C. Heinz, J. Hejbal, L. Helary, A. Held, S. Hellman, C. Helsens, R. C. W. Henderson, Y. Heng, S. Henkelmann, A. M. Henriques Correia, S. Henrot-Versille, G. H. Herbert, H. Herde, V. Herget, Y. Hernández Jiménez, H. Herr, G. Herten, R. Hertenberger, L. Hervas, T. C. Herwig, G. G. Hesketh, N. P. Hessey, J. W. Hetherly, S. Higashino, E. Higón-Rodriguez, K. Hildebrand, E. Hill, J. C. Hill, K. H. Hiller, S. J. Hillier, M. Hils, I. Hinchliffe, M. Hirose, D. Hirschbuehl, B. Hiti, O. Hladik, X. Hoad, J. Hobbs, N. Hod, M. C. Hodgkinson, P. Hodgson, A. Hoecker, M. R. Hoeferkamp, F. Hoenig, D. Hohn, T. R. Holmes, M. Homann, S. Honda, T. Honda, T. M. Hong, B. H. Hooberman, W. H. Hopkins, Y. Horii, A. J. Horton, J-Y. Hostachy, S. Hou, A. Hoummada, J. Howarth, J. Hoya, M. Hrabovsky, J. Hrdinka, I. Hristova, J. Hrivnac, A. Hrynevich, T. Hryn’ova, P. J. Hsu, S.-C. Hsu, Q. Hu, S. Hu, Y. Huang, Z. Hubacek, F. Hubaut, F. Huegging, T. B. Huffman, E. W. Hughes, G. Hughes, M. Huhtinen, P. Huo, N. Huseynov, J. Huston, J. Huth, G. Iacobucci, G. Iakovidis, I. Ibragimov, L. Iconomidou-Fayard, Z. Idrissi, P. Iengo, O. Igonkina, T. Iizawa, Y. Ikegami, M. Ikeno, Y. Ilchenko, D. Iliadis, N. Ilic, G. Introzzi, P. Ioannou, M. Iodice, K. Iordanidou, V. Ippolito, M. F. Isacson, N. Ishijima, M. Ishino, M. Ishitsuka, C. Issever, S. Istin, F. Ito, J. M. Iturbe Ponce, R. Iuppa, H. Iwasaki, J. M. Izen, V. Izzo, S. Jabbar, P. Jackson, R. M. Jacobs, V. Jain, K. B. Jakobi, K. Jakobs, S. Jakobsen, T. Jakoubek, D. O. Jamin, D. K. Jana, R. Jansky, J. Janssen, M. Janus, P. A. Janus, G. Jarlskog, N. Javadov, T. Javůrek, M. Javurkova, F. Jeanneau, L. Jeanty, J. Jejelava, A. Jelinskas, P. Jenni, C. Jeske, S. Jézéquel, H. Ji, J. Jia, H. Jiang, Y. Jiang, Z. Jiang, S. Jiggins, J. Jimenez Pena, S. Jin, A. Jinaru, O. Jinnouchi, H. Jivan, P. Johansson, K. A. Johns, C. A. Johnson, W. J. Johnson, K. Jon-And, R. W. L. Jones, S. D. Jones, S. Jones, T. J. Jones, J. Jongmanns, P. M. Jorge, J. Jovicevic, X. Ju, A. Juste Rozas, A. Kaczmarska, M. Kado, H. Kagan, M. Kagan, S. J. Kahn, T. Kaji, E. Kajomovitz, C. W. Kalderon, A. Kaluza, S. Kama, A. Kamenshchikov, N. Kanaya, L. Kanjir, V. A. Kantserov, J. Kanzaki, B. Kaplan, L. S. Kaplan, D. Kar, K. Karakostas, N. Karastathis, M. J. Kareem, E. Karentzos, S. N. Karpov, Z. M. Karpova, K. Karthik, V. Kartvelishvili, A. N. Karyukhin, K. Kasahara, L. Kashif, R. D. Kass, A. Kastanas, Y. Kataoka, C. Kato, A. Katre, J. Katzy, K. Kawade, K. Kawagoe, T. Kawamoto, G. Kawamura, E. F. Kay, V. F. Kazanin, R. Keeler, R. Kehoe, J. S. Keller, E. Kellermann, J. J. Kempster, J Kendrick, H. Keoshkerian, O. Kepka, S. Kersten, B. P. Kerševan, R. A. Keyes, M. Khader, F. Khalil-Zada, A. Khanov, A. G. Kharlamov, T. Kharlamova, A. Khodinov, T. J. Khoo, V. Khovanskiy, E. Khramov, J. Khubua, S. Kido, C. R. Kilby, H. Y. Kim, S. H. Kim, Y. K. Kim, N. Kimura, O. M. Kind, B. T. King, D. Kirchmeier, J. Kirk, A. E. Kiryunin, T. Kishimoto, D. Kisielewska, V. Kitali, O. Kivernyk, E. Kladiva, T. Klapdor-Kleingrothaus, M. H. Klein, M. Klein, U. Klein, K. Kleinknecht, P. Klimek, A. Klimentov, R. Klingenberg, T. Klingl, T. Klioutchnikova, P. Kluit, S. Kluth, E. Kneringer, E. B. F. G. Knoops, A. Knue, A. Kobayashi, D. Kobayashi, T. Kobayashi, M. Kobel, M. Kocian, P. Kodys, T. Koffas, E. Koffeman, M. K. Köhler, N. M. Köhler, T. Koi, M. Kolb, I. Koletsou, A. A. Komar, T. Kondo, N. Kondrashova, K. Köneke, A. C. König, T. Kono, R. Konoplich, N. Konstantinidis, R. Kopeliansky, S. Koperny, A. K. Kopp, K. Korcyl, K. Kordas, A. Korn, A. A. Korol, I. Korolkov, E. V. Korolkova, O. Kortner, S. Kortner, T. Kosek, V. V. Kostyukhin, A. Kotwal, A. Koulouris, A. Kourkoumeli-Charalampidi, C. Kourkoumelis, E. Kourlitis, V. Kouskoura, A. B. Kowalewska, R. Kowalewski, T. Z. Kowalski, C. Kozakai, W. Kozanecki, A. S. Kozhin, V. A. Kramarenko, G. Kramberger, D. Krasnopevtsev, M. W. Krasny, A. Krasznahorkay, D. Krauss, J. A. Kremer, J. Kretzschmar, K. Kreutzfeldt, P. Krieger, K. Krizka, K. Kroeninger, H. Kroha, J. Kroll, J. Kroll, J. Kroseberg, J. Krstic, U. Kruchonak, H. Krüger, N. Krumnack, M. C. Kruse, T. Kubota, H. Kucuk, S. Kuday, J. T. Kuechler, S. Kuehn, A. Kugel, F. Kuger, T. Kuhl, V. Kukhtin, R. Kukla, Y. Kulchitsky, S. Kuleshov, Y. P. Kulinich, M. Kuna, T. Kunigo, A. Kupco, T. Kupfer, O. Kuprash, H. Kurashige, L. L. Kurchaninov, Y. A. Kurochkin, M. G. Kurth, V. Kus, E. S. Kuwertz, M. Kuze, J. Kvita, T. Kwan, D. Kyriazopoulos, A. La Rosa, J. L. La Rosa Navarro, L. La Rotonda, F. La Ruffa, C. Lacasta, F. Lacava, J. Lacey, D. P. J. Lack, H. Lacker, D. Lacour, E. Ladygin, R. Lafaye, B. Laforge, S. Lai, S. Lammers, W. Lampl, E. Lançon, U. Landgraf, M. P. J. Landon, M. C. Lanfermann, V. S. Lang, J. C. Lange, R. J. Langenberg, A. J. Lankford, F. Lanni, K. Lantzsch, A. Lanza, A. Lapertosa, S. Laplace, J. F. Laporte, T. Lari, F. Lasagni Manghi, M. Lassnig, T. S. Lau, P. Laurelli, W. Lavrijsen, A. T. Law, P. Laycock, T. Lazovich, M. Lazzaroni, B. Le, O. Le Dortz, E. Le Guirriec, E. P. Le Quilleuc, M. LeBlanc, T. LeCompte, F. Ledroit-Guillon, C. A. Lee, G. R. Lee, L. Lee, S. C. Lee, B. Lefebvre, G. Lefebvre, M. Lefebvre, F. Legger, C. Leggett, G. Lehmann Miotto, X. Lei, W. A. Leight, M. A. L. Leite, R. Leitner, D. Lellouch, B. Lemmer, K. J. C. Leney, T. Lenz, B. Lenzi, R. Leone, S. Leone, C. Leonidopoulos, G. Lerner, C. Leroy, A. A. J. Lesage, C. G. Lester, M. Levchenko, J. Levêque, D. Levin, L. J. Levinson, M. Levy, D. Lewis, B. Li, C-Q. Li, H. Li, L. Li, Q. Li, Q. Y. Li, S. Li, X. Li, Y. Li, Z. Liang, B. Liberti, A. Liblong, K. Lie, J. Liebal, W. Liebig, A. Limosani, S. C. Lin, T. H. Lin, R. A. Linck, B. E. Lindquist, A. L. Lionti, E. Lipeles, A. Lipniacka, M. Lisovyi, T. M. Liss, A. Lister, A. M. Litke, B. Liu, H. B. Liu, H. Liu, J. B. Liu, J. K. K. Liu, J. Liu, K. Liu, L. Liu, M. Liu, Y. L. Liu, Y. W. Liu, M. Livan, A. Lleres, J. Llorente Merino, S. L. Lloyd, C. Y. Lo, F. Lo Sterzo, E. M. Lobodzinska, P. Loch, F. K. Loebinger, K. M. Loew, A. Loginov, T. Lohse, K. Lohwasser, M. Lokajicek, B. A. Long, J. D. Long, R. E. Long, L. Longo, K. A. Looper, J. A. Lopez, D. Lopez Mateos, I. Lopez Paz, A. Lopez Solis, J. Lorenz, N. Lorenzo Martinez, M. Losada, P. J. Lösel, A. Lösle, X. Lou, A. Lounis, J. Love, P. A. Love, H. Lu, N. Lu, Y. J. Lu, H. J. Lubatti, C. Luci, A. Lucotte, C. Luedtke, F. Luehring, W. Lukas, L. Luminari, O. Lundberg, B. Lund-Jensen, M. S. Lutz, P. M. Luzi, D. Lynn, R. Lysak, E. Lytken, F. Lyu, V. Lyubushkin, H. Ma, L. L. Ma, Y. Ma, G. Maccarrone, A. Macchiolo, C. M. Macdonald, J. Machado Miguens, D. Madaffari, R. Madar, W. F. Mader, A. Madsen, J. Maeda, S. Maeland, T. Maeno, A. S. Maevskiy, V. Magerl, J. Mahlstedt, C. Maiani, C. Maidantchik, A. A. Maier, T. Maier, A. Maio, O. Majersky, S. Majewski, Y. Makida, N. Makovec, B. Malaescu, Pa. Malecki, V. P. Maleev, F. Malek, U. Mallik, D. Malon, C. Malone, S. Maltezos, S. Malyukov, J. Mamuzic, G. Mancini, I. Mandić, J. Maneira, L. Manhaes de Andrade Filho, J. Manjarres Ramos, K. H. Mankinen, A. Mann, A. Manousos, B. Mansoulie, J. D. Mansour, R. Mantifel, M. Mantoani, S. Manzoni, L. Mapelli, G. Marceca, L. March, L. Marchese, G. Marchiori, M. Marcisovsky, M. Marjanovic, D. E. Marley, F. Marroquim, S. P. Marsden, Z. Marshall, M. U. F Martensson, S. Marti-Garcia, C. B. Martin, T. A. Martin, V. J. Martin, B. Martin dit Latour, M. Martinez, V. I. Martinez Outschoorn, S. Martin-Haugh, V. S. Martoiu, A. C. Martyniuk, A. Marzin, L. Masetti, T. Mashimo, R. Mashinistov, J. Masik, A. L. Maslennikov, L. Massa, P. Mastrandrea, A. Mastroberardino, T. Masubuchi, P. Mättig, J. Maurer, B. Maček, S. J. Maxfield, D. A. Maximov, R. Mazini, I. Maznas, S. M. Mazza, N. C. Mc Fadden, G. Mc Goldrick, S. P. Mc Kee, A. McCarn, R. L. McCarthy, T. G. McCarthy, L. I. McClymont, E. F. McDonald, J. A. Mcfayden, G. Mchedlidze, S. J. McMahon, P. C. McNamara, C. J. McNicol, R. A. McPherson, S. Meehan, T. M. Megy, S. Mehlhase, A. Mehta, T. Meideck, B. Meirose, D. Melini, B. R. Mellado Garcia, J. D. Mellenthin, M. Melo, F. Meloni, A. Melzer, S. B. Menary, L. Meng, X. T. Meng, A. Mengarelli, S. Menke, E. Meoni, S. Mergelmeyer, C. Merlassino, P. Mermod, L. Merola, C. Meroni, F. S. Merritt, A. Messina, J. Metcalfe, A. S. Mete, C. Meyer, J. Meyer, J-P. Meyer, H. Meyer Zu Theenhausen, F. Miano, R. P. Middleton, S. Miglioranzi, L. Mijović, G. Mikenberg, M. Mikestikova, M. Mikuž, M. Milesi, A. Milic, D. A. Millar, D. W. Miller, C. Mills, A. Milov, D. A. Milstead, A. A. Minaenko, Y. Minami, I. A. Minashvili, A. I. Mincer, B. Mindur, M. Mineev, Y. Minegishi, Y. Ming, L. M. Mir, K. P. Mistry, T. Mitani, J. Mitrevski, V. A. Mitsou, A. Miucci, P. S. Miyagawa, A. Mizukami, J. U. Mjörnmark, T. Mkrtchyan, M. Mlynarikova, T. Moa, K. Mochizuki, P. Mogg, S. Mohapatra, S. Molander, R. Moles-Valls, M. C. Mondragon, K. Mönig, J. Monk, E. Monnier, A. Montalbano, J. Montejo Berlingen, F. Monticelli, S. Monzani, R. W. Moore, N. Morange, D. Moreno, M. Moreno Llácer, P. Morettini, S. Morgenstern, D. Mori, T. Mori, M. Morii, M. Morinaga, V. Morisbak, A. K. Morley, G. Mornacchi, J. D. Morris, L. Morvaj, P. Moschovakos, M. Mosidze, H. J. Moss, J. Moss, K. Motohashi, R. Mount, E. Mountricha, E. J. W. Moyse, S. Muanza, F. Mueller, J. Mueller, R. S. P. Mueller, D. Muenstermann, P. Mullen, G. A. Mullier, F. J. Munoz Sanchez, W. J. Murray, H. Musheghyan, M. Muškinja, A. G. Myagkov, M. Myska, B. P. Nachman, O. Nackenhorst, K. Nagai, R. Nagai, K. Nagano, Y. Nagasaka, K. Nagata, M. Nagel, E. Nagy, A. M. Nairz, Y. Nakahama, K. Nakamura, T. Nakamura, I. Nakano, R. F. Naranjo Garcia, R. Narayan, D. I. Narrias Villar, I. Naryshkin, T. Naumann, G. Navarro, R. Nayyar, H. A. Neal, P. Y. Nechaeva, T. J. Neep, A. Negri, M. Negrini, S. Nektarijevic, C. Nellist, A. Nelson, M. E. Nelson, S. Nemecek, P. Nemethy, M. Nessi, M. S. Neubauer, M. Neumann, P. R. Newman, T. Y. Ng, T. Nguyen Manh, R. B. Nickerson, R. Nicolaidou, J. Nielsen, V. Nikolaenko, I. Nikolic-Audit, K. Nikolopoulos, J. K. Nilsen, P. Nilsson, Y. Ninomiya, A. Nisati, N. Nishu, R. Nisius, I. Nitsche, T. Nitta, T. Nobe, Y. Noguchi, M. Nomachi, I. Nomidis, M. A. Nomura, T. Nooney, M. Nordberg, N. Norjoharuddeen, O. Novgorodova, M. Nozaki, L. Nozka, K. Ntekas, E. Nurse, F. Nuti, F. G. Oakham, H. Oberlack, T. Obermann, J. Ocariz, A. Ochi, I. Ochoa, J. P. Ochoa-Ricoux, K. O’Connor, S. Oda, S. Odaka, A. Oh, S. H. Oh, C. C. Ohm, H. Ohman, H. Oide, H. Okawa, Y. Okumura, T. Okuyama, A. Olariu, L. F. Oleiro Seabra, S. A. Olivares Pino, D. Oliveira Damazio, A. Olszewski, J. Olszowska, D. C. O’Neil, A. Onofre, K. Onogi, P. U. E. Onyisi, H. Oppen, M. J. Oreglia, Y. Oren, D. Orestano, N. Orlando, A. A. O’Rourke, R. S. Orr, B. Osculati, V. O’Shea, R. Ospanov, G. Otero y Garzon, H. Otono, M. Ouchrif, F. Ould-Saada, A. Ouraou, K. P. Oussoren, Q. Ouyang, M. Owen, R. E. Owen, V. E. Ozcan, N. Ozturk, K. Pachal, A. Pacheco Pages, L. Pacheco Rodriguez, C. Padilla Aranda, S. Pagan Griso, M. Paganini, F. Paige, G. Palacino, S. Palazzo, S. Palestini, M. Palka, D. Pallin, E. St. Panagiotopoulou, I. Panagoulias, C. E. Pandini, J. G. Panduro Vazquez, P. Pani, S. Panitkin, D. Pantea, L. Paolozzi, T. D. Papadopoulou, K. Papageorgiou, A. Paramonov, D. Paredes Hernandez, A. J. Parker, K. A. Parker, M. A. Parker, F. Parodi, J. A. Parsons, U. Parzefall, V. R. Pascuzzi, J. M. P. Pasner, E. Pasqualucci, S. Passaggio, F. Pastore, S. Pataraia, J. R. Pater, T. Pauly, B. Pearson, S. Pedraza Lopez, R. Pedro, S. V. Peleganchuk, O. Penc, C. Peng, H. Peng, J. Penwell, B. S. Peralva, M. M. Perego, D. V. Perepelitsa, F. Peri, L. Perini, H. Pernegger, S. Perrella, R. Peschke, V. D. Peshekhonov, K. Peters, R. F. Y. Peters, B. A. Petersen, T. C. Petersen, E. Petit, A. Petridis, C. Petridou, P. Petroff, E. Petrolo, M. Petrov, F. Petrucci, N. E. Pettersson, A. Peyaud, R. Pezoa, F. H. Phillips, P. W. Phillips, G. Piacquadio, E. Pianori, A. Picazio, E. Piccaro, M. A. Pickering, R. Piegaia, J. E. Pilcher, A. D. Pilkington, A. W. J. Pin, M. Pinamonti, J. L. Pinfold, H. Pirumov, M. Pitt, L. Plazak, M.-A. Pleier, V. Pleskot, E. Plotnikova, D. Pluth, P. Podberezko, R. Poettgen, R. Poggi, L. Poggioli, I. Pogrebnyak, D. Pohl, G. Polesello, A. Poley, A. Policicchio, R. Polifka, A. Polini, C. S. Pollard, V. Polychronakos, K. Pommès, D. Ponomarenko, L. Pontecorvo, G. A. Popeneciu, S. Pospisil, K. Potamianos, I. N. Potrap, C. J. Potter, H. Potti, T. Poulsen, J. Poveda, M. E. Pozo Astigarraga, P. Pralavorio, A. Pranko, S. Prell, D. Price, M. Primavera, S. Prince, N. Proklova, K. Prokofiev, F. Prokoshin, S. Protopopescu, J. Proudfoot, M. Przybycien, A. Puri, P. Puzo, J. Qian, G. Qin, Y. Qin, A. Quadt, M. Queitsch-Maitland, D. Quilty, S. Raddum, V. Radeka, V. Radescu, S. K. Radhakrishnan, P. Radloff, P. Rados, F. Ragusa, G. Rahal, J. A. Raine, S. Rajagopalan, C. Rangel-Smith, T. Rashid, S. Raspopov, M. G. Ratti, D. M. Rauch, F. Rauscher, S. Rave, I. Ravinovich, J. H. Rawling, M. Raymond, A. L. Read, N. P. Readioff, M. Reale, D. M. Rebuzzi, A. Redelbach, G. Redlinger, R. Reece, R. G. Reed, K. Reeves, L. Rehnisch, J. Reichert, A. Reiss, C. Rembser, H. Ren, M. Rescigno, S. Resconi, E. D. Resseguie, S. Rettie, E. Reynolds, O. L. Rezanova, P. Reznicek, R. Rezvani, R. Richter, S. Richter, E. Richter-Was, O. Ricken, M. Ridel, P. Rieck, C. J. Riegel, J. Rieger, O. Rifki, M. Rijssenbeek, A. Rimoldi, M. Rimoldi, L. Rinaldi, G. Ripellino, B. Ristić, E. Ritsch, I. Riu, F. Rizatdinova, E. Rizvi, C. Rizzi, R. T. Roberts, S. H. Robertson, A. Robichaud-Veronneau, D. Robinson, J. E. M. Robinson, A. Robson, E. Rocco, C. Roda, Y. Rodina, S. Rodriguez Bosca, A. Rodriguez Perez, D. Rodriguez Rodriguez, S. Roe, C. S. Rogan, O. Røhne, J. Roloff, A. Romaniouk, M. Romano, S. M. Romano Saez, E. Romero Adam, N. Rompotis, M. Ronzani, L. Roos, S. Rosati, K. Rosbach, P. Rose, N-A. Rosien, E. Rossi, L. P. Rossi, J. H. N. Rosten, R. Rosten, M. Rotaru, J. Rothberg, D. Rousseau, A. Rozanov, Y. Rozen, X. Ruan, F. Rubbo, F. Rühr, A. Ruiz-Martinez, Z. Rurikova, N. A. Rusakovich, H. L. Russell, J. P. Rutherfoord, N. Ruthmann, Y. F. Ryabov, M. Rybar, G. Rybkin, S. Ryu, A. Ryzhov, G. F. Rzehorz, A. F. Saavedra, G. Sabato, S. Sacerdoti, H. F-W. Sadrozinski, R. Sadykov, F. Safai Tehrani, P. Saha, M. Sahinsoy, M. Saimpert, M. Saito, T. Saito, H. Sakamoto, Y. Sakurai, G. Salamanna, J. E. Salazar Loyola, D. Salek, P. H. Sales De Bruin, D. Salihagic, A. Salnikov, J. Salt, D. Salvatore, F. Salvatore, A. Salvucci, A. Salzburger, D. Sammel, D. Sampsonidis, D. Sampsonidou, J. Sánchez, V. Sanchez Martinez, A. Sanchez Pineda, H. Sandaker, R. L. Sandbach, C. O. Sander, M. Sandhoff, C. Sandoval, D. P. C. Sankey, M. Sannino, Y. Sano, A. Sansoni, C. Santoni, H. Santos, I. Santoyo Castillo, A. Sapronov, J. G. Saraiva, B. Sarrazin, O. Sasaki, K. Sato, E. Sauvan, G. Savage, P. Savard, N. Savic, C. Sawyer, L. Sawyer, J. Saxon, C. Sbarra, A. Sbrizzi, T. Scanlon, D. A. Scannicchio, J. Schaarschmidt, P. Schacht, B. M. Schachtner, D. Schaefer, L. Schaefer, R. Schaefer, J. Schaeffer, S. Schaepe, S. Schaetzel, U. Schäfer, A. C. Schaffer, D. Schaile, R. D. Schamberger, V. A. Schegelsky, D. Scheirich, M. Schernau, C. Schiavi, S. Schier, L. K. Schildgen, C. Schillo, M. Schioppa, S. Schlenker, K. R. Schmidt-Sommerfeld, K. Schmieden, C. Schmitt, S. Schmitt, S. Schmitz, U. Schnoor, L. Schoeffel, A. Schoening, B. D. Schoenrock, E. Schopf, M. Schott, J. F. P. Schouwenberg, J. Schovancova, S. Schramm, N. Schuh, A. Schulte, M. J. Schultens, H-C. Schultz-Coulon, H. Schulz, M. Schumacher, B. A. Schumm, Ph. Schune, A. Schwartzman, T. A. Schwarz, H. Schweiger, Ph. Schwemling, R. Schwienhorst, A. Sciandra, G. Sciolla, M. Scornajenghi, F. Scuri, F. Scutti, J. Searcy, P. Seema, S. C. Seidel, A. Seiden, J. M. Seixas, G. Sekhniaidze, K. Sekhon, S. J. Sekula, N. Semprini-Cesari, S. Senkin, C. Serfon, L. Serin, L. Serkin, M. Sessa, R. Seuster, H. Severini, F. Sforza, A. Sfyrla, E. Shabalina, N. W. Shaikh, L. Y. Shan, R. Shang, J. T. Shank, M. Shapiro, P. B. Shatalov, K. Shaw, S. M. Shaw, A. Shcherbakova, C. Y. Shehu, Y. Shen, N. Sherafati, P. Sherwood, L. Shi, S. Shimizu, C. O. Shimmin, M. Shimojima, I. P. J. Shipsey, S. Shirabe, M. Shiyakova, J. Shlomi, A. Shmeleva, D. Shoaleh Saadi, M. J. Shochet, S. Shojaii, D. R. Shope, S. Shrestha, E. Shulga, M. A. Shupe, P. Sicho, A. M. Sickles, P. E. Sidebo, E. Sideras Haddad, O. Sidiropoulou, A. Sidoti, F. Siegert, Dj. Sijacki, J. Silva, S. B. Silverstein, V. Simak, L. Simic, S. Simion, E. Simioni, B. Simmons, M. Simon, P. Sinervo, N. B. Sinev, M. Sioli, G. Siragusa, I. Siral, S. Yu. Sivoklokov, J. Sjölin, M. B. Skinner, P. Skubic, M. Slater, T. Slavicek, M. Slawinska, K. Sliwa, R. Slovak, V. Smakhtin, B. H. Smart, J. Smiesko, N. Smirnov, S. Yu. Smirnov, Y. Smirnov, L. N. Smirnova, O. Smirnova, J. W. Smith, M. N. K. Smith, R. W. Smith, M. Smizanska, K. Smolek, A. A. Snesarev, I. M. Snyder, S. Snyder, R. Sobie, F. Socher, A. Soffer, A. Søgaard, D. A. Soh, G. Sokhrannyi, C. A. Solans Sanchez, M. Solar, E. Yu. Soldatov, U. Soldevila, A. A. Solodkov, A. Soloshenko, O. V. Solovyanov, V. Solovyev, P. Sommer, H. Son, A. Sopczak, D. Sosa, C. L. Sotiropoulou, R. Soualah, A. M. Soukharev, D. South, B. C. Sowden, S. Spagnolo, M. Spalla, M. Spangenberg, F. Spanò, D. Sperlich, F. Spettel, T. M. Spieker, R. Spighi, G. Spigo, L. A. Spiller, M. Spousta, R. D. St. Denis, A. Stabile, R. Stamen, S. Stamm, E. Stanecka, R. W. Stanek, C. Stanescu, M. M. Stanitzki, B. Stapf, S. Stapnes, E. A. Starchenko, G. H. Stark, J. Stark, S. H Stark, P. Staroba, P. Starovoitov, S. Stärz, R. Staszewski, P. Steinberg, B. Stelzer, H. J. Stelzer, O. Stelzer-Chilton, H. Stenzel, G. A. Stewart, M. C. Stockton, M. Stoebe, G. Stoicea, P. Stolte, S. Stonjek, A. R. Stradling, A. Straessner, M. E. Stramaglia, J. Strandberg, S. Strandberg, M. Strauss, P. Strizenec, R. Ströhmer, D. M. Strom, R. Stroynowski, A. Strubig, S. A. Stucci, B. Stugu, N. A. Styles, D. Su, J. Su, S. Suchek, Y. Sugaya, M. Suk, V. V. Sulin, D. M. S. Sultan, S. Sultansoy, T. Sumida, S. Sun, X. Sun, K. Suruliz, C. J. E. Suster, M. R. Sutton, S. Suzuki, M. Svatos, M. Swiatlowski, S. P. Swift, I. Sykora, T. Sykora, D. Ta, K. Tackmann, J. Taenzer, A. Taffard, R. Tafirout, E. Tahirovic, N. Taiblum, H. Takai, R. Takashima, E. H. Takasugi, T. Takeshita, Y. Takubo, M. Talby, A. A. Talyshev, J. Tanaka, M. Tanaka, R. Tanaka, S. Tanaka, R. Tanioka, B. B. Tannenwald, S. Tapia Araya, S. Tapprogge, S. Tarem, G. F. Tartarelli, P. Tas, M. Tasevsky, T. Tashiro, E. Tassi, A. Tavares Delgado, Y. Tayalati, A. C. Taylor, A. J. Taylor, G. N. Taylor, P. T. E. Taylor, W. Taylor, P. Teixeira-Dias, D. Temple, H. Ten Kate, P. K. Teng, J. J. Teoh, F. Tepel, S. Terada, K. Terashi, J. Terron, S. Terzo, M. Testa, R. J. Teuscher, T. Theveneaux-Pelzer, F. Thiele, J. P. Thomas, J. Thomas-Wilsker, A. S. Thompson, P. D. Thompson, L. A. Thomsen, E. Thomson, M. J. Tibbetts, R. E. Ticse Torres, V. O. Tikhomirov, Yu. A. Tikhonov, S. Timoshenko, P. Tipton, S. Tisserant, K. Todome, S. Todorova-Nova, S. Todt, J. Tojo, S. Tokár, K. Tokushuku, E. Tolley, L. Tomlinson, M. Tomoto, L. Tompkins, K. Toms, B. Tong, P. Tornambe, E. Torrence, H. Torres, E. Torró Pastor, J. Toth, F. Touchard, D. R. Tovey, C. J. Treado, T. Trefzger, F. Tresoldi, A. Tricoli, I. M. Trigger, S. Trincaz-Duvoid, M. F. Tripiana, W. Trischuk, B. Trocmé, A. Trofymov, C. Troncon, M. Trottier-McDonald, M. Trovatelli, L. Truong, M. Trzebinski, A. Trzupek, K. W. Tsang, J. C-L. Tseng, P. V. Tsiareshka, G. Tsipolitis, N. Tsirintanis, S. Tsiskaridze, V. Tsiskaridze, E. G. Tskhadadze, K. M. Tsui, I. I. Tsukerman, V. Tsulaia, S. Tsuno, D. Tsybychev, Y. Tu, A. Tudorache, V. Tudorache, T. T. Tulbure, A. N. Tuna, S. A. Tupputi, S. Turchikhin, D. Turgeman, I. Turk Cakir, R. Turra, P. M. Tuts, G. Ucchielli, I. Ueda, M. Ughetto, F. Ukegawa, G. Unal, A. Undrus, G. Unel, F. C. Ungaro, Y. Unno, C. Unverdorben, J. Urban, P. Urquijo, P. Urrejola, G. Usai, J. Usui, L. Vacavant, V. Vacek, B. Vachon, K. O. H. Vadla, A. Vaidya, C. Valderanis, E. Valdes Santurio, M. Valente, S. Valentinetti, A. Valero, L. Valéry, S. Valkar, A. Vallier, J. A. Valls Ferrer, W. Van Den Wollenberg, H. Van der Graaf, P. Van Gemmeren, J. Van Nieuwkoop, I. Van Vulpen, M. C. van Woerden, M. Vanadia, W. Vandelli, A. Vaniachine, P. Vankov, G. Vardanyan, R. Vari, E. W. Varnes, C. Varni, T. Varol, D. Varouchas, A. Vartapetian, K. E. Varvell, G. A. Vasquez, J. G. Vasquez, F. Vazeille, T. Vazquez Schroeder, J. Veatch, V. Veeraraghavan, L. M. Veloce, F. Veloso, S. Veneziano, A. Ventura, M. Venturi, N. Venturi, A. Venturini, V. Vercesi, M. Verducci, W. Verkerke, A. T. Vermeulen, J. C. Vermeulen, M. C. Vetterli, N. Viaux Maira, O. Viazlo, I. Vichou, T. Vickey, O. E. Vickey Boeriu, G. H. A. Viehhauser, S. Viel, L. Vigani, M. Villa, M. Villaplana Perez, E. Vilucchi, M. G. Vincter, V. B. Vinogradov, A. Vishwakarma, C. Vittori, I. Vivarelli, S. Vlachos, M. Vogel, P. Vokac, G. Volpi, H. von der Schmitt, E. Von Toerne, V. Vorobel, K. Vorobev, M. Vos, R. Voss, J. H. Vossebeld, N. Vranjes, M. Vranjes Milosavljevic, V. Vrba, M. Vreeswijk, T. Šfiligoj, R. Vuillermet, I. Vukotic, T. Ženiš, L. Živković, P. Wagner, W. Wagner, J. Wagner-Kuhr, H. Wahlberg, S. Wahrmund, J. Walder, R. Walker, W. Walkowiak, V. Wallangen, C. Wang, C. Wang, F. Wang, H. Wang, H. Wang, J. Wang, J. Wang, Q. Wang, R. Wang, S. M. Wang, T. Wang, W. Wang, W. X. Wang, Z. Wang, C. Wanotayaroj, A. Warburton, C. P. Ward, D. R. Wardrope, A. Washbrook, P. M. Watkins, A. T. Watson, M. F. Watson, G. Watts, S. Watts, B. M. Waugh, A. F. Webb, S. Webb, M. S. Weber, S. A. Weber, S. W. Weber, J. S. Webster, A. R. Weidberg, B. Weinert, J. Weingarten, M. Weirich, C. Weiser, H. Weits, P. S. Wells, T. Wenaus, T. Wengler, S. Wenig, N. Wermes, M. D. Werner, P. Werner, M. Wessels, T. D. Weston, K. Whalen, N. L. Whallon, A. M. Wharton, A. S. White, A. White, M. J. White, R. White, D. Whiteson, B. W. Whitmore, F. J. Wickens, W. Wiedenmann, M. Wielers, C. Wiglesworth, L. A. M. Wiik-Fuchs, A. Wildauer, F. Wilk, H. G. Wilkens, H. H. Williams, S. Williams, C. Willis, S. Willocq, J. A. Wilson, I. Wingerter-Seez, E. Winkels, F. Winklmeier, O. J. Winston, B. T. Winter, M. Wittgen, M. Wobisch, T. M. H. Wolf, R. Wolff, M. W. Wolter, H. Wolters, V. W. S. Wong, S. D. Worm, B. K. Wosiek, J. Wotschack, K. W. Woźniak, M. Wu, S. L. Wu, X. Wu, Y. Wu, T. R. Wyatt, B. M. Wynne, S. Xella, Z. Xi, L. Xia, D. Xu, L. Xu, T. Xu, B. Yabsley, S. Yacoob, D. Yamaguchi, Y. Yamaguchi, A. Yamamoto, S. Yamamoto, T. Yamanaka, F. Yamane, M. Yamatani, Y. Yamazaki, Z. Yan, H. J. Yang, H. T. Yang, Y. Yang, Z. Yang, W-M. Yao, Y. C. Yap, Y. Yasu, E. Yatsenko, K. H. Yau Wong, J. Ye, S. Ye, I. Yeletskikh, E. Yigitbasi, E. Yildirim, K. Yorita, K. Yoshihara, C. J. S. Young, C. Young, J. Yu, J. Yu, S. P. Y. Yuen, I. Yusuff, B. Zabinski, G. Zacharis, R. Zaidan, A. M. Zaitsev, N. Zakharchuk, J. Zalieckas, A. Zaman, S. Zambito, D. Zanzi, C. Zeitnitz, G. Zemaityte, A. Zemla, J. C. Zeng, Q. Zeng, O. Zenin, D. Zerwas, D. Zhang, F. Zhang, G. Zhang, H. Zhang, J. Zhang, L. Zhang, L. Zhang, M. Zhang, P. Zhang, R. Zhang, R. Zhang, X. Zhang, Y. Zhang, Z. Zhang, X. Zhao, Y. Zhao, Z. Zhao, A. Zhemchugov, B. Zhou, C. Zhou, L. Zhou, M. S. Zhou, M. Zhou, N. Zhou, C. G. Zhu, H. Zhu, J. Zhu, Y. Zhu, X. Zhuang, K. Zhukov, A. Zibell, D. Zieminska, N. I. Zimine, C. Zimmermann, S. Zimmermann, Z. Zinonos, M. Zinser, M. Ziolkowski, G. Zobernig, A. Zoccoli, R. Zou, M. Zur Nedden, L. Zwalinski, ATLAS Collaboration

**Affiliations:** 1 Department of Physics, University of Adelaide, Adelaide, Australia; 2 Physics Department, SUNY Albany, Albany, NY USA; 3 Department of Physics, University of Alberta, Edmonton, AB Canada; 4 Department of Physics, Ankara University, Ankara, Turkey; 5 Istanbul Aydin University, Istanbul, Turkey; 6 Division of Physics, TOBB University of Economics and Technology, Ankara, Turkey; 7 LAPP, Université Grenoble Alpes, Université Savoie Mont Blanc, CNRS/IN2P3, Annecy, France; 8 High Energy Physics Division, Argonne National Laboratory, Argonne, IL USA; 9 Department of Physics, University of Arizona, Tucson, AZ USA; 10 Department of Physics, University of Texas at Arlington, Arlington, TX USA; 11 Physics Department, National and Kapodistrian University of Athens, Athens, Greece; 12 Physics Department, National Technical University of Athens, Zografou, Greece; 13 Department of Physics, University of Texas at Austin, Austin, TX USA; 14 Faculty of Engineering and Natural Sciences, Bahcesehir University, Istanbul, Turkey; 15 Faculty of Engineering and Natural Sciences, Istanbul Bilgi University, Istanbul, Turkey; 16 Department of Physics, Bogazici University, Istanbul, Turkey; 17 Department of Physics Engineering, Gaziantep University, Gaziantep, Turkey; 18 Institute of Physics, Azerbaijan Academy of Sciences, Baku, Azerbaijan; 19 Institut de Física d’Altes Energies (IFAE), Barcelona Institute of Science and Technology, Barcelona, Spain; 20 Institute of High Energy Physics, Chinese Academy of Sciences, Beijing, China; 21 Physics Department, Tsinghua University, Beijing, China; 22 Department of Physics, Nanjing University, Nanjing, China; 23 University of Chinese Academy of Science (UCAS), Beijing, China; 24 Institute of Physics, University of Belgrade, Belgrade, Serbia; 25 Department for Physics and Technology, University of Bergen, Bergen, Norway; 26 Physics Division, Lawrence Berkeley National Laboratory and University of California, Berkeley, CA USA; 27 Institut für Physik, Humboldt Universität zu Berlin, Berlin, Germany; 28 Albert Einstein Center for Fundamental Physics and Laboratory for High Energy Physics, University of Bern, Bern, Switzerland; 29 School of Physics and Astronomy, University of Birmingham, Birmingham, UK; 30 Centro de Investigaciónes, Universidad Antonio Nariño, Bogotá, Colombia; 31 Dipartimento di Fisica e Astronomia, Università di Bologna, Bologna, Italy; 32 INFN Sezione di Bologna, Bologna, Italy; 33 Physikalisches Institut, Universität Bonn, Bonn, Germany; 34 Department of Physics, Boston University, Boston, MA USA; 35 Department of Physics, Brandeis University, Waltham, MA USA; 36 Transilvania University of Brasov, Brasov, Romania; 37 Horia Hulubei National Institute of Physics and Nuclear Engineering, Bucharest, Romania; 38 Department of Physics, Alexandru Ioan Cuza University of Iasi, Iasi, Romania; 39 Physics Department, National Institute for Research and Development of Isotopic and Molecular Technologies, Cluj-Napoca, Romania; 40 University Politehnica Bucharest, Bucharest, Romania; 41 West University in Timisoara, Timisoara, Romania; 42 Faculty of Mathematics, Physics and Informatics, Comenius University, Bratislava, Slovak Republic; 43 Department of Subnuclear Physics, Institute of Experimental Physics of the Slovak Academy of Sciences, Kosice, Slovak Republic; 44 Physics Department, Brookhaven National Laboratory, Upton, NY USA; 45 Departamento de Física, Universidad de Buenos Aires, Buenos Aires, Argentina; 46 Cavendish Laboratory, University of Cambridge, Cambridge, UK; 47 Department of Physics, University of Cape Town, Cape Town, South Africa; 48 Department of Mechanical Engineering Science, University of Johannesburg, Johannesburg, South Africa; 49 School of Physics, University of the Witwatersrand, Johannesburg, South Africa; 50 Department of Physics, Carleton University, Ottawa, ON Canada; 51 Faculté des Sciences Ain Chock, Réseau Universitaire de Physique des Hautes Energies-Université Hassan II, Casablanca, Morocco; 52 Centre National de l’Energie des Sciences Techniques Nucleaires (CNESTEN), Rabat, Morocco; 53 Faculté des Sciences Semlalia, Université Cadi Ayyad, LPHEA, Marrakech, Morocco; 54 Faculté des Sciences, Université Mohamed Premier and LPTPM, Oujda, Morocco; 55 Faculté des sciences, Université Mohammed V, Rabat, Morocco; 56 CERN, Geneva, Switzerland; 57 Enrico Fermi Institute, University of Chicago, Chicago, IL USA; 58 LPC, Université Clermont Auvergne, CNRS/IN2P3, Clermont-Ferrand, France; 59 Nevis Laboratory, Columbia University, Irvington, NY USA; 60 Niels Bohr Institute, University of Copenhagen, Copenhagen, Denmark; 61 Dipartimento di Fisica, Università della Calabria, Rende, Italy; 62 INFN Gruppo Collegato di Cosenza, Laboratori Nazionali di Frascati, Frascati, Italy; 63 Physics Department, Southern Methodist University, Dallas, TX USA; 64 Physics Department, University of Texas at Dallas, Richardson, TX USA; 65 Department of Physics, Stockholm University, Stockholm, Sweden; 66 Oskar Klein Centre, Stockholm, Sweden; 67 Deutsches Elektronen-Synchrotron DESY, Hamburg and Zeuthen, Germany; 68 Lehrstuhl für Experimentelle Physik IV, Technische Universität Dortmund, Dortmund, Germany; 69 Institut für Kern- und Teilchenphysik, Technische Universität Dresden, Dresden, Germany; 70 Department of Physics, Duke University, Durham, NC USA; 71 SUPA-School of Physics and Astronomy, University of Edinburgh, Edinburgh, UK; 72 INFN e Laboratori Nazionali di Frascati, Frascati, Italy; 73 Physikalisches Institut, Albert-Ludwigs-Universität Freiburg, Freiburg, Germany; 74 II Physikalisches Institut, Georg-August-Universität Göttingen, Göttingen, Germany; 75 Département de Physique Nucléaire et Corpusculaire, Université de Genève, Geneva, Switzerland; 76 Dipartimento di Fisica, Università di Genova, Genoa, Italy; 77 INFN Sezione di Genova, Genoa, Italy; 78 II. Physikalisches Institut, Justus-Liebig-Universität Giessen, Giessen, Germany; 79 SUPA-School of Physics and Astronomy, University of Glasgow, Glasgow, UK; 80 LPSC, Université Grenoble Alpes, CNRS/IN2P3, Grenoble INP, Grenoble, France; 81 Laboratory for Particle Physics and Cosmology, Harvard University, Cambridge, MA USA; 82 Department of Modern Physics and State Key Laboratory of Particle Detection and Electronics, University of Science and Technology of China, Hefei, China; 83 Institute of Frontier and Interdisciplinary Science and Key Laboratory of Particle Physics and Particle Irradiation (MOE), Shandong University, Qingdao, China; 84 School of Physics and Astronomy, Shanghai Jiao Tong University, KLPPAC-MoE, SKLPPC, Shanghai, China; 85 Tsung-Dao Lee Institute, Shanghai, China; 86 Kirchhoff-Institut für Physik, Ruprecht-Karls-Universität Heidelberg, Heidelberg, Germany; 87 Physikalisches Institut, Ruprecht-Karls-Universität Heidelberg, Heidelberg, Germany; 88 Faculty of Applied Information Science, Hiroshima Institute of Technology, Hiroshima, Japan; 89 Department of Physics, Chinese University of Hong Kong, Shatin, N.T., Hong Kong, China; 90 Department of Physics, University of Hong Kong, Hong Kong, China; 91 Department of Physics and Institute for Advanced Study, Hong Kong University of Science and Technology, Clear Water Bay, Kowloon, Hong Kong, China; 92 Department of Physics, National Tsing Hua University, Hsinchu, Taiwan; 93 Department of Physics, Indiana University, Bloomington, IN USA; 94 INFN Gruppo Collegato di Udine, Sezione di Trieste, Udine, Italy; 95 ICTP, Trieste, Italy; 96 Dipartimento di Chimica, Fisica e Ambiente, Università di Udine, Udine, Italy; 97 INFN Sezione di Lecce, Lecce, Italy; 98 Dipartimento di Matematica e Fisica, Università del Salento, Lecce, Italy; 99 INFN Sezione di Milano, Milan, Italy; 100 Dipartimento di Fisica, Università di Milano, Milan, Italy; 101 INFN Sezione di Napoli, Naples, Italy; 102 Dipartimento di Fisica, Università di Napoli, Naples, Italy; 103 INFN Sezione di Pavia, Pavia, Italy; 104 Dipartimento di Fisica, Università di Pavia, Pavia, Italy; 105 INFN Sezione di Pisa, Pisa, Italy; 106 Dipartimento di Fisica E. Fermi, Università di Pisa, Pisa, Italy; 107 INFN Sezione di Roma, Rome, Italy; 108 Dipartimento di Fisica, Sapienza Università di Roma, Rome, Italy; 109 INFN Sezione di Roma Tor Vergata, Rome, Italy; 110 Dipartimento di Fisica, Università di Roma Tor Vergata, Rome, Italy; 111 INFN Sezione di Roma Tre, Rome, Italy; 112 Dipartimento di Matematica e Fisica, Università Roma Tre, Rome, Italy; 113 INFN-TIFPA, Trento, Italy; 114 Università degli Studi di Trento, Trento, Italy; 115 Institut für Astro- und Teilchenphysik, Leopold-Franzens-Universität, Innsbruck, Austria; 116 University of Iowa, Iowa City, IA USA; 117 Department of Physics and Astronomy, Iowa State University, Ames, IA USA; 118 Joint Institute for Nuclear Research, Dubna, Russia; 119 Departamento de Engenharia Elétrica, Universidade Federal de Juiz de Fora (UFJF), Juiz de Fora, Brazil; 120 Universidade Federal do Rio De Janeiro COPPE/EE/IF, Rio de Janeiro, Brazil; 121 Universidade Federal de São João del Rei (UFSJ), São João del Rei, Brazil; 122 Instituto de Física, Universidade de São Paulo, São Paulo, Brazil; 123 KEK, High Energy Accelerator Research Organization, Tsukuba, Japan; 124 Graduate School of Science, Kobe University, Kobe, Japan; 125 Faculty of Physics and Applied Computer Science, AGH University of Science and Technology, Kraków, Poland; 126 Marian Smoluchowski Institute of Physics, Jagiellonian University, Kraków, Poland; 127 Institute of Nuclear Physics Polish Academy of Sciences, Kraków, Poland; 128 Faculty of Science, Kyoto University, Kyoto, Japan; 129 Kyoto University of Education, Kyoto, Japan; 130 Research Center for Advanced Particle Physics and Department of Physics, Kyushu University, Fukuoka, Japan; 131 Instituto de Física La Plata, Universidad Nacional de La Plata and CONICET, La Plata, Argentina; 132 Physics Department, Lancaster University, Lancaster, UK; 133 Oliver Lodge Laboratory, University of Liverpool, Liverpool, UK; 134 Department of Experimental Particle Physics, Jožef Stefan Institute and Department of Physics, University of Ljubljana, Ljubljana, Slovenia; 135 School of Physics and Astronomy, Queen Mary University of London, London, UK; 136 Department of Physics, Royal Holloway University of London, Egham, UK; 137 Department of Physics and Astronomy, University College London, London, UK; 138 Louisiana Tech University, Ruston, LA USA; 139 Fysiska institutionen, Lunds universitet, Lund, Sweden; 140 Centre de Calcul de l’Institut National de Physique Nucléaire et de Physique des Particules (IN2P3), Villeurbanne, France; 141 Departamento de Física Teorica C-15 and CIAFF, Universidad Autónoma de Madrid, Madrid, Spain; 142 Institut für Physik, Universität Mainz, Mainz, Germany; 143 School of Physics and Astronomy, University of Manchester, Manchester, UK; 144 CPPM, Aix-Marseille Université, CNRS/IN2P3, Marseille, France; 145 Department of Physics, University of Massachusetts, Amherst, MA USA; 146 Department of Physics, McGill University, Montreal, QC Canada; 147 School of Physics, University of Melbourne, Melbourne, VIC Australia; 148 Department of Physics, University of Michigan, Ann Arbor, MI USA; 149 Department of Physics and Astronomy, Michigan State University, East Lansing, MI USA; 150 B.I. Stepanov Institute of Physics, National Academy of Sciences of Belarus, Minsk, Belarus; 151 Research Institute for Nuclear Problems of Byelorussian State University, Minsk, Belarus; 152 Group of Particle Physics, University of Montreal, Montreal, QC Canada; 153 P.N. Lebedev Physical Institute of the Russian Academy of Sciences, Moscow, Russia; 154 Institute for Theoretical and Experimental Physics (ITEP), Moscow, Russia; 155 National Research Nuclear University MEPhI, Moscow, Russia; 156 D.V. Skobeltsyn Institute of Nuclear Physics, M.V. Lomonosov Moscow State University, Moscow, Russia; 157 Fakultät für Physik, Ludwig-Maximilians-Universität München, Munich, Germany; 158 Max-Planck-Institut für Physik (Werner-Heisenberg-Institut), Munich, Germany; 159 Nagasaki Institute of Applied Science, Nagasaki, Japan; 160 Graduate School of Science and Kobayashi-Maskawa Institute, Nagoya University, Nagoya, Japan; 161 Department of Physics and Astronomy, University of New Mexico, Albuquerque, NM USA; 162 Institute for Mathematics, Astrophysics and Particle Physics, Radboud University Nijmegen/Nikhef, Nijmegen, The Netherlands; 163 Nikhef National Institute for Subatomic Physics, University of Amsterdam, Amsterdam, The Netherlands; 164 Department of Physics, Northern Illinois University, DeKalb, IL USA; 165 Budker Institute of Nuclear Physics, SB RAS, Novosibirsk, Russia; 166 Novosibirsk State University, Novosibirsk, Russia; 167 Institute for High Energy Physics of the National Research Centre Kurchatov Institute, Protvino, Russia; 168 Department of Physics, New York University, New York, NY USA; 169 Ohio State University, Columbus, OH USA; 170 Faculty of Science, Okayama University, Okayama, Japan; 171 Homer L. Dodge Department of Physics and Astronomy, University of Oklahoma, Norman, OK USA; 172 Department of Physics, Oklahoma State University, Stillwater, OK USA; 173 RCPTM, Joint Laboratory of Optics, Palacký University, Olomouc, Czech Republic; 174 Center for High Energy Physics, University of Oregon, Eugene, OR USA; 175 LAL, Université Paris-Sud, CNRS/IN2P3, Université Paris-Saclay, Orsay, France; 176 Graduate School of Science, Osaka University, Osaka, Japan; 177 Department of Physics, University of Oslo, Oslo, Norway; 178 Department of Physics, Oxford University, Oxford, UK; 179 LPNHE, Sorbonne Université, Paris Diderot Sorbonne Paris Cité, CNRS/IN2P3, Paris, France; 180 Department of Physics, University of Pennsylvania, Philadelphia, PA USA; 181 Konstantinov Nuclear Physics Institute of National Research Centre “Kurchatov Institute”, PNPI, St. Petersburg, Russia; 182 Department of Physics and Astronomy, University of Pittsburgh, Pittsburgh, PA USA; 183 Laboratório de Instrumentação e Física Experimental de Partículas-LIP, Lisbon, Portugal; 184 Departamento de Física, Faculdade de Ciências, Universidade de Lisboa, Lisbon, Portugal; 185 Departamento de Física, Universidade de Coimbra, Coimbra, Portugal; 186 Centro de Física Nuclear da Universidade de Lisboa, Lisbon, Portugal; 187 Departamento de Física, Universidade do Minho, Braga, Portugal; 188 Departamento de Física Teorica y del Cosmos, Universidad de Granada, Granada, Spain; 189 Dep Física and CEFITEC of Faculdade de Ciências e Tecnologia, Universidade Nova de Lisboa, Caparica, Portugal; 190 Institute of Physics, Academy of Sciences of the Czech Republic, Prague, Czech Republic; 191 Czech Technical University in Prague, Prague, Czech Republic; 192 Faculty of Mathematics and Physics, Charles University, Prague, Czech Republic; 193 Particle Physics Department, Rutherford Appleton Laboratory, Didcot, UK; 194 IRFU, CEA, Université Paris-Saclay, Gif-sur-Yvette, France; 195 Santa Cruz Institute for Particle Physics, University of California Santa Cruz, Santa Cruz, CA USA; 196 Departamento de Física, Pontificia Universidad Católica de Chile, Santiago, Chile; 197 Departamento de Física, Universidad Técnica Federico Santa María, Valparaiso, Chile; 198 Department of Physics, University of Washington, Seattle, WA USA; 199 Department of Physics and Astronomy, University of Sheffield, Sheffield, UK; 200 Department of Physics, Shinshu University, Nagano, Japan; 201 Department Physik, Universität Siegen, Siegen, Germany; 202 Department of Physics, Simon Fraser University, Burnaby, BC Canada; 203 SLAC National Accelerator Laboratory, Stanford, CA USA; 204 Physics Department, Royal Institute of Technology, Stockholm, Sweden; 205 Departments of Physics and Astronomy, Stony Brook University, Stony Brook, NY USA; 206 Department of Physics and Astronomy, University of Sussex, Brighton, UK; 207 School of Physics, University of Sydney, Sydney, Australia; 208 Institute of Physics, Academia Sinica, Taipei, Taiwan; 209 Academia Sinica Grid Computing, Institute of Physics, Academia Sinica, Taipei, Taiwan; 210 E. Andronikashvili Institute of Physics, Iv. Javakhishvili Tbilisi State University, Tbilisi, Georgia; 211 High Energy Physics Institute, Tbilisi State University, Tbilisi, Georgia; 212 Department of Physics, Technion: Israel Institute of Technology, Haifa, Israel; 213 Raymond and Beverly Sackler School of Physics and Astronomy, Tel Aviv University, Tel Aviv, Israel; 214 Department of Physics, Aristotle University of Thessaloniki, Thessaloniki, Greece; 215 International Center for Elementary Particle Physics and Department of Physics, University of Tokyo, Tokyo, Japan; 216 Graduate School of Science and Technology, Tokyo Metropolitan University, Tokyo, Japan; 217 Department of Physics, Tokyo Institute of Technology, Tokyo, Japan; 218 Tomsk State University, Tomsk, Russia; 219 Department of Physics, University of Toronto, Toronto, ON Canada; 220 TRIUMF, Vancouver, BC Canada; 221 Department of Physics and Astronomy, York University, Toronto, ON Canada; 222 Division of Physics and Tomonaga Center for the History of the Universe, Faculty of Pure and Applied Sciences, University of Tsukuba, Tsukuba, Japan; 223 Department of Physics and Astronomy, Tufts University, Medford, MA USA; 224 Department of Physics and Astronomy, University of California Irvine, Irvine, CA USA; 225 Department of Physics and Astronomy, University of Uppsala, Uppsala, Sweden; 226 Department of Physics, University of Illinois, Urbana, IL USA; 227 Instituto de Física Corpuscular (IFIC), Centro Mixto Universidad de Valencia-CSIC, Valencia, Spain; 228 Department of Physics, University of British Columbia, Vancouver, BC Canada; 229 Department of Physics and Astronomy, University of Victoria, Victoria, BC Canada; 230 Fakultät für Physik und Astronomie, Julius-Maximilians-Universität Würzburg, Würzburg, Germany; 231 Department of Physics, University of Warwick, Coventry, UK; 232 Waseda University, Tokyo, Japan; 233 Department of Particle Physics, Weizmann Institute of Science, Rehovot, Israel; 234 Department of Physics, University of Wisconsin, Madison, WI USA; 235 Fakultät für Mathematik und Naturwissenschaften, Fachgruppe Physik, Bergische Universität Wuppertal, Wuppertal, Germany; 236 Department of Physics, Yale University, New Haven, CT USA; 237 Yerevan Physics Institute, Yerevan, Armenia; 238 CERN, 1211 Geneva 23, Switzerland

## Abstract

The performance of the missing transverse momentum ($E_{\mathrm{T}}^{\mathrm{miss}}$) reconstruction with the ATLAS detector is evaluated using data collected in proton–proton collisions at the LHC at a centre-of-mass energy of 13 TeV in 2015. To reconstruct $E_{\mathrm{T}}^{\mathrm{miss}}$, fully calibrated electrons, muons, photons, hadronically decaying $\tau \text {-leptons}$, and jets reconstructed from calorimeter energy deposits and charged-particle tracks are used. These are combined with the soft hadronic activity measured by reconstructed charged-particle tracks not associated with the hard objects. Possible double counting of contributions from reconstructed charged-particle tracks from the inner detector, energy deposits in the calorimeter, and reconstructed muons from the muon spectrometer is avoided by applying a signal ambiguity resolution procedure which rejects already used signals when combining the various $E_{\mathrm{T}}^{\mathrm{miss}}$ contributions. The individual terms as well as the overall reconstructed $E_{\mathrm{T}}^{\mathrm{miss}}$ are evaluated with various performance metrics for scale (linearity), resolution, and sensitivity to the data-taking conditions. The method developed to determine the systematic uncertainties of the $E_{\mathrm{T}}^{\mathrm{miss}}$ scale and resolution is discussed. Results are shown based on the full 2015 data sample corresponding to an integrated luminosity of $3.2~\hbox {fb}^{-1}$.

## Introduction

The missing transverse momentum ($E_{\mathrm{T}}^{\mathrm{miss}}$) is an important observable serving as an experimental proxy for the transverse momentum carried by undetected particles produced in proton–proton ($pp$) collisions measured with the ATLAS detector [1] at the Large Hadron Collider (LHC). It is reconstructed from the signals of detected particles in the final state. A value incompatible with zero may indicate not only the production of Standard Model (SM) neutrinos but also the production of new particles suggested in models for physics beyond the SM that escape the ATLAS detector without being detected. The reconstruction of $E_{\mathrm{T}}^{\mathrm{miss}}$ is challenging because it involves all detector subsystems and requires the most complete and unambiguous representation of the hard interaction of interest by calorimeter and tracking signals. This representation is obscured by limitations introduced by the detector acceptance and by signals and signal remnants from additional $pp$ interactions occurring in the same, previous and subsequent LHC bunch crossings (pile-up) relative to the triggered hard-scattering. ATLAS has developed successful strategies for a high-quality $E_{\mathrm{T}}^{\mathrm{miss}}$ reconstruction focussing on the minimisation of effects introduced by pile-up for the data recorded between 2010 and 2012 (LHC $\text {Run}\,1$) [2, 3]. These approaches are the basis for the $E_{\mathrm{T}}^{\mathrm{miss}}$ reconstruction developed for the data collected in 2015 (LHC $\text {Run}\,2$) that is described in this paper, together with results from performance evaluations and the determination of systematic uncertainties.

This paper is organised as follows. The subsystems forming the ATLAS detector are described in Sect. 2. The $E_{\mathrm{T}}^{\mathrm{miss}}$ reconstruction is discussed in Sect. 3. The extraction of the data samples and the generation of the Monte Carlo (MC) simulation samples are presented in Sect. 4. The event selection is outlined in Sect. 5, followed by results for $E_{\mathrm{T}}^{\mathrm{miss}}$ performance in Sect. 6. Section 7 comprises a discussion of methods used to determine systematic uncertainties associated with the $E_{\mathrm{T}}^{\mathrm{miss}}$ measurement, and the presentation of the corresponding results. Section 8 describes variations of the $E_{\mathrm{T}}^{\mathrm{miss}}$ reconstruction using calorimeter signals for the soft hadronic event activity, or reconstructed charged-particle tracks only. The paper concludes with a summary and outlook in Sect. 9. The nomenclature and conventions used by ATLAS for $E_{\mathrm{T}}^{\mathrm{miss}}$-related variables and descriptors can be found in Appendix A, while the composition of $E_{\mathrm{T}}^{\mathrm{miss}}$ reconstruction variants is presented in Appendix B. An evaluation of the effect of alternative jet selections on the $E_{\mathrm{T}}^{\mathrm{miss}}$ reconstruction performance is given in Appendix C.

## ATLAS detector

The ATLAS experiment at the LHC features a multi-purpose particle detector with a forward–backward symmetric cylindrical geometry and a nearly full ($4\pi$) coverage in solid angle.^1^ It consists of an inner detector (ID) tracking system in a $2\,\text {T}$ axial magnetic field provided by a superconducting solenoid. The solenoid is surrounded by electromagnetic and hadronic calorimeters, and a muon spectrometer (MS). The ID covers the pseudorapidity range $|\eta | < 2.5$, and consists of a silicon pixel detector, a silicon microstrip detector and a transition radiation tracker for $|\eta | < 2.0$. During the LHC shutdown between $\text {Run}\,1$ and $\text {Run}\,2$, a new tracking layer, known as the insertable B-layer [4], was added between the previous innermost pixel layer and a new, narrower beam pipe.

The high-granularity lead/liquid-argon (LAr) sampling electromagnetic calorimeter covers the region $|\eta | < 3.2$. The regions $|\eta | < 1.37$ and $1.5< |\eta | < 1.8$ are instrumented with presamplers in front of the LAr calorimeter in the same cryostat. A steel/scintillator-tile calorimeter (Tile) provides hadronic coverage in the central pseudorapidity range $|\eta | < 1.7$. LAr technology is also used for the hadronic calorimeters in the endcap region $1.5< |\eta | < 3.2$ and for electromagnetic and hadronic energy measurements in the forward calorimeters covering $3.2< |\eta | < 4.9$.

The MS surrounds the calorimeters. It consists of three large superconducting air-core toroidal magnets, precision tracking chambers providing precise muon tracking out to $|\eta | = 2.7$, and fast detectors for triggering in the region $|\eta | < 2.4$.

A two-level trigger system is used to select events [5]. A low-level hardware trigger reduces the data rate, and a high-level software trigger selects events with interesting final states. More details of the ATLAS detector can be found in Ref. [1].

## $E_{\mathrm{T}}^{\mathrm{miss}}$ reconstruction

The reconstructed $E_{\mathrm{T}}^{\mathrm{miss}}$ in ATLAS is characterised by two contributions. The first one is from the *hard-event* signals comprising fully reconstructed and calibrated particles and jets (hard objects). The reconstructed particles are electrons, photons, $\tau \text {-leptons}$, and muons. While muons are reconstructed from ID and MS tracks, electrons and $\tau \text {-leptons}$ are identified combining calorimeter signals with tracking information. Photons and jets are principally reconstructed from calorimeter signals, with possible signal refinements from reconstructed tracks. The second contribution to $E_{\mathrm{T}}^{\mathrm{miss}}$ is from the *soft-event* signals consisting of reconstructed charged-particle tracks (soft signals) associated with the hard-scatter vertex defined in Appendix A but not with the hard objects.

ATLAS carries out a dedicated reconstruction procedure for each kind of particle as well as for jets, casting a particle or jet hypothesis on the origin of (a group of) detector signals. These procedures are independent of one another. This means that e.g. the same calorimeter signal used to reconstruct an electron is likely also used to reconstruct a jet, thus potentially introducing double counting of the same signal when reconstructing $E_{\mathrm{T}}^{\mathrm{miss}}$. This issue is addressed by the explicit *signal ambiguity resolution* in the object-based $E_{\mathrm{T}}^{\mathrm{miss}}$ reconstruction originally introduced in Refs. [2, 3], and by its 2015 implementation described in Sects. 3.1 and 3.2.

Additional options for the set of signals used to reconstruct $E_{\mathrm{T}}^{\mathrm{miss}}$ are available and discussed in detail in Sect. 8. One of these alternative options is the calorimeter-based $E_{\mathrm{T}}^{\mathrm{miss}}$ reconstruction discussed in Sect. 8.1, which uses a soft event built from clusters of topologically connected calorimeter cells (topo-clusters) [6]. Another option is the track-based missing transverse momentum, which differs from $E_{\mathrm{T}}^{\mathrm{miss}}$ only in the use of tracks in place of jets. It is described in more detail in Sect. 8.2.

### $E_{\mathrm{T}}^{\mathrm{miss}}$ basics

The missing transverse momentum reconstruction provides a set of observables constructed from the components $p_{x(y)}$ of the transverse momentum vectors ($\mathbf p _{\mathrm{T}}$) of the various contributions. The missing transverse momentum components $E_{x(y)}^{{\text {miss}}}$ serve as the basic input for most of these observables. They are given by

1 $$\begin{aligned} E_{x(y)}^{{\text {miss}}} = - \sum _{i\in \{\text {hard objects}\}} p_{x(y),i} - \sum _{j\in \{\text {soft signals}\}} p_{x(y),j} . \end{aligned}$$

The set of observables constructed from $E_{x(y)}^{{\text {miss}}}$ is

2 $$\begin{aligned} \mathbf E _{\mathrm{T}}^{{\text {miss}}}= & {} (E_{x}^{{\text {miss}}},E_{y}^{{\text {miss}}}), \end{aligned}$$

3 $$\begin{aligned} E_{\mathrm{T}}^{\mathrm{miss}}= & {} |\mathbf E _{\mathrm{T}}^{{\text {miss}}} | = \sqrt{(E_{x}^{{\text {miss}}})^{2}+(E_{y}^{{\text {miss}}})^{2}}, \end{aligned}$$

4 $$\begin{aligned} \phi ^{{\text {miss}}}= & {} \tan ^{-1}(E_{y}^{{\text {miss}}}/E_{x}^{{\text {miss}}}). \end{aligned}$$

The vector $\mathbf E _{\mathrm{T}}^{{\text {miss}}}$ provides the amount of the missing transverse momentum via its magnitude $E_{\mathrm{T}}^{\mathrm{miss}}$, and its direction in the transverse plane in terms of the azimuthal angle $\phi ^{{\text {miss}}}$. Consequently, $E_{\mathrm{T}}^{\mathrm{miss}}$ is non-negative by definition. However, in an experimental environment where not all relevant $p_{\mathrm{T}}$ from the hard-scatter interaction can be reconstructed and used in Eq. (1), and the reconstructed $p_{\mathrm{T}}$ from each contribution is affected by the limited resolution of the detector, an *observation bias* towards non-vanishing values for $E_{\mathrm{T}}^{\mathrm{miss}}$ is introduced even for final states without genuine missing transverse momentum generated by undetectable particles.

The scalar sum of all transverse momenta ($p_{\mathrm{T}} = |\mathbf p _{\mathrm{T}} |$) from the objects contributing to $E_{\mathrm{T}}^{\mathrm{miss}}$ reconstruction is given by

5 $$\begin{aligned} \Sigma E_{\mathrm{T}} = \sum _{i\in \{\text {hard objects}\}} p_{\mathrm{T},i} + \sum _{j\in \{\text {soft signals}\}} p_{\mathrm{T},j}. \end{aligned}$$

In the context of $E_{\mathrm{T}}^{\mathrm{miss}}$ reconstruction, $\Sigma E_{\mathrm{T}}$ is calculated in addition to the sum given in Eq. (1), and the derived quantities defining $E_{\mathrm{T}}^{\mathrm{miss}}$ given in Eqs. (2)–(4). It provides a useful overall scale for evaluating the hardness of the hard-scatter event in the transverse plane, and thus provides a measure for the event activity in physics analyses and $E_{\mathrm{T}}^{\mathrm{miss}}$ reconstruction performance studies.

In the calculation of $E_{x(y)}^{{\text {miss}}}$ and $\Sigma E_{\mathrm{T}}$ the contributing objects need to be reconstructed from mutually exclusive detector signals. This rule avoids multiple inclusions of the same signal in all constructed observables. The implementation of this rule in terms of the signal ambiguity resolution requires the definition of a sequence for selected contributions, in addition to a rejection mechanism based on common signal usage between different objects. Similarly to the analysis presented in Ref. [3], the most commonly used order for the $E_{\mathrm{T}}^{\mathrm{miss}}$ reconstruction sequence for the hard-object contribution starts with electrons ($e$), followed by photons ($\gamma$), then hadronically decaying $\tau \text {-leptons}$ ($\tau _{\mathrm{had}}$), and finally jets. Muons ($\mu$) are principally reconstructed from ID and MS tracks alone, with corrections based on their energy loss in the calorimeter, leading to little or no signal overlap with the other reconstructed particles in the calorimeter.

In the sequence discussed here, all electrons passing the selection enter the $E_{\mathrm{T}}^{\mathrm{miss}}$ reconstruction first. The lower-priority reconstructed particles ($\gamma$, $\tau _{\mathrm{had}}$) are fully rejected if they share their calorimeter signal with a higher-priority object that has already entered the $E_{\mathrm{T}}^{\mathrm{miss}}$ reconstruction. Muons experience energy loss in the calorimeters, but only non-isolated muons overlap with other hard objects, most likely jets or $\tau \text {-leptons}$. In this case the muon’s energy deposit in the calorimeter cannot be separated from the overlapping jet-like objects with the required precision, and the calorimeter-signal-overlap resolution based on the shared use of topo-clusters cannot be applied. A discussion of the treatment of isolated and non-isolated muons is given in Sect. 3.3.4.

Generally, jets are rejected if they overlap with accepted higher-priority particles. To avoid signal losses for $E_{\mathrm{T}}^{\mathrm{miss}}$ reconstruction in the case of partial or marginal overlap, and to suppress the accidental inclusion of jets reconstructed from calorimeter signals from large muon energy losses or pile-up, the more refined overlap resolution strategies described in Sects. 3.3.5 and 3.3.6 are applied.

Excluding ID tracks associated with any of the accepted hard objects contributing to $E_{\mathrm{T}}^{\mathrm{miss}}$, ID tracks from the hard-scatter collision vertex are used to construct the soft-event signal for the results presented in this paper.

**Table 1:** Overview of the contributions to $E_{\mathrm{T}}^{\mathrm{miss}}$ and $\Sigma E_{\mathrm{T}}$ from hard objects such as electrons ($e$), photons ($\gamma$), hadronically decaying $\tau \text {-leptons}$ ($\tau _{\mathrm{had}}$), muons ($\mu$), and jets, together with the signals for the soft term. The configuration shown is the one used as reference for the performance evaluations presented in this paper. The table is ordered descending in priority $\mathbb {P}$ of consideration for $E_{\mathrm{T}}^{\mathrm{miss}}$ reconstruction, with (1) being the first and (5) being the last calculated hard-object contribution. The soft-event contribution is constructed at the lowest priority (6), after all hard objects are considered. The transverse (longitudinal) impact parameter $d_{0}$ ($z_{0} \sin (\theta )$) used to select the ID tracks contributing to $E_{\mathrm{T}}^{{\text {miss}},{\text {soft}}}$ and $\Sigma E_{\mathrm{T}}^{{\text {soft}}}$ in $\mathbb {P} = (6)$ is measured relative to the hard-scatter vertex. All variables are explained in Sect. 3.2. The angular distance $\Delta R$ between objects is defined as $\Delta R = \sqrt{(\Delta \eta )^{2}+(\Delta \phi )^{2}}$

$\mathbb {P}$	Objects contributing to $E_{\mathrm{T}}^{\mathrm{miss}}$ and $\Sigma E_{\mathrm{T}}$			
	Type	Selections	Variables	Comments
(1)	$e$	${|\eta |< 1.37}\text { or }{1.52< |\eta | < 2.47}$	$E_{\mathrm{T}}^{{\text {miss}},e}$	All $e^{\pm }$ passing medium reconstruction quality and kinematic selections
		${p_{\mathrm{T}} > 10\,{\text {Ge}\text {V}}}$	$\Sigma E_{\mathrm{T}}^{e}$	
(2)	$\gamma$	${{|\eta |< 1.37}\text { or }{1.52< |\eta | < 2.47}}$	$E_{\mathrm{T}}^{{\text {miss}},\gamma }$	All $\gamma$ passing tight quality and kinematic selections in reconstruction, and without signal overlap with (1)
		${p_{\mathrm{T}} > 25\,{\text {Ge}\text {V}}}$	$\Sigma E_{\mathrm{T}}^{\gamma }$	
(3)	$\tau _{\mathrm{had}}$	${|\eta |< 1.37}\text { or }{1.52< |\eta | < 2.47}$	$E_{\mathrm{T}}^{{\text {miss}},\tau _{\mathrm{had}}}$	All $\tau _{\mathrm{had}}$ passing medium reconstruction quality and kinematic selections, and without signal overlap with (1) and (2)
		${p_{\mathrm{T}} > 20\,{\text {Ge}\text {V}}}$	$\Sigma E_{\mathrm{T}}^{\tau _{\mathrm{had}}}$	
(4)	$\mu$	${|\eta | < 2.7}$	$E_{\mathrm{T}}^{{\text {miss}},\mu }$	All $\mu$ passing medium quality and kinematic selections in reconstruction; for the discussion of the $\mu$–jet overlap removal see Sect. 3.3.6
		${p_{\mathrm{T}} > 10\,{\text {Ge}\text {V}}}$	$\Sigma E_{\mathrm{T}}^{\mu }$	
(5)	Jet	$|\eta | < 4.5$	$E_{\mathrm{T}}^{{\text {miss}},\text {jet}}$	All jets passing reconstruction quality (jet cleaning) and kinematic selections, and without signal $\hbox {overlap}^{\mathrm{a}}$ with (1)–(3); for the dedicated overlap removal strategy with $\mu$ from (4) see Sect. 3.3.6
		${p_{\mathrm{T}} > 60\,{\text {Ge}\text {V}}}$	$\Sigma E_{\mathrm{T}}^{\text {jet}}$	

		$$2.4< |\eta | < 4.5$$		
		$${20\,{\text {Ge}\text {V}}< p_{\mathrm{T}} < 60\,{\text {Ge}\text {V}}}$$		

		$$|\eta | < 2.4$$		
		$$20\,{\text {Ge}\text {V}}< p_{\mathrm{T}} < 60\,{\text {Ge}\text {V}} $$		
		$$\text {JVT} > 0.59$$		
(6)	ID track	$p_{\mathrm{T}} > 400\,{\text {Me}\text {V}}$	$E_{\mathrm{T}}^{{\text {miss}},{\text {soft}}}$	All ID tracks from the hard-scatter vertex passing reconstruction quality and kinematic selections, and not associated with any particle from (1), (3) or (4), or ghost-associated with a jet from (5)
		$|d_{0}| < 1.5\,{\text {mm}}$	$\Sigma E_{\mathrm{T}}^{{\text {soft}}}$	
		$$|z_{0}\sin (\theta )| < 1.5\,{\text {mm}} $$		
		$$\Delta R(\text {track},e-/\gamma \text {cluster}) > 0.05$$		
		$$\Delta R(\text {track},\tau _{\mathrm{had}}) > 0.2$$		

$^\mathrm{a}$While for single reconstructed particles no overlap is accepted at all, jets with a signal overlap fraction $\kappa _{E} < 50$% can still contribute their associated tracks to $E_{\mathrm{T}}^{{\text {miss}},{\text {soft}}}$ if those pass the selections for $\mathbb {P} = (6)$, as discussed in Sect. 3.3.5. The definition of $\kappa _{E}$ is given in Eq. (8)

### $E_{\mathrm{T}}^{\mathrm{miss}}$ terms

Particle and jet selections in a given analysis should be reflected in $E_{\mathrm{T}}^{\mathrm{miss}}$ and $\Sigma E_{\mathrm{T}}$ for a consistent interpretation of a given event. Each reconstructed particle and jet has its own dedicated calibration translating the detector signals into a fully corrected four-momentum. This means that e.g. rejecting certain electrons in a given analysis can change both $E_{\mathrm{T}}^{\mathrm{miss}}$ and $\Sigma E_{\mathrm{T}}$, if the corresponding calorimeter signal is included and calibrated as a jet or a significant part of a jet. This also means that systematic uncertainties for the different particles can be consistently propagated to $E_{\mathrm{T}}^{\mathrm{miss}}$. The applied selections are presented in Sect. 3.3, and summarised in Table 1.

In ATLAS the flexibility needed to recalculate $E_{\mathrm{T}}^{\mathrm{miss}}$ and $\Sigma E_{\mathrm{T}}$ under changing analysis requirements for the same event is implemented using dedicated variables corresponding to specific object contributions. In this approach the full $\mathbf E _{\mathrm{T}}^{{\text {miss}}}$ is the vectorial sum of missing transverse momentum terms $\mathbf E _{\mathrm{T}}^{{\text {miss}},p}$, with $p \in \{e,\gamma ,\tau _{\mathrm{had}},\mu ,\text {jet}\}$ reconstructed from the $\mathbf p _{\mathrm{T}} = (p_{x},p_{y})$ of accepted particles and jets, and the corresponding soft term $\mathbf E _{\mathrm{T}}^{{\text {miss}},\text {soft}}$ from the soft-event signals introduced in Sect. 3.1 and further specified in Sect. 3.4. This yields^2^
6The $E_{\mathrm{T}}^{\mathrm{miss}}$ and $\phi ^{{\text {miss}}}$ observables can be constructed according to Eqs. (3) and (4), respectively, for the overall missing transverse momentum (from $\mathbf E _{\mathrm{T}}^{{\text {miss}}}$) as well as for each individual term indicated in Eq. (6). In the priority-ordered reconstruction sequence for $E_{\mathrm{T}}^{\mathrm{miss}}$, contributions are defined by a combination of analysis-dependent selections and a possible rejection due to the applied signal ambiguity resolution. The muon and electron contributions are typically not subjected to the signal overlap resolution and are thus exclusively defined by the selection requirements. Unused tracks in Eq. (6) refers to those tracks associated with the hard-scatter vertex but not with any hard object. Neutral particle signals from the calorimeter suffer from significant contributions from pile-up and are not included in the soft term.

Correspondingly, $\Sigma E_{\mathrm{T}}$ is calculated from the scalar sums of the transverse momenta of hard objects entering the $E_{\mathrm{T}}^{\mathrm{miss}}$ reconstruction and the soft term,7The hard term in both $E_{\mathrm{T}}^{\mathrm{miss}}$ and $\Sigma E_{\mathrm{T}}$ is characterised by little dependence on pile-up, as it includes only fully calibrated objects, where the calibration includes a pile-up correction and objects tagged as originating from pile-up are removed. The particular choice of using only tracks from the hard-scatter vertex for the soft term strongly suppresses pile-up contributions to this term as well. The observed residual pile-up dependencies are discussed with the performance results shown in Sect. 6.

### Object selection

The following selections are applied to reconstructed particles and jets used for the performance evaluations presented in Sects. 6–8. Generally, these selections require refinements to achieve optimal $E_{\mathrm{T}}^{\mathrm{miss}}$ reconstruction performance in the context of a given physics analysis, and the selections performed in this study are an example set of criteria.

#### Electron selection

Reconstructed electrons are selected on the basis of their shower shapes in the calorimeter and how well their calorimeter cell clusters are matched to ID tracks [7]. Both are evaluated in a combined likelihood-based approach [8]. Electrons with at least medium reconstruction quality are selected. They are calibrated using the default calibration given in Ref. [7]. To be considered for $E_{\mathrm{T}}^{\mathrm{miss}}$ reconstruction, electrons passing the reconstruction quality requirements are in addition required to have $p_{\mathrm{T}} > 10\,{\text {Ge}\text {V}}$ and $|\eta | < 1.37$ or $1.52< |\eta | < 2.47$, to avoid the transition region between the central and endcap electromagnetic calorimeters. Any energy deposit by electrons within $1.37< |\eta | < 1.52$ is likely reconstructed as a jet and enters the $E_{\mathrm{T}}^{\mathrm{miss}}$ reconstruction as such, if this jet meets the corresponding selection criteria discussed in Sect. 3.3.5.

#### Photon selection

The identification and reconstruction of photons exploits the distinctive evolution of their electromagnetic showers in the calorimeters [9]. Photons are selected and calibrated using the tight selection criteria given in Ref. [7]. In addition to the reconstruction quality requirements, photons must have $p_{\mathrm{T}} > 25\,{\text {Ge}\text {V}}$ and $|\eta | < 1.37$ or $1.52< |\eta | < 2.37$ to be included in the $E_{\mathrm{T}}^{\mathrm{miss}}$ reconstruction. Similarly to electrons, photons emitted within $1.37< |\eta | < 1.52$ may contribute to $E_{\mathrm{T}}^{\mathrm{miss}}$ as a jet.

#### $\tau$-Lepton selection

Hadronically decaying $\tau \text {-leptons}$ are reconstructed from narrow jets with low associated track multiplicities [10]. Candidates must pass the medium quality selection given in Ref. [11], and in addition have $p_{\mathrm{T}} > 20\,{\text {Ge}\text {V}}$ and $|\eta | < 1.37$ or $1.52< |\eta | < 2.47$. Any $\tau \text {-lepton}$ not satisfying these $\tau$-identification criteria may contribute to $E_{\mathrm{T}}^{\mathrm{miss}}$ when passing the jet selection.

#### Muon selection

Muons are reconstructed within $|\eta | < 2.5$ employing a combined MS and ID track fit. Outside of the ID coverage, muons are reconstructed within $2.5< |\eta | < 2.7$ from a track fit to MS track segments alone. Muons are further selected for $E_{\mathrm{T}}^{\mathrm{miss}}$ reconstruction by requiring the medium reconstruction quality defined in Ref. [12], $p_{\mathrm{T}} > 10\,{\text {Ge}\text {V}}$, and an association with the hard-scatter vertex for those within $|\eta | < 2.5$.

#### Jet selection

Jets are reconstructed from clusters of topologically connected calorimeter cells (topo-clusters), described in Ref. [6]. The topo-clusters are calibrated at the electromagnetic (EM) energy scale.^3^ The anti-$k_{t}$ algorithm [13], as provided by the FastJet toolkit [14], is employed with a radius parameter $R = 0.4$ to form jets from these topo-clusters. The jets are fully calibrated using the EM+JES scheme [15] including a correction for pile-up [16]. They are required to have $p_{\mathrm{T}} > 20\,{\text {Ge}\text {V}}$ after the full calibration. The jet contribution to $E_{\mathrm{T}}^{\mathrm{miss}}$ and $\Sigma E_{\mathrm{T}}$ is primarily defined by the signal ambiguity resolution.

Jets not rejected at that stage are further filtered using a tagging algorithm to select hard-scatter jets (“jet vertex tagging”) [16]. This algorithm provides the jet vertex tagger variable JVT, ranging from 0 (pile-up-like) to 1 (hard-scatter-like), for each jet with matched tracks.^4^ The matching of tracks with jets is done by *ghost association*, where tracks are clustered as *ghost particles* into the jet, as described in Ref. [3] and based on the approach outlined in Ref. [17].

The overlap resolution can result in a partial overlap of the jet with an electron or photon, in terms of the fraction of common signals contributing to the respective reconstructed energy. This is measured by the ratio $\kappa _{E}$ of the electron(photon) energy $E_{e(\gamma )}^{\text {EM}}$ to the jet energy $E_{\text {jet}}^{\text {EM}}$,

8 $$\begin{aligned} \kappa _{E} = \dfrac{E_{e(\gamma )}^{\text {EM}}}{E_{\text {jet}}^{\text {EM}}}, \end{aligned}$$

with both energies calibrated at the EM scale. In the case of $\kappa _{E} \le 50$%, the jet is included in $E_{\mathrm{T}}^{\mathrm{miss}}$ reconstruction, with its $p_{\mathrm{T}}$ scaled by $1-\kappa _{E}$. For $\kappa _{E} > 50$%, only the tracks associated with the jet, excluding the track(s) associated with the overlapping particle if any, contribute to the soft term as discussed in Sect. 3.4.

Jets not rejected by the signal ambiguity resolution and with $p_{\mathrm{T}} > 20\,{\text {Ge}\text {V}}$ and $|\eta | > 2.4$, or with $p_{\mathrm{T}} \ge 60\,{\text {Ge}\text {V}}$ and $|\eta | < 4.5$, are always accepted for $E_{\mathrm{T}}^{\mathrm{miss}}$ reconstruction. Jets reconstructed with $20\,{\text {Ge}\text {V}}< p_{\mathrm{T}} < 60\,{\text {Ge}\text {V}}$ and $|\eta | < 2.4$ are only accepted if they are tagged by $\text {JVT} > 0.59$. In both cases, the jet $p_{\mathrm{T}}$ thresholds are applied to the jet $p_{\mathrm{T}}$ before applying the $\kappa _{E}$ correction. Additional configurations for selecting jets used in $E_{\mathrm{T}}^{\mathrm{miss}}$ reconstruction are discussed in Appendix A, together with the effect of the variation of these selection criteria on the $E_{\mathrm{T}}^{\mathrm{miss}}$ reconstruction performance.

#### Muon overlap with jets

Jets overlapping with a reconstructed muon affect the $E_{\mathrm{T}}^{\mathrm{miss}}$ reconstruction in a manner that depends on their origin. If these jets represent a significant (*catastrophic*) energy loss along the path of the muon through the calorimeter, or if they are pile-up jets tagged by JVT as originating from the hard-scatter interaction due to the muon ID track, they need to be rejected for $E_{\mathrm{T}}^{\mathrm{miss}}$ reconstruction. On the other hand, jets reconstructed from final state radiation (FSR) off the muon need to be included into $E_{\mathrm{T}}^{\mathrm{miss}}$ reconstruction.

In all cases, the muon–jet overlap is determined by ghost-associating the muon with the jet. For this, each muon enters the jet clustering as ghost particle with infinitesimal small momentum, together with the EM-scale topo-clusters from the calorimeter. If a given ghost particle becomes part of a jet, the corresponding muon is considered overlapping with this jet. This procedure is very similar to the track associations with jets mentioned in Sect. 3.3.5.

Tagging jets using JVT efficiently retains those from the hard-scatter vertex for $E_{\mathrm{T}}^{\mathrm{miss}}$ reconstruction and rejects jets generated by pile-up. A muon overlapping with a pile-up jet can lead to a mis-tag, because the ID track from the muon represents a significant amount of $p_{\mathrm{T}}$ from the hard-scatter vertex and thus increases JVT. As a consequence of this fake tag, the pile-up jet $p_{\mathrm{T}}$ contributes to $E_{\mathrm{T}}^{\mathrm{miss}}$, and thus degrades both the $E_{\mathrm{T}}^{\mathrm{miss}}$ response and resolution due to the stochastic nature of its contribution.

A jet that is reconstructed from a catastrophic energy loss of a muon tends to be tagged as a hard-scatter jet as well. This jet is reconstructed from topo-clusters in close proximity to the extrapolated trajectory of the ID track associated with the muon bend in the axial magnetic field. Inclusion of such a jet into $E_{\mathrm{T}}^{\mathrm{miss}}$ reconstruction leads to double-counting of the transverse momentum associated with the muon energy loss, as the fully reconstructed muon $p_{\mathrm{T}}$ is already corrected for this effect.

To reject contributions from pile-up jets and jets reconstructed from muon energy loss, the following selection criteria are applied:

- $p_{\mathrm{T},\text {track}}^{\mu }/p_{\mathrm{T},\text {track}}^{\text {jet}} > 0.8$ – the transverse momentum of the ID track associated with the muon ($p_{\mathrm{T},\text {track}}^{\mu }$) represents a significant fraction of the transverse momentum $p_{\mathrm{T},\text {track}}^{\text {jet}}$, the sum of the transverse momenta of all ID tracks associated with the jet;
- $p_{\mathrm{T}}^{\text {jet}}/p_{\mathrm{T},\text {track}}^{\mu } < 2$ – the overall transverse momentum $p_{\mathrm{T}}^{\text {jet}}$ of the jet is not too large compared to $p_{\mathrm{T},\text {track}}^{\mu }$;
- $N_{\text {track}}^{\mathrm{PV}} < 5$ – the total number of tracks $N_{\text {track}}^{\mathrm{PV}}$ associated with the jet and emerging from the hard-scatter vertex is small.

All jets with overlapping muons meeting these criteria are understood to be either from pile-up or a catastrophic muon energy loss and are rejected for $E_{\mathrm{T}}^{\mathrm{miss}}$ reconstruction. The muons are retained for the $E_{\mathrm{T}}^{\mathrm{miss}}$ reconstruction.

Another consideration for muon contributions to $E_{\mathrm{T}}^{\mathrm{miss}}$ is FSR. Muons can radiate hard photons at small angles, which are typically not reconstructed as such because of the nearby muon ID track violating photon isolation requirements. They are also not reconstructed as electrons, due to the mismatch between the ID track momentum and the energy measured by the calorimeter. Most likely the calorimeter signal generated by the FSR photon is reconstructed as a jet, with the muon ID track associated. As the transverse momentum carried by the FSR photon is not recovered in muon reconstruction, jets representing this photon need to be included in the $E_{\mathrm{T}}^{\mathrm{miss}}$ reconstruction. Such jets are characterised by the following selections, which are highly indicative of a photon in the ATLAS calorimeter:

- $N_{\text {track}}^{\mathrm{PV}} < 3$ – the jet has low charged-particle content, indicated by a very small number of tracks from the hard-scatter vertex;
- $f_{\mathrm{EMC}} > 0.9$ – the jet energy $E^{\text {jet}}$ is largely deposited in the electromagnetic calorimeter (EMC), as expected for photons and measured by the corresponding energy fraction $f_{\mathrm{EMC}} = E^{\text {jet}}_{{\text {EMC}}}/E^{\text {jet}}$;
- $p_{\mathrm{T},\text {PS}}^{\text {jet}} > 2.5\,{\text {Ge}\text {V}}$ – the transverse momentum contribution $p_{\mathrm{T},\text {PS}}^{\text {jet}}$ from presampler signals to $p_{\mathrm{T}}^{\text {jet}}$ indicates an early starting point for the shower;
- $w_{\text {jet}} < 0.1$ – the jet is narrow, with a width $w_{\text {jet}}$ comparable to a dense electromagnetic shower; $w_{\text {jet}}$ is reconstructed according to
  $$\begin{aligned} w_{\text {jet}} = \dfrac{\sum _{i} \Delta R_{i} p_{\mathrm{T},i}}{\sum _{i}p_{\mathrm{T},i}}, \end{aligned}$$
  where $\Delta R_{i}=\sqrt{(\Delta \eta _{i})^{2}+(\Delta \phi _{i})^{2}}$ is the angular distance of topo-cluster *i* from the jet axis, and $p_{\mathrm{T},i}$ is the transverse momentum of this cluster;
- $p_{\mathrm{T},\text {track}}^{\text {jet}}/p_{\mathrm{T},\text {track}}^{\mu } > 0.8$ – the transverse momentum $p_{\mathrm{T},\text {track}}^{\text {jet}}$ carried by all tracks associated with the jet is close to $p_{\mathrm{T},\text {track}}^{\mu }$.

Jets are accepted for $E_{\mathrm{T}}^{\mathrm{miss}}$ reconstruction when consistent with an FSR photon defined by the ensemble of these selection criteria, with their energy scale set to the EM scale, to improve the calibration.

### $E_{\mathrm{T}}^{\mathrm{miss}}$ soft term

The soft term introduced in Sect. 3.2 is exclusively reconstructed from ID tracks from the hard-scatter vertex, thus only using the $p_{\mathrm{T}}$-flow from soft charged particles. It is an important contribution to $E_{\mathrm{T}}^{\mathrm{miss}}$ for the improvement of both the $E_{\mathrm{T}}^{\mathrm{miss}}$ scale and resolution, in particular in final states with a low hard-object multiplicity. In this case it is indicative of (hadronic) recoil, comprising the event components not otherwise represented by reconstructed and calibrated particles or jets.

The more inclusive reconstruction of the $E_{\mathrm{T}}^{\mathrm{miss}}$ soft term including signals from soft neutral particles uses calorimeter topo-clusters. The reconstruction performance using the calorimeter-based $E_{\mathrm{T}}^{{\text {miss}},{\text {soft}},\text {calo}}$ is inferior to the track-only-based $E_{\mathrm{T}}^{{\text {miss}},{\text {soft}}}$, mostly due to a larger residual dependence on pile-up. More details of the topo-cluster-based $E_{\mathrm{T}}^{{\text {miss}},{\text {soft}},\text {calo}}$ reconstruction are discussed in Sect. 8.1.

#### Track and vertex selection

Hits in the ID are used to reconstruct tracks pointing to a particular collision vertex [18]. Both the tracks and vertices need to pass basic quality requirements to be accepted. Each event typically has a number $N_{\mathrm{PV}} > 1$ of reconstructed primary vertices.

Tracks are required to have $p_{\mathrm{T}} > 400\,{\text {Me}\text {V}}$ and $|\eta | < 2.5$, in addition to the reconstruction quality requirements given in Ref. [19]. Vertices are constructed from at least two tracks passing selections on the transverse (longitudinal) impact parameter $|d_{0}| < 1.5\,{\text {mm}}$ ($|z_{0}\sin (\theta )| < 1.5\,{\text {mm}}$) relative to the vertex candidate. These tracks must also pass requirements on the number of hits in the ID. The hard-scatter vertex is identified as described in Appendix A.

#### Track soft term

The track sample contributing to $E_{\mathrm{T}}^{{\text {miss}},{\text {soft}}}$ for each reconstructed event is collected from high-quality tracks emerging from the hard-scatter vertex but not associated with any electron, $\tau \text {-lepton}$, muon, or jet contributing to $E_{\mathrm{T}}^{\mathrm{miss}}$ reconstruction. The applied signal-overlap resolution removes

- ID tracks with $\Delta R(\text {track,electron/photon cluster}) < 0.05$;
- ID tracks with $\Delta R(\text {track},\tau \text {-lepton}) < 0.2$;
- ID tracks associated with muons;
- ID tracks ghost-associated with fully or partially contributing jets.

ID tracks from the hard-scatter vertex that are associated with jets rejected by the overlap removal or are associated with jets that are likely from pile-up, as tagged by the JVT procedure discussed in Sect. 3.3.5, contribute to $E_{\mathrm{T}}^{{\text {miss}},{\text {soft}}}$.

Since only reconstructed tracks associated with the hard-scatter vertex are used, the track-based $E_{\mathrm{T}}^{{\text {miss}},{\text {soft}}}$ is largely insensitive to pile-up effects. It does not include contributions from any soft neutral particles, including those produced by the hard-scatter interaction.

## Data and simulation samples

The determination of the $E_{\mathrm{T}}^{\mathrm{miss}}$ reconstruction performance uses selected final states without ($E_{\mathrm{T}}^{{\text {miss}},\text {true}} = 0$) and with genuine missing transverse momentum from neutrinos ($E_{\mathrm{T}}^{{\text {miss}},\text {true}} = p_{\mathrm{T}}^{\nu }$). Samples with $E_{\mathrm{T}}^{{\text {miss}},\text {true}} = 0$ are composed of leptonic $Z$ boson decays ($Z \rightarrow ee$ and $Z \rightarrow \mu \mu$) collected by a trigger and event selection that do not depend on the particular pile-up conditions, since both the electron and muon triggers as well as the corresponding reconstructed kinematic variables are only negligibly affected by pile-up. Also using lepton triggers, samples with neutrinos were collected from $W \rightarrow e\nu$ and $W \rightarrow \mu \nu$ decays. In addition, samples with neutrinos and higher hard-object multiplicity were collected from top-quark pair ($t\bar{t} \,$) production with at least either the *t* or the $\bar{t}$ decaying semi-leptonically.

### Data samples

The data sample used corresponds to a total integrated luminosity of $3.2~\hbox {fb}^{-1}$, collected with a proton bunch-crossing interval of $25\,{\text {ns}}$. Only high-quality data with a well-functioning calorimeter, inner detector and muon spectrometer are analysed. The data-quality criteria are applied, which reduce the impact of instrumental noise and out-of-time calorimeter deposits from cosmic-ray and beam backgrounds.

### Monte Carlo samples

The $Z \rightarrow \ell \ell$ and $W \rightarrow \ell \nu$ samples were generated using Powheg-Box  [20] (version v1r2856) employing a matrix element calculation at next-to-leading order (NLO) in perturbative QCD. To generate the particle final state, the (parton-level) matrix element output was interfaced to Pythia8  [21],^5^ which generated the parton shower (PS) and the underlying event (UE) using the AZNLO tuned parameter set [22]. Parton distribution functions (PDFs) were taken from the CTEQ6L1 PDF set [23].

The $t\bar{t}$-production sample was generated with a Powheg NLO kernel (version v2r3026) interfaced to Pythia6  [24] (version 6.428) with the Perugia2012 set of tuned parameters [25] for the PS and UE generation. The CT10 NLO PDF set [26] was employed. The resummation of soft-gluon terms in the next-to-next-to-leading-logarithmic (NNLL) approximation with top++  2.0  [27] was included.

Additional processes contributing to the $Z \rightarrow \ell \ell$ and $W \rightarrow \ell \nu$ final state samples are the production of dibosons, single top quarks, and multijets. Dibosons were generated using Sherpa [28–31] version v2.1.1 employing the CT10 PDF set. Single top quarks were generated using Powheg version v1r2556 with the CT10 PDF set for the *t*-channel production and Powheg version v1r2819 for the *s*-channel and the associated top quark ($W t$) production, all interfaced to the PS and UE from the same Pythia6 configuration used for $t\bar{t}$ production. Multijet events were generated using Pythia8 with the NNPDF23LO PDF set [32] and the A14 set of tuned PS and UE parameters described in Ref. [33].

Minimum bias (MB) events were generated using Pythia8 with the MSTW2008LO PDF set [34] and the A2 tuned parameter set [35] for PS and UE. These MB events were used to model pile-up, as discussed in Sect. 4.3.

For the determination of the systematic uncertainties in $E_{\mathrm{T}}^{\mathrm{miss}}$ reconstruction, an alternative inclusive sample of $Z \rightarrow \mu \mu$ events was generated using the MadGraph_aMC@NLO (version v2.2.2) matrix element generator [36] employing the CTEQ6L1 PDF set. Both PS and UE were generated using Pythia8 with the NNPDF23LO PDF set and the A14 set of tuned parameters.

The MC-generated events were processed with the Geant4 software toolkit [37], which simulates the propagation of the generated stable particles^6^ through the ATLAS detector and their interactions with the detector material [38].

### Pile-up

The calorimeter signals are affected by pile-up and the short bunch-crossing period at the LHC. In 2015, an average of about 13 pile-up collisions per bunch crossing was observed. The dominant contribution of the additional $pp$ collisions to the detector signals of the recorded event arises from a diffuse emission of soft particles superimposed to the hard-scatter interaction final state (in-time pile-up). In addition, the LAr calorimeter signals are sensitive to signal remnants from up to 24 previous bunch crossings and one following bunch crossing (out-of-time pile-up), as discussed in Refs. [6, 39]. Both types of pile-up affect signals contributing to $E_{\mathrm{T}}^{\mathrm{miss}}$.

The in-time pile-up activity is measured by the number of reconstructed primary collision vertices $N_{\mathrm{PV}}$. The out-of-time pile-up is proportional to the number of collisions per bunch crossing $\mu$, measured as an average over time periods of up to two minutes by integrated signals from the luminosity detectors in ATLAS [40].

To model in-time pile-up in MC simulations, a number of generated pile-up collisions was drawn from a Poisson distribution around the value of $\mu$ recorded in data. The collisions were randomly collected from the MB sample discussed in Sect. 4.2. The particles emerging from them were overlaid onto the particle-level final state of the generated hard-scatter interaction and converted into detector signals before event reconstruction. The event reconstruction then proceeds as for data.

Similar to the LHC proton-beam structure, events in MC simulations are organised in bunch trains, where the structure in terms of bunch-crossing interval and gaps between trains is taken into account to model the effects of out-of-time pile-up. The fully reconstructed events in MC simulation samples are finally weighted such that the distribution of the number of overlaid collisions over the whole sample corresponds to the $\mu$ distribution observed in data.

The effect of pile-up on the signal in the Tile calorimeter is reduced due to its location behind the electromagnetic calorimeter and its fast time response [41]. Reconstructed ID and MS tracks are largely unaffected by pile-up.

## Event selection

### $Z \rightarrow \mu \mu$ event selection

The $Z \rightarrow \mu \mu$ final state is ideal for the evaluation of $E_{\mathrm{T}}^{\mathrm{miss}}$ reconstruction performance, since it can be selected with a high signal-to-background ratio and the $Z$ kinematics can be measured with high precision, even in the presence of pile-up. Neutrinos are produced only through very rare heavy-flavour decays in the hadronic recoil. This channel can therefore be considered to have no genuine missing transverse momentum. Thus, the scale and resolution for the reconstructed $E_{\mathrm{T}}^{\mathrm{miss}}$ are indicative of the reconstruction quality and reflect limitations introduced by both the detector and the ambiguity resolution procedure. The well-defined expectation value $E_{\mathrm{T}}^{{\text {miss}},\text {true}} = 0$ allows the reconstruction quality to be determined in both data and MC simulations. The reconstructed $E_{\mathrm{T}}^{\mathrm{miss}}$ in this final state is also sensitive to the effectiveness of the muon–jet overlap resolution, which can be explored in this low-multiplicity environment in both data and MC simulations, with a well-defined $E_{\mathrm{T}}^{\mathrm{miss}}$.

Events must pass one of three high-level muon triggers with different $p_{\mathrm{T}}^{\mu }$ thresholds and isolation requirements. The isolation is determined by the ratio of the scalar sum of $p_{\mathrm{T}}$ of reconstructed tracks other than the muon track itself, in a cone of size $\Delta R = 0.2$ around the muon track ($p_{\mathrm{T}}^{\text {cone}}$), to $p_{\mathrm{T}}^{\mu }$. The individual triggers require (1) $p_{\mathrm{T}}^{\mu } > 20\,{\text {Ge}\text {V}}$ and $p_{\mathrm{T}}^{\text {cone}}/p_{\mathrm{T}}^{\mu } < 0.12$, or (2) $p_{\mathrm{T}}^{\mu } > 24\,{\text {Ge}\text {V}}$ and $p_{\mathrm{T}}^{\text {cone}}/p_{\mathrm{T}}^{\mu } < 0.06$, or (3) $p_{\mathrm{T}}^{\mu } > 50\,{\text {Ge}\text {V}}$ without isolation requirement.

The offline selection of $Z \rightarrow \mu \mu$ events requires exactly two muons, each selected as defined in Sect. 3.3.4, with the additional criteria that (1) the muons must have opposite charge, (2) $p_{\mathrm{T}}^{\mu } > 25\,{\text {Ge}\text {V}}$, and (3) the reconstructed invariant mass $m_{\mu \mu }$ of the dimuon system is consistent with the mass $m_{Z}$ of the $Z$ boson, $|m_{\mu \mu }- m_{Z} | < 25\,{\text {Ge}\text {V}}$.

### $W \rightarrow e\nu$ event selection

Events with $W \rightarrow e\nu$ or $W \rightarrow \mu \nu$ in the final state provide a well-defined topology with neutrinos produced in the hard-scatter interaction. In combination with $Z \rightarrow \mu \mu$, the effectiveness of signal ambiguity resolution and lepton energy reconstruction for both the electrons and muons can be observed. The $W \rightarrow e\nu$ events in particular provide a good metric with $E_{\mathrm{T}}^{{\text {miss}},\text {true}} = p_{\mathrm{T}}^{\nu } > 0$ to evaluate and validate the scale, resolution and direction (azimuth) of the reconstructed $E_{\mathrm{T}}^{\mathrm{miss}}$, as the $E_{\mathrm{T}}^{\mathrm{miss}}$ reconstruction is sensitive to the electron–jet overlap resolution performance. This metric is only available in MC simulations where $p_{\mathrm{T}}^{\nu }$ is known. Candidate $W \rightarrow e\nu$ events are required to pass the high-level electron trigger with $p_{\mathrm{T}} > 17\,{\text {Ge}\text {V}}$. Electron candidates are selected according to criteria described in Sect. 3.3.1. Only events containing exactly one electron are considered.

Further selections using $E_{\mathrm{T}}^{\mathrm{miss}}$ and the reconstructed transverse mass $m_{\mathrm{T}}$, given by

$$\begin{aligned} m_{\mathrm{T}} = \sqrt{ 2p_{\mathrm{T}}^{e} E_{\mathrm{T}}^{\mathrm{miss}} (1-\cos {\Delta \phi })}, \end{aligned}$$

are applied to reduce the multijet background with one jet emulating an isolated electron from the $W$ boson. Here $E_{\mathrm{T}}^{\mathrm{miss}}$ is calculated as presented in Sect. 3. The transverse momentum of the electron is denoted by $p_{\mathrm{T}}^{e}$, and $\Delta \phi$ is the distance between $\phi ^{{\text {miss}}}$ and the azimuth of the electron. Selected events are required to have $E_{\mathrm{T}}^{\mathrm{miss}} > 25\,{\text {Ge}\text {V}}$ and $m_{\mathrm{T}} > 50\,{\text {Ge}\text {V}}$.

### $t\bar{t}$ event selection

Events with $t\bar{t}$ in the final state allow the evaluation of the $E_{\mathrm{T}}^{\mathrm{miss}}$ performance in interactions with a large jet multiplicity. Electrons and muons used to define these samples are reconstructed as discussed in Sects. 3.3.1 and 3.3.4, respectively, and are required to have $p_{\mathrm{T}} > 25\,{\text {Ge}\text {V}}$.

The final $t\bar{t}$ sample is selected by imposing additional requirements. Each event must have exactly one electron and no muons passing the selections described above. In addition, at least four jets reconstructed by the anti-$k_{t}$ algorithm with $R = 0.4$ and selected following the description in Sect. 3.3.5 are required. At least one of the jets needs to be *b*-tagged using the tagger configuration for a 77% efficiency working point described in Ref. [42]. All jets are required to be at an angular distance of $\Delta R > 0.4$ from the electron.

## Performance of $E_{\mathrm{T}}^{\mathrm{miss}}$ reconstruction in data and Monte Carlo simulation

Unlike for fully reconstructed and calibrated particles and jets, and in the case of the precise reconstruction of charged particle kinematics provided by ID tracks, $E_{\mathrm{T}}^{\mathrm{miss}}$ reconstruction yields a non-linear response, especially in regions of phase space where the observation bias discussed in Sect. 3.1 dominates the reconstructed $E_{\mathrm{T}}^{\mathrm{miss}}$. In addition, the $E_{\mathrm{T}}^{\mathrm{miss}}$ resolution functions are characterised by a high level of complexity, due to the composite character of the observable. Objects with different $p_{\mathrm{T}}$-resolutions contribute, and the $E_{\mathrm{T}}^{\mathrm{miss}}$ composition can fluctuate significantly for events from the same final state. Due to the dependence of the $E_{\mathrm{T}}^{\mathrm{miss}}$ response on the resolution, both performance characteristics change as a function of the total event activity and are affected by pile-up. There is no universal way of mitigating these effects, due to the inability to validate in data a stable and universal calibration reference for $E_{\mathrm{T}}^{\mathrm{miss}}$.

The $E_{\mathrm{T}}^{\mathrm{miss}}$ reconstruction performance is therefore assessed by comparing a set of reconstructed $E_{\mathrm{T}}^{\mathrm{miss}}$-related observables in data and MC simulations for the same final-state selection, with the same object and event selections applied. Systematic uncertainties in the $E_{\mathrm{T}}^{\mathrm{miss}}$ response and resolution are derived from these comparisons and are used to quantify the level of understanding of the data from the physics models. The quality of the detector simulation is independently determined for all reconstructed jets, particles and ID tracks, and can thus be propagated to the overall $E_{\mathrm{T}}^{\mathrm{miss}}$ uncertainty for any given event. Both the distributions of observables as well as their average behaviour with respect to relevant scales measuring the overall kinematic activity of the hard-scatter event or the pile-up activity are compared. To focus on distribution shapes rather than statistical differences in these comparisons, the overall distribution of a given observable obtained from MC simulations is normalised to the integral of the corresponding distribution in data.

As the reconstructed final state can be produced by different physics processes, the individual process contributions in MC simulations are scaled according to the cross section of the process. This approach is taken to both show the contribution of a given process to the overall distribution, and to identify possible inadequate modelling arising from any individual process, or a subset of processes, by its effect on the overall shape of the MC distribution.

Inclusive event samples considered for the $E_{\mathrm{T}}^{\mathrm{miss}}$ performance evaluation are obtained by applying selections according to Sect. 5.1 for a final state without genuine $E_{\mathrm{T}}^{\mathrm{miss}}$ ($Z \rightarrow \mu \mu$), and according to Sect. 5.2 for a final state with genuine $E_{\mathrm{T}}^{\mathrm{miss}}$ ($W \rightarrow e\nu$). From these, specific exclusive samples are extracted by applying conditions on the number of jets reconstructed. In particular, *zero jet* ($N_{\mathrm{jet}} = 0$) samples without any jet with $p_{\mathrm{T}} > 20\,{\text {Ge}\text {V}}$ (fully calibrated) and $|\eta |<4.9$ are useful for exclusively studying the performance of the soft term. Samples with events selected on the basis of a non-zero number of reconstructed jets with $p_{\mathrm{T}} > 20\,{\text {Ge}\text {V}}$ are useful for evaluating the contribution of jets to $E_{\mathrm{T}}^{\mathrm{miss}}$. While the $p_{\mathrm{T}}$ response of jets is fully calibrated and provides a better measurement of the overall event $p_{\mathrm{T}}$-flow, the $p_{\mathrm{T}}$ resolution for jets is affected by pile-up and can introduce a detrimental effect on $E_{\mathrm{T}}^{\mathrm{miss}}$ reconstruction performance.

Missing transverse momentum and its related observables presented in Sect. 3.1 are reconstructed for the performance evaluations shown in the following sections using a standard reconstruction configuration. This configuration implements the signal ambiguity resolution in the $E_{\mathrm{T}}^{\mathrm{miss}}$ reconstruction sequence discussed in Sect. 3.1. It employs the hard-object selections defined in Sects. 3.3.1–3.3.4, with jets selected according to the prescriptions given in Sect. 3.3.5. The overlap resolution strategy for jets and muons described in Sect. 3.3.6 is applied. The soft term is formed from ID tracks according to Sect. 3.4.

### $E_{\mathrm{T}}^{\mathrm{miss}}$ modelling in Monte Carlo simulations

**Fig. 1:**
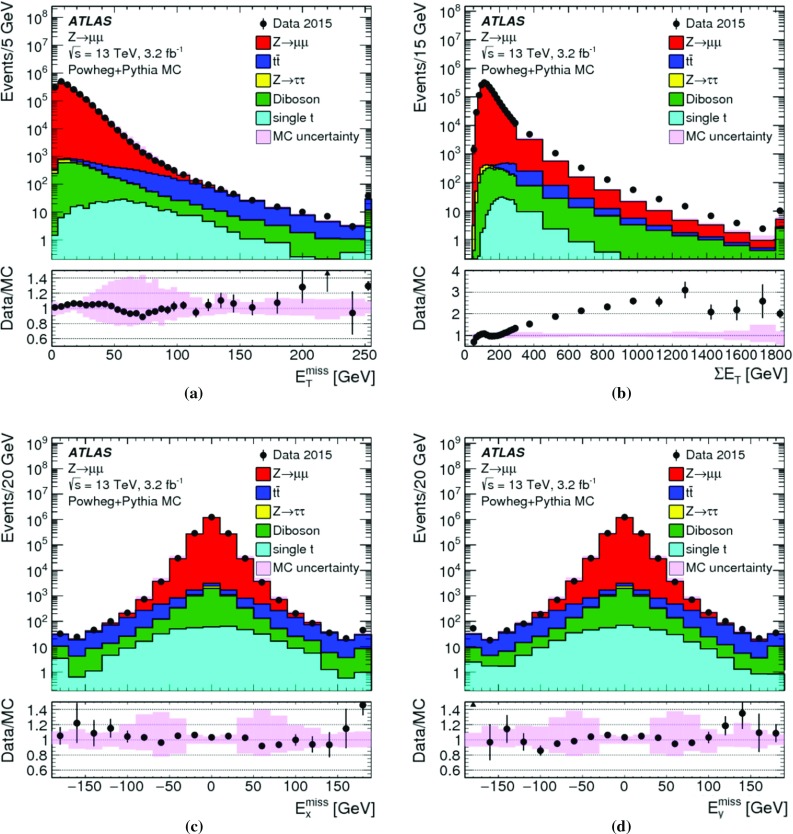
Distributions of **a** $E_{\mathrm{T}}^{\mathrm{miss}}$, **b** $\Sigma E_{\mathrm{T}}$, **c** $E_{x}^{{\text {miss}}}$ and **d** $E_{y}^{{\text {miss}}}$ for an inclusive sample of $Z \rightarrow \mu \mu$ events extracted from data and compared to MC simulations including all relevant backgrounds. The shaded areas indicate the total uncertainty for MC simulations, including the overall statistical uncertainty combined with systematic uncertainties from the $p_{\mathrm{T}}$ scale and resolution which are contributed by muons, jets, and the soft term. The last bin of each distribution includes the overflow, and the first bin contains the underflow in **c** and **d**. The respective ratios between data and MC simulations are shown below the distributions, with the shaded areas showing the total uncertainties for MC simulations

**Fig. 2:**
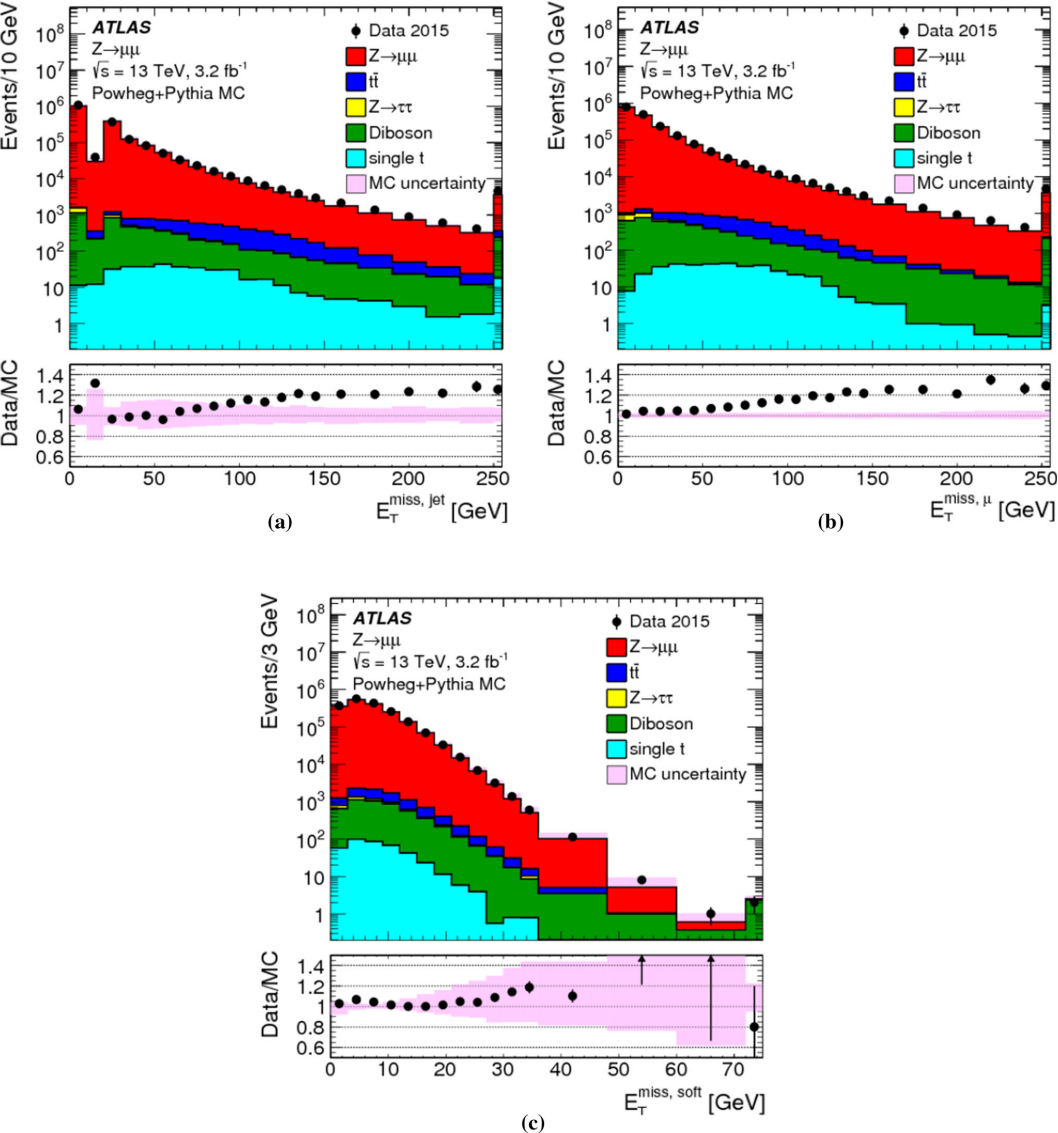
Distributions of **a** the jet term $E_{\mathrm{T}}^{{\text {miss}},\text {jet}}$, **b** the muon term $E_{\mathrm{T}}^{{\text {miss}},\mu }$, and **c** the soft term $E_{\mathrm{T}}^{{\text {miss}},{\text {soft}}}$ for the inclusive samples of $Z \rightarrow \mu \mu$ events in data, compared to MC simulations including all relevant backgrounds. The shaded areas indicate the total uncertainty from MC simulations, including the overall statistical uncertainty combined with the respective systematic uncertainties from **a** the jet, **b** the muon, and **c** the soft term. The last bin of each distribution includes the overflow entries. The respective ratios between data and MC simulations are shown below the distributions, with the shaded areas showing the corresponding total uncertainties from MC simulations

The quality of the MC modelling of $E_{x}^{{\text {miss}}}$, $E_{y}^{{\text {miss}}}$, $E_{\mathrm{T}}^{\mathrm{miss}}$ and $\Sigma E_{\mathrm{T}}$, reconstructed as given in Eqs. (1), (3) and (5), is evaluated for an inclusive sample of $Z \rightarrow \mu \mu$ events by comparing the distributions of these observables to data. The results are presented in Fig. 1. The data and MC simulations agree within 20% for the bulk of the $E_{\mathrm{T}}^{\mathrm{miss}}$ distribution shown in Fig. 1a, with larger differences not accommodated by the total (systematic and statistical) uncertainties of the distributions for high $E_{\mathrm{T}}^{\mathrm{miss}}$. These differences suggest a mismodelling in $t\bar{t}$ events, the dominant background in the tail regime [43]. The $\Sigma E_{\mathrm{T}}$ distributions compared between data and MC simulations in Fig. 1b show discrepancies significantly larger than the overall uncertainties for $200\,{\text {Ge}\text {V}}< \Sigma E_{\mathrm{T}} < 1.2\,{\text {Te}\text {V}}$. These reflect the level of mismodelling of the final state mostly in terms of hard-object composition in MC simulations. The $E_{x}^{{\text {miss}}}$ and $E_{y}^{{\text {miss}}}$ spectra shown in Fig. 1c, d, respectively, show good agreement between data and MC simulations for the bulk of the distributions within $|E_{x(y)}^{{\text {miss}}} |<100\,{\text {Ge}\text {V}}$, with larger differences observed outside of this range still mostly within the uncertainties.

**Fig. 3:**
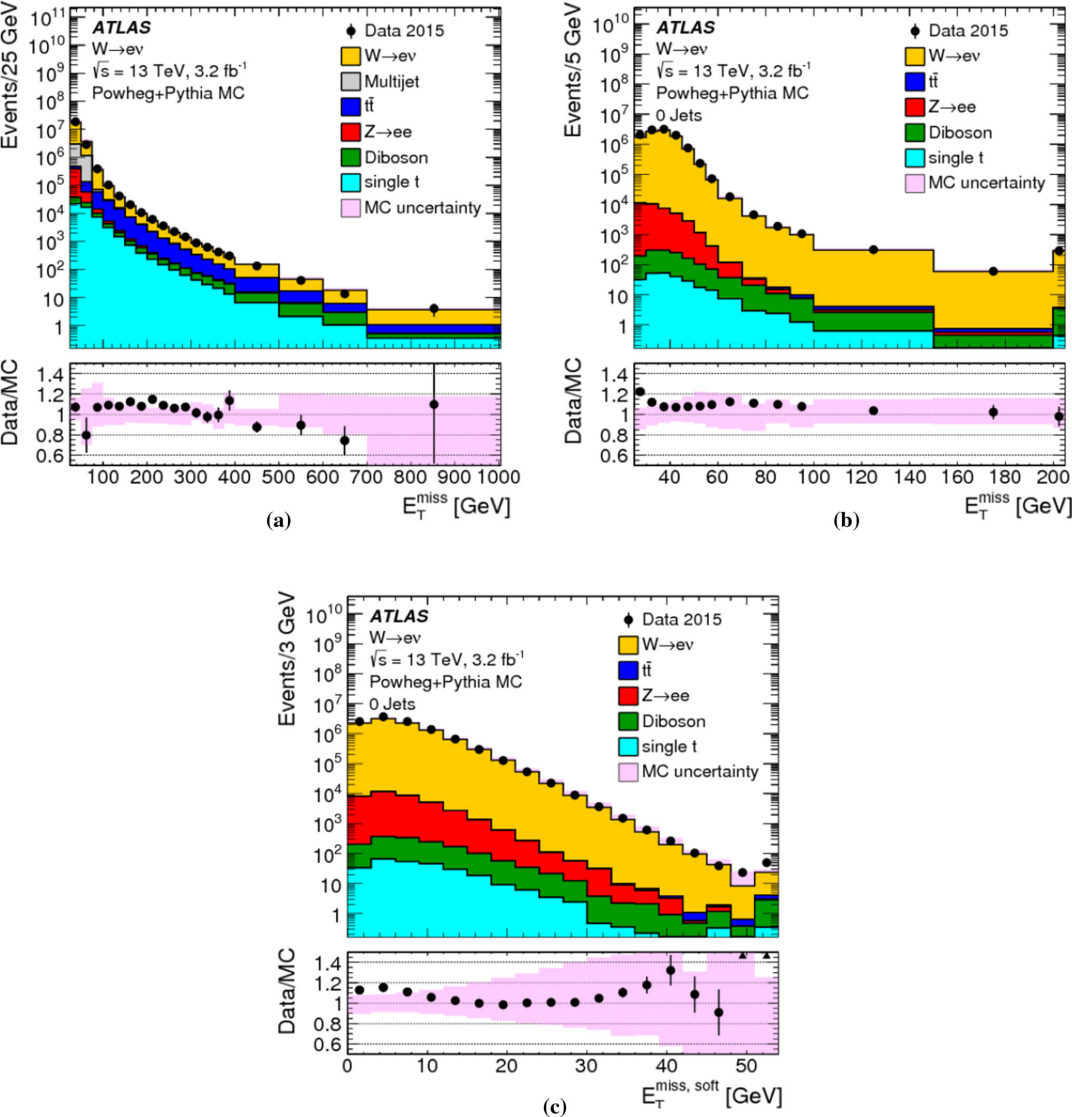
Distributions of the total $E_{\mathrm{T}}^{\mathrm{miss}}$ in **a** the inclusive case and **b** the $N_{\mathrm{jet}} = 0$ case, as well as **c** the soft term $E_{\mathrm{T}}^{{\text {miss}},{\text {soft}}}$ reconstructed in $N_{\mathrm{jet}} = 0$ events with $W \rightarrow e\nu$ in data. The expectation from MC simulation is superimposed and includes all relevant background final states passing the event selection. The inclusive $E_{\mathrm{T}}^{\mathrm{miss}}$ distribution from MC simulations contains a small contribution from multijet final states at low $E_{\mathrm{T}}^{\mathrm{miss}}$, which is absent for the $N_{\mathrm{jet}} = 0$ selection. The shaded areas indicate the total uncertainty for MC simulations, including the overall statistical uncertainty combined with systematic uncertainties comprising contributions from the electron, jet, and the soft term. The last bins contain the respective overflows. The respective ratios between data and MC simulations are shown below the distributions, with the shaded areas indicating the total uncertainties for MC simulations

The distributions of individual contributions to $E_{\mathrm{T}}^{\mathrm{miss}}$ from jets ($E_{\mathrm{T}}^{{\text {miss}},\text {jet}}$), muons ($E_{\mathrm{T}}^{{\text {miss}},\mu }$), and the soft term ($E_{\mathrm{T}}^{{\text {miss}},{\text {soft}}}$), as defined in Eq. (6), are compared between data and MC simulations for the same inclusive $Z \rightarrow \mu \mu$ sample in Fig. 2. Agreement between data and MC simulations for $E_{\mathrm{T}}^{{\text {miss}},\text {jet}}$ in Fig. 2a is of the order of $\pm 20$% and within the total uncertainties for $E_{\mathrm{T}}^{{\text {miss}},\text {jet}} \lesssim 120\,{\text {Ge}\text {V}}$, but beyond those for higher $E_{\mathrm{T}}^{{\text {miss}},\text {jet}}$. A similar observation holds for $E_{\mathrm{T}}^{{\text {miss}},\mu }$ in Fig. 2b, where data and MC simulations agree within the uncertainties for low $E_{\mathrm{T}}^{{\text {miss}},\mu }$ but significantly beyond them for larger $E_{\mathrm{T}}^{{\text {miss}},\mu }$. Agreement between data and MC simulations is better for the soft term $E_{\mathrm{T}}^{{\text {miss}},{\text {soft}}}$, with differences up to 10% for $E_{\mathrm{T}}^{{\text {miss}},{\text {soft}}} \lesssim 30\,{\text {Ge}\text {V}}$, as seen in Fig. 2c. Larger differences for larger $E_{\mathrm{T}}^{{\text {miss}},{\text {soft}}}$ are still found to be within the uncertainties.

The peak around $E_{\mathrm{T}}^{{\text {miss}},\text {jet}} = 20\,{\text {Ge}\text {V}}$ indicates the onset of single-jet events at the threshold $p_{\mathrm{T}} = 20\,{\text {Ge}\text {V}}$ for jets contributing to $E_{\mathrm{T}}^{{\text {miss}},\text {jet}}$. Larger values of $E_{\mathrm{T}}^{{\text {miss}},\text {jet}}$ arise from events with one or more high-$p_{\mathrm{T}}$ jets balancing the $p_{\mathrm{T}}$ of the $Z$ boson.

For the $W \rightarrow e\nu$ sample with genuine missing transverse momentum given by $p_{\mathrm{T}}^{\nu }$, both the total reconstructed $E_{\mathrm{T}}^{\mathrm{miss}}$ and the soft term are compared between data and MC simulations in Fig. 3. The level of agreement between the $E_{\mathrm{T}}^{\mathrm{miss}}$ distributions for data and MC simulations shown in Fig. 3a for the inclusive event sample is at $\pm 20\%$, similar to that observed for the $Z \rightarrow \mu \mu$ sample in Fig. 1a, except that for this final state it is found to be within the total uncertainties of the measurement. The differences between the $E_{\mathrm{T}}^{\mathrm{miss}}$ distributions observed with the exclusive $N_{\mathrm{jet}} = 0$ sample shown in Fig. 3b are well below 20%, but show a trend to larger discrepancies for decreasing $E_{\mathrm{T}}^{\mathrm{miss}} \lesssim 40\,{\text {Ge}\text {V}}$. This trend is due to the missing background contribution in MC simulations from multijet final states. The extraction of this contribution is very inefficient and only possible with large statistical uncertainties. Even very large MC samples of multijet final states provide very few events with only one jet that is accidentally reconstructed as an electron, and with the amount of $E_{\mathrm{T}}^{\mathrm{miss}}$ required in the $W \rightarrow e\nu$ selection described in Sect. 5.2. The comparison of the $E_{\mathrm{T}}^{{\text {miss}},{\text {soft}}}$ distributions from data and MC simulations shown in Fig. 3c yields agreement well within the uncertainties, for $E_{\mathrm{T}}^{{\text {miss}},{\text {soft}}} \gtrsim 10\,{\text {Ge}\text {V}}$. The rising deficiencies observed in the MC distribution for decreasing $E_{\mathrm{T}}^{{\text {miss}},{\text {soft}}} \lesssim 10\,{\text {Ge}\text {V}}$ are expected to be related to the missing multijet contribution.

### $E_{\mathrm{T}}^{\mathrm{miss}}$ response and resolution

The response in the context of $E_{\mathrm{T}}^{\mathrm{miss}}$ reconstruction is determined by the deviation of the observed $E_{\mathrm{T}}^{\mathrm{miss}}$ from the expectation value for a given final state. This deviation sets the scale for the observed $E_{\mathrm{T}}^{\mathrm{miss}}$. If this deviation is independent of the genuine missing transverse momentum, or any other hard $p_{\mathrm{T}}$ indicative of the overall hard-scatter activity, the $E_{\mathrm{T}}^{\mathrm{miss}}$ response is linear. In this case, a constant bias in the reconstructed $E_{\mathrm{T}}^{\mathrm{miss}}$ is still possible due to detector inefficiencies and coverage (acceptance) limitations.

Final states balanced in transverse momentum are expected to show a non-linear $E_{\mathrm{T}}^{\mathrm{miss}}$ response at low event activity, as the response in this case suffers from the observation bias in $E_{\mathrm{T}}^{\mathrm{miss}}$ reconstruction discussed in Sect. 3.1. With increasing momentum transfers in the hard-scatter interaction, the $E_{\mathrm{T}}^{\mathrm{miss}}$ response becomes increasingly dominated by a well-measured hadronic recoil and thus more linear. In the case of final states with genuine missing transverse momentum, the $E_{\mathrm{T}}^{\mathrm{miss}}$ response is only linear once $E_{\mathrm{T}}^{{\text {miss}},\text {true}}$ exceeds the observation bias. These features are discussed in Sect. 6.2.1 and explored in Sect. 6.2.2.

Contributions to the fluctuations in the $E_{\mathrm{T}}^{\mathrm{miss}}$ measurement arise from (1) the limitations in the detector acceptance not allowing the reconstruction of the complete transverse momentum flow from the hard interaction, (2) the irreducible intrinsic signal fluctuations in the detector response, and from (3) the additional response fluctuations due to pile-up. In particular (1) introduces fluctuations driven by the large variations of the particle composition of the final state with respect to their types, momenta and directions. The limited detector coverage of $|\eta | < 4.9$ for all particles, together with the need to suppress the pile-up-induced signal fluctuations as much as possible, restricts the contribution of particles to $E_{\mathrm{T}}^{\mathrm{miss}}$ to the reconstructed and accepted $e$, $\gamma$, $\tau _{\mathrm{had}}$ and $\mu$, and those being part of a reconstructed and accepted jet. In addition, the $p_{\mathrm{T}}$-flow of not explicitly reconstructed charged particles emerging from the hard-scatter vertex is represented by ID tracks contributing to $E_{\mathrm{T}}^{{\text {miss}},{\text {soft}}}$ given in Eqs. (6) and (7), but only in the phase space defined by the selections given in Sect. 3.4.1. All other charged and neutral particles do not contribute to $E_{\mathrm{T}}^{\mathrm{miss}}$ reconstruction.

Like for the $E_{\mathrm{T}}^{\mathrm{miss}}$ response, resolution-related aspects of $E_{\mathrm{T}}^{\mathrm{miss}}$ reconstruction are understood from data-to-MC-simulations comparisons. The scales used for the corresponding evaluations are the overall event activity represented by $\Sigma E_{\mathrm{T}}$, and the pile-up activity measured by $N_{\mathrm{PV}}$. The measurement of the $E_{\mathrm{T}}^{\mathrm{miss}}$ resolution is discussed in Sect. 6.2.3 and results are presented in Sect. 6.2.4.

#### $E_{\mathrm{T}}^{\mathrm{miss}}$ scale determination

In events with $Z \rightarrow \mu \mu$ decays, the transverse momentum of the $Z$ boson ($p_{\mathrm{T}}^{Z}$) is an indicator of the hardness of the interaction. It provides a useful scale for the evaluation of the $E_{\mathrm{T}}^{\mathrm{miss}}$ response for this final state without genuine missing transverse momentum. The direction of the corresponding $Z$ boson transverse momentum vector $\mathbf p _{\mathrm{T}}^{Z}$ defines an axis $\mathbf A _{Z}$ in the transverse plane of the collision, which is reconstructed from the $\mathbf p _{\mathrm{T}}$ of the decay products by9The magnitude of the component of $\mathbf E _{\mathrm{T}}^{{\text {miss}}}$ parallel to $\mathbf A _{Z}$ is

10 $$\begin{aligned} \mathcal {P} _{\parallel }^{\,\!Z} = \mathbf E _{\mathrm{T}}^{{\text {miss}}} \cdot \mathbf A _{Z} . \end{aligned}$$

This projection is sensitive to any limitation in $E_{\mathrm{T}}^{\mathrm{miss}}$ reconstruction, in particular with respect to the contribution from the hadronic recoil against $\mathbf p _{\mathrm{T}}^{Z}$, both in terms of response and resolution. Because it can be determined both for data and MC simulations, it provides an important tool for the validation of the $E_{\mathrm{T}}^{\mathrm{miss}}$ response and the associated systematic uncertainties.

The expectation value for a balanced interaction producing a $Z$ boson against a hadronic recoil is $\text {E}[\mathcal {P} _{\parallel }^{\,\!Z} ] = 0$. Any observed deviation from this value represents a bias in the $E_{\mathrm{T}}^{\mathrm{miss}}$ reconstruction. For $\mathcal {P} _{\parallel }^{\,\!Z} < 0$, the reconstructed hadronic activity recoiling against $\mathbf p _{\mathrm{T}}^{Z}$ is too small, while for $\mathcal {P} _{\parallel }^{\,\!Z} > 0$ too much hadronic recoil is reconstructed. The evolution of $\mathcal {P} _{\parallel }^{\,\!Z}$ as a function of the hardness of the $Z$ boson production can be measured by evaluating the mean $\langle \mathcal {P} _{\parallel }^{\,\!Z} \rangle$ in bins of the hard-scatter scale $p_{\mathrm{T}}^{{\text {hard}}} = p_{\mathrm{T}}^{Z}$.

In addition to measuring the $E_{\mathrm{T}}^{\mathrm{miss}}$ response in data and MC simulation without genuine $E_{\mathrm{T}}^{\mathrm{miss}}$, its linearity can be determined using samples of final states with genuine $E_{\mathrm{T}}^{\mathrm{miss}}$ in MC simulations. This is done by evaluating the relative deviation $\Delta _{\mathrm{T}}^{\mathrm{lin}}$ of the reconstructed $E_{\mathrm{T}}^{\mathrm{miss}}$ from the expected $E_{\mathrm{T}}^{{\text {miss}},\text {true}} > 0$ as a function of $E_{\mathrm{T}}^{{\text {miss}},\text {true}}$,

11 $$\begin{aligned} \Delta _{\mathrm{T}}^{\mathrm{lin}} (E_{\mathrm{T}}^{{\text {miss}},\text {true}}) = \dfrac{E_{\mathrm{T}}^{\mathrm{miss}}- E_{\mathrm{T}}^{{\text {miss}},\text {true}}}{E_{\mathrm{T}}^{{\text {miss}},\text {true}}}. \end{aligned}$$

#### Measuring the $E_{\mathrm{T}}^{\mathrm{miss}}$ response

**Fig. 4:**
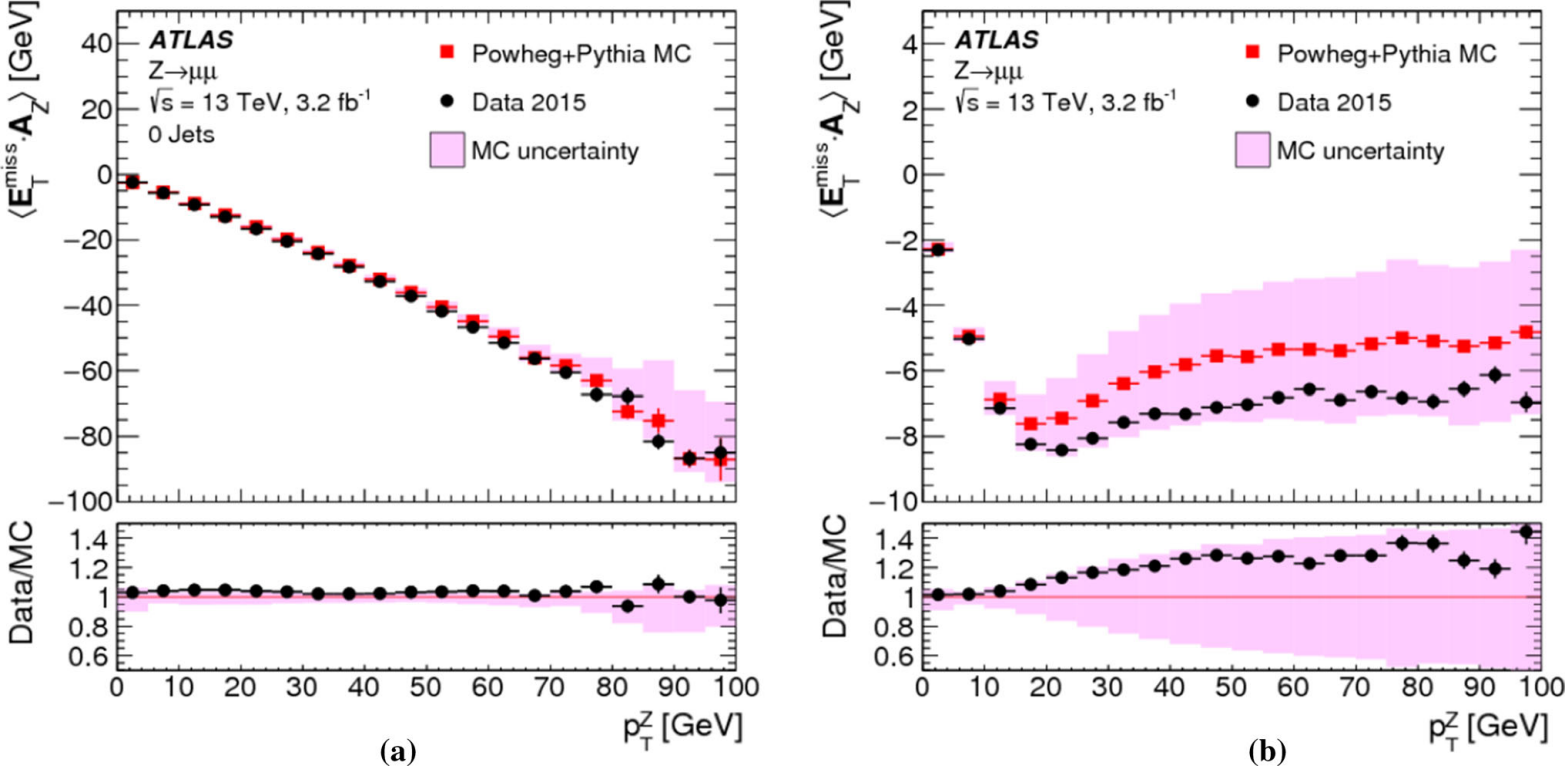
The average projection of $\mathbf E _{\mathrm{T}}^{{\text {miss}}}$ onto the direction $\mathbf A _{Z}$ of the $Z$ boson’s transverse momentum vector $\mathbf p _{\mathrm{T}}^{Z}$, as given in Eq. (10), is shown as a function of $p_{\mathrm{T}}^{Z} = |\mathbf p _{\mathrm{T}}^{Z} |$ in $Z \rightarrow \mu \mu$ events from **a** the $N_{\mathrm{jet}} = 0$ sample and from **b** the inclusive sample. In both cases data are compared to MC simulations. The ratio of the averages from data and MC simulations are shown below the plots. The shaded areas indicate the overall statistical uncertainty combined with systematic uncertainties comprising contributions from the muon and soft-term systematic uncertainties in **a**, and including the additional jet systematic uncertainties in **b**, for MC simulations

Figure 4 shows $\langle \mathcal {P} _{\parallel }^{\,\!Z} \rangle$ as a function of $p_{\mathrm{T}}^{Z}$ for the $N_{\mathrm{jet}} = 0$ and the inclusive $Z \rightarrow \mu \mu$ sample, respectively. MC simulations compare well with the data for $N_{\mathrm{jet}} = 0$, but show larger deviations up to 30% for the inclusive selection. Nevertheless, these differences are still found to be within the total uncertainty of the measurement.

The steep decrease of $\langle \mathcal {P} _{\parallel }^{\,\!Z} \rangle$ with increasing $p_{\mathrm{T}}^{Z}$ in the $N_{\mathrm{jet}} = 0$ sample seen in Fig. 4a reflects the inherent underestimation of the soft term, as in this case the hadronic recoil is exclusively represented by ID tracks with $p_{\mathrm{T}} > 400\,{\text {Me}\text {V}}$ within $|\eta | < 2.5$. It thus does not contain any signal from (1) neutral particles, (2) charged particles produced with $|\eta | > 2.5$, and (3) charged particles produced within $|\eta | < 2.5$ but with $p_{\mathrm{T}}$ below threshold, rejected by the track quality requirements, or not represented by a track at all due to insufficient signals in the ID (e.g., lack of hits for track fitting).

In the case of the inclusive sample shown in Fig. 4b, the $E_{\mathrm{T}}^{\mathrm{miss}}$ response is recovered better as $p_{\mathrm{T}}^{Z}$ increases, since an increasing number of events enter the sample with a reconstructed recoil containing fully calibrated jets. These provide a more complete representation of the hadronic transverse momentum flow. The residual offsets in $\langle \mathcal {P} _{\parallel }^{\,\!Z} \rangle$ of about $8\,{\text {Ge}\text {V}}$ in data and $6\,{\text {Ge}\text {V}}$ in MC simulations observed for $p_{\mathrm{T}}^{Z} \gtrsim 40\,{\text {Ge}\text {V}}$ in Fig. 4b agree within the uncertainties of this measurement.

**Fig. 5:**
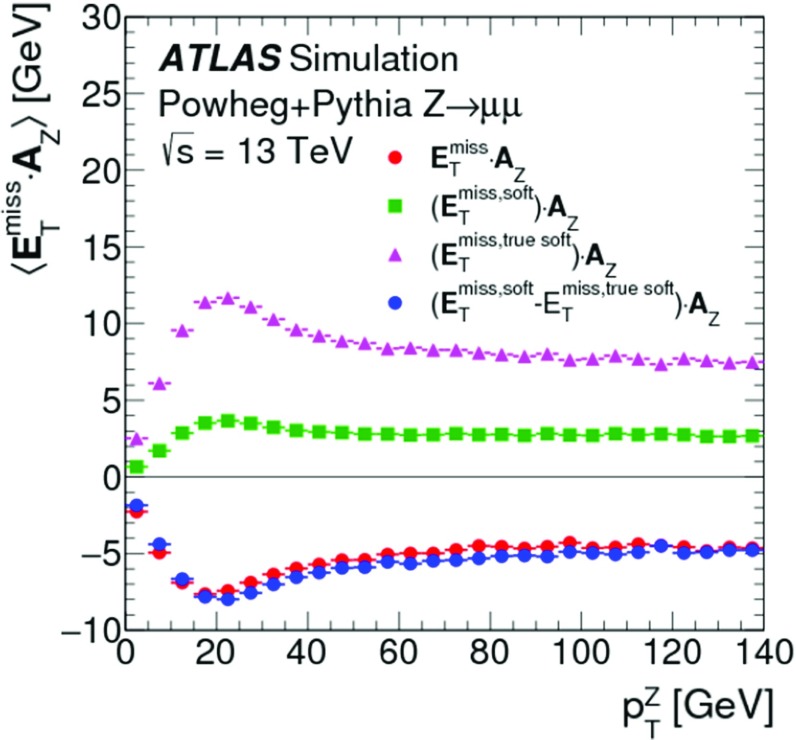
The average projection of $\mathbf E _{\mathrm{T}}^{{\text {miss}}}$ onto the direction $\mathbf A _{Z}$ of the $Z$ boson’s transverse momentum vector $\mathbf p _{\mathrm{T}}^{Z}$, as given in Eq. (10), is shown as a function of $p_{\mathrm{T}}^{Z} = |\mathbf p _{\mathrm{T}}^{Z} |$ in $Z \rightarrow \mu \mu$ events from the inclusive MC sample. The average projection of the soft term and the true soft term are also shown, to demonstrate the source of the deviation from zero

The persistent bias in $\langle \mathcal {P} _{\parallel }^{\,\!Z} \rangle$ is further explored in Fig. 5, which compares variations of $\langle \mathcal {P} _{\parallel }^{\,\!Z} \rangle$ respectively using the full $\mathbf E _{\mathrm{T}}^{{\text {miss}}}$, the soft-term contribution $\mathbf E _{\mathrm{T}}^{{\text {miss}},\text {soft}}$ only, the hard-term contribution $\mathbf E _{\mathrm{T}}^{{\text {miss}}}-\mathbf E _{\mathrm{T}}^{{\text {miss}},\text {soft}}$, and the true soft term $\mathbf E _{\mathrm{T}}^{{\text {miss}},\text {true soft}}$ only, as a function of $p_{\mathrm{T}}^{Z}$, for the $Z \rightarrow \mu \mu$ sample from MC simulations. In particular the difference between the projections using $\mathbf E _{\mathrm{T}}^{{\text {miss}},\text {true soft}}$ and $\mathbf E _{\mathrm{T}}^{{\text {miss}},\text {soft}}$ indicates the lack of reconstructed hadronic response, when $\mathbf E _{\mathrm{T}}^{{\text {miss}},\text {soft}} = \mathbf E _{\mathrm{T}}^{{\text {miss}},\text {true soft}}$ is expected for a fully measured recoil. The parallel projection using only the soft terms is larger than zero for all $p_{\mathrm{T}}^{Z}$ due to the missing $Z$-boson contribution to $\mathbf E _{\mathrm{T}}^{{\text {miss}}}$ given by $-\mathbf p _{\mathrm{T}}^{Z}$.

**Fig. 6:**
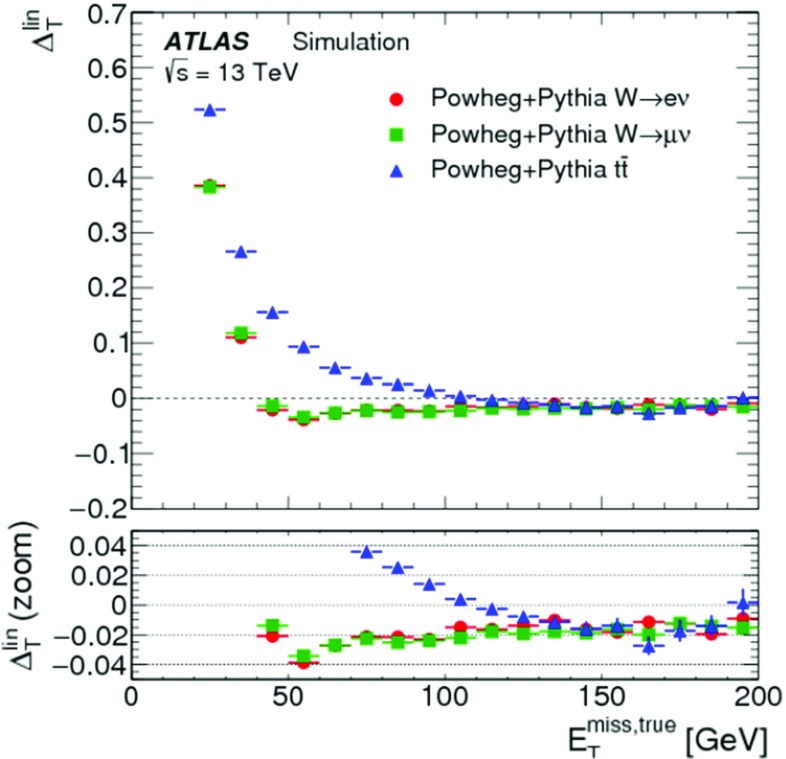
The deviation of the $E_{\mathrm{T}}^{\mathrm{miss}}$ response from linearity, measured as a function of the expected $E_{\mathrm{T}}^{{\text {miss}},\text {true}}$ by $\Delta _{\mathrm{T}}^{\mathrm{lin}}$ in Eq. (11), in $W \rightarrow e\nu$, $W \rightarrow \mu \nu$, and $t\bar{t}$ final states in MC simulations. The lower plot shows a zoomed-in view on the $\Delta _{\mathrm{T}}^{\mathrm{lin}}$ dependence on $E_{\mathrm{T}}^{{\text {miss}},\text {true}}$ with a highly suppressed ordinate

The deviation from linearity in $E_{\mathrm{T}}^{\mathrm{miss}}$ reconstruction, measured by $\Delta _{\mathrm{T}}^{\mathrm{lin}}$ given in Eq. (11), is shown as a function of $E_{\mathrm{T}}^{{\text {miss}},\text {true}}$ for MC simulations of $W \rightarrow e\nu$, $W \rightarrow \mu \nu$ and $t\bar{t}$ production in Fig. 6. The observed $\Delta _{\mathrm{T}}^{\mathrm{lin}} > 0$ at low $E_{\mathrm{T}}^{{\text {miss}},\text {true}}$ indicates an overestimation of $E_{\mathrm{T}}^{{\text {miss}},\text {true}}$ by the reconstructed $E_{\mathrm{T}}^{\mathrm{miss}}$ due to the observation biases arising from the finite $E_{\mathrm{T}}^{\mathrm{miss}}$ resolution, as discussed in Sect. 3.1. This bias overcompensates the lack of reconstructed $p_{\mathrm{T}}$-flow from the incompletely measured hadronic recoil in $W \rightarrow e\nu$ and $W \rightarrow \mu \nu$ events for $E_{\mathrm{T}}^{{\text {miss}},\text {true}} \lesssim 40\,{\text {Ge}\text {V}}$ with an increasing non-linearity observed with decreasing $E_{\mathrm{T}}^{{\text {miss}},\text {true}}$. For $E_{\mathrm{T}}^{{\text {miss}},\text {true}} \gtrsim 70\,{\text {Ge}\text {V}}$ the $E_{\mathrm{T}}^{\mathrm{miss}}$ response is directly proportional to $E_{\mathrm{T}}^{{\text {miss}},\text {true}}$, with the reconstructed recoil being approximately 2% too small. The $W \rightarrow e\nu$ and $W \rightarrow \mu \nu$ final states show very similar $\Delta _{\mathrm{T}}^{\mathrm{lin}} (E_{\mathrm{T}}^{{\text {miss}},\text {true}})$, thus indicating the universality of the recoil reconstruction and the independence on the lepton flavour of the reconstructed $E_{\mathrm{T}}^{\mathrm{miss}}$ in a low-multiplicity final state with $E_{\mathrm{T}}^{{\text {miss}},\text {true}} > 0$.

In $t\bar{t}$ final-state reconstruction, resolution effects tend to dominate $\Delta _{\mathrm{T}}^{\mathrm{lin}}$ at $E_{\mathrm{T}}^{{\text {miss}},\text {true}} \lesssim 120\,{\text {Ge}\text {V}}$. Compared to the $W \rightarrow e\nu$ and $W \rightarrow \mu \nu$ final states, a significantly poorer $E_{\mathrm{T}}^{\mathrm{miss}}$ resolution is observed in this kinematic region, due to the presence of at least four jets with relatively low $p_{\mathrm{T}}$ and high sensitivity to pile-up-induced fluctuations in each event of the $t\bar{t}$ sample. For $E_{\mathrm{T}}^{{\text {miss}},\text {true}} > 120\,{\text {Ge}\text {V}}$, $\Delta _{\mathrm{T}}^{\mathrm{lin}} (E_{\mathrm{T}}^{{\text {miss}},\text {true}}) \approx 2$% indicates a proportional $E_{\mathrm{T}}^{\mathrm{miss}}$ response with a systematic shift similar to the one observed in inclusive $W$-boson production.

#### Determination of the $E_{\mathrm{T}}^{\mathrm{miss}}$ resolution

The $E_{\mathrm{T}}^{\mathrm{miss}}$ resolution is determined by the width of the combined distribution of the differences between the measured $E_{x(y)}^{{\text {miss}}}$ and the components of the true missing transverse momentum vector $\mathbf E _{\mathrm{T}}^{{\text {miss}},\text {true}} = (E_{x}^{{\text {miss}},{\text {true}}},E_{y}^{{\text {miss}},{\text {true}}})$. The width is measured in terms of the ${\text {RMS}}$, with

12 $$\begin{aligned} \mathrm {RMS}^{\mathrm{miss}}_{x(y)} = \left\{ \begin{array}{ll} {\text {RMS}}(E_{x(y)}^{{\text {miss}}} - E_{x(y)}^{{\text {miss}},\text {true}}) &{} W \rightarrow e\nu \text { or } t\bar{t} \text { sample } (E_{\mathrm{T}}^{{\text {miss}},\text {true}} > 0) \\ {\text {RMS}}(E_{x(y)}^{{\text {miss}}}) &{} Z \rightarrow \mu \mu \text { sample } (E_{\mathrm{T}}^{{\text {miss}},\text {true}} = 0) \end{array}\right. . \end{aligned}$$

This metric does not capture all of the effects driving the fluctuations in $E_{\mathrm{T}}^{\mathrm{miss}}$ reconstruction, such as biases between individual $E_{\mathrm{T}}^{\mathrm{miss}}$ terms or the behaviour of outliers, but it is an appropriate general measure of how well $E_{\mathrm{T}}^{\mathrm{miss}}$ represents $E_{\mathrm{T}}^{{\text {miss}},\text {true}}$.

Using the $Z \rightarrow \mu \mu$ sample allows direct comparisons of $\mathrm {RMS}^{\mathrm{miss}}_{x(y)}$ between data and MC simulations, as $E_{\mathrm{T}}^{{\text {miss}},\text {true}} = 0$ in this case. The resolution in final states with genuine $E_{\mathrm{T}}^{\mathrm{miss}}$ is determined with MC simulations alone. For $W \rightarrow e\nu$ and $t\bar{t}$ final states, $E_{x(y)}^{{\text {miss}},\text {true}} = p_{x(y)}^{\nu }$ is used.

#### $E_{\mathrm{T}}^{\mathrm{miss}}$ resolution measurements

**Fig. 7:**
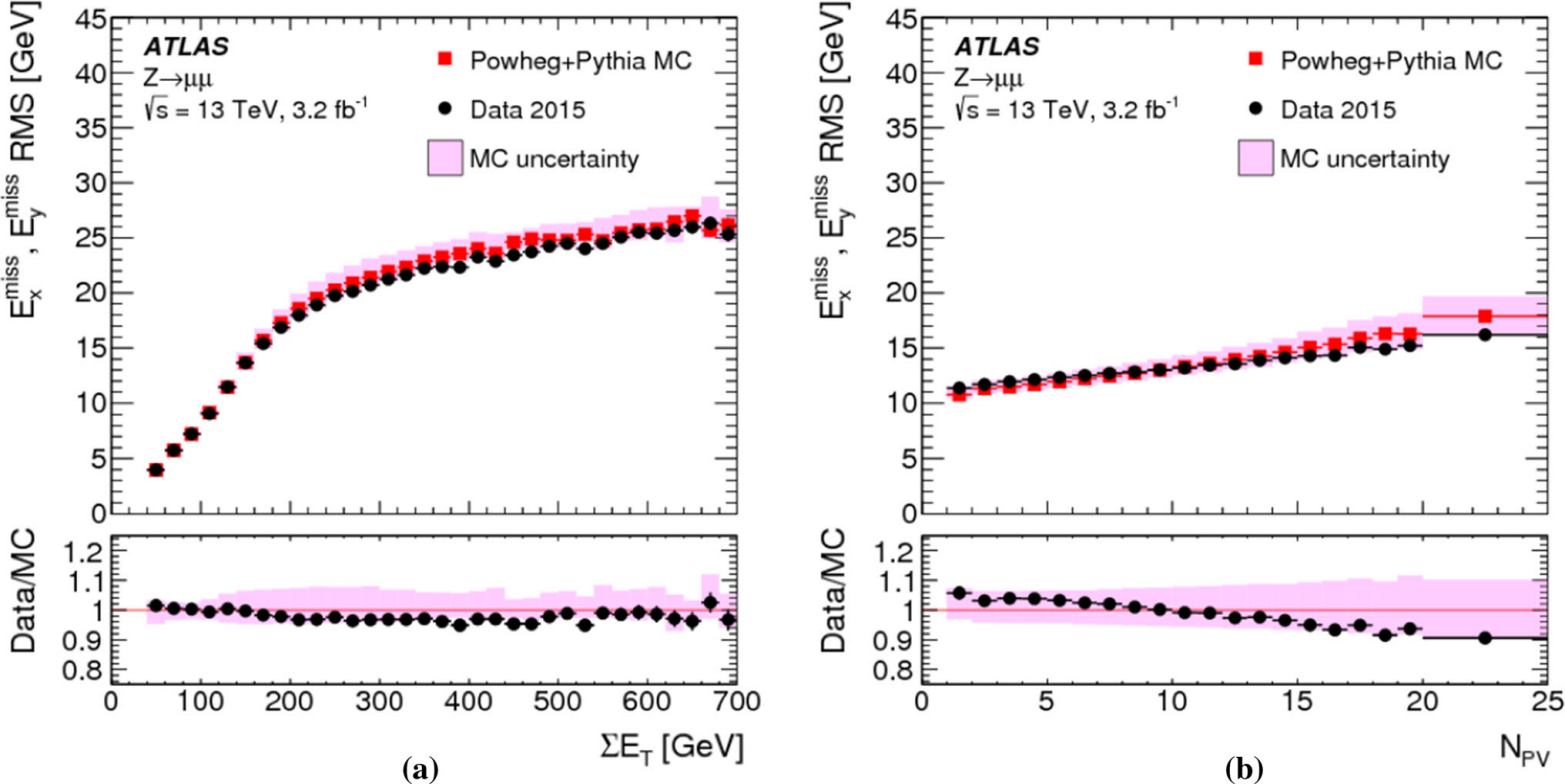
The RMS width of the $E_{x(y)}^{{\text {miss}}}$ distributions **a** in bins of $\Sigma E_{\mathrm{T}}$ and **b** in bins of the number of primary vertices in an inclusive sample of $Z \rightarrow \mu \mu$ events. Predictions from MC simulations are overlaid on the data points, and the ratios are shown below the respective plot. The shaded bands indicate the combined statistical and systematic uncertainties of the resolution measurements

**Fig. 8:**
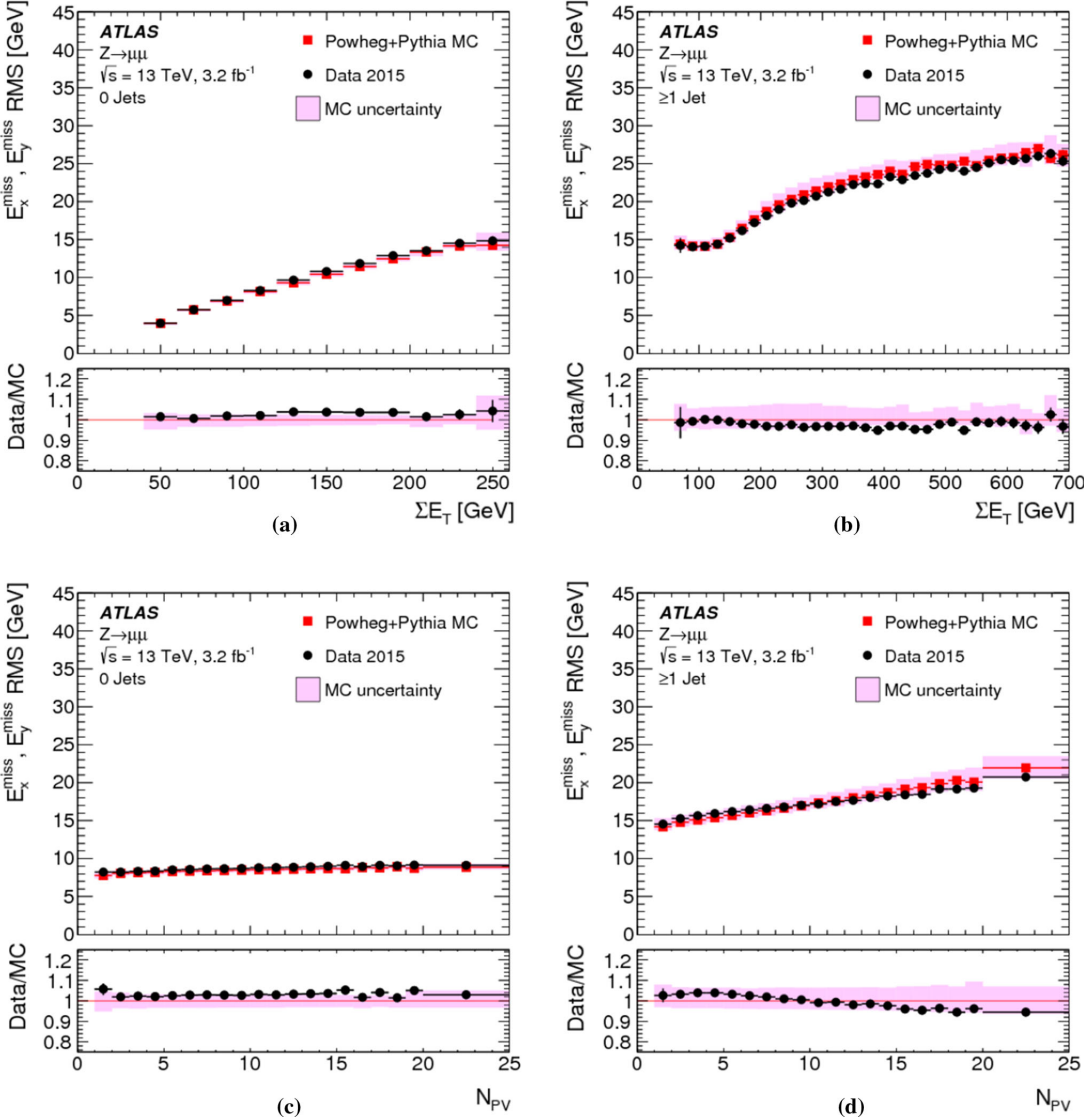
The $E_{\mathrm{T}}^{\mathrm{miss}}$ resolution $\mathrm {RMS}^{\mathrm{miss}}_{x(y)}$ determined for **a** an exclusive $Z \rightarrow \mu \mu$ sample without jets with $p_{\mathrm{T}} > 20\,{\text {Ge}\text {V}}$ ($N_{\mathrm{jet}} = 0$) and for **b** an exclusive sample with at least one jet above this threshold ($N_{\mathrm{jet}} \ge 1$), as a function of $\Sigma E_{\mathrm{T}}$ in data and MC simulations. The dependence of $\mathrm {RMS}^{\mathrm{miss}}_{x(y)}$ on the pile-up activity, as measured by $N_{\mathrm{PV}}$, for these two samples is shown in **c** and **d**, respectively. The shaded bands indicate the combined statistical and systematic uncertainties associated with the measurement

The $E_{\mathrm{T}}^{\mathrm{miss}}$ resolution measured by $\mathrm {RMS}^{\mathrm{miss}}_{x(y)}$ is evaluated as a function of the event activity measured by $\Sigma E_{\mathrm{T}}$ given in Eq. (7). For the inclusive $Z \rightarrow \mu \mu$ sample, Fig. 7a shows $\mathrm {RMS}^{\mathrm{miss}}_{x(y)}$ quickly rising from less than $5\,{\text {Ge}\text {V}}$ to about $10\,{\text {Ge}\text {V}}$ with increasing $\Sigma E_{\mathrm{T}}$ within $50\,{\text {Ge}\text {V}} \le \Sigma E_{\mathrm{T}} < 70\,{\text {Ge}\text {V}}$.^7^ This is due to the fact that in this range the two muons are the dominant hard objects contributing, with a $p_{\mathrm{T}}$ resolution proportional to $(p_{\mathrm{T}}^{\mu })^{2}$. A convolution of the muon resolution with a small contribution from $E_{\mathrm{T}}^{{\text {miss}},{\text {soft}}}$ is possible for $\Sigma E_{\mathrm{T}} > 50\,{\text {Ge}\text {V}}$. This component is on average about 60% of $p_{\mathrm{T}}^{Z}$, and subject to the stochastic fluctuations further discussed below.

The increase of $Z \rightarrow \mu \mu + 1$ jet topologies in the $Z \rightarrow \mu \mu$ sample leads to an additional source of fluctuations affecting $\mathrm {RMS}^{\mathrm{miss}}_{x(y)} (\Sigma E_{\mathrm{T}})$ for $70\,{\text {Ge}\text {V}} < \Sigma E_{\mathrm{T}} \lesssim 180\,{\text {Ge}\text {V}}$. In general the $Z \rightarrow \mu \mu$ sample collected for this study covers $p_{\mathrm{T}}^{Z} \lesssim 140\,{\text {Ge}\text {V}}$ with relevant statistics. At this limit it is expected that the hadronic recoil contains two reconstructed jets, with the onset of this contribution at $\Sigma E_{\mathrm{T}}$ of about $180\,{\text {Ge}\text {V}}$. The corresponding change of the dominant final state composition for $\Sigma E_{\mathrm{T}} > 180\,{\text {Ge}\text {V}}$ leads to a change of shape of $\mathrm {RMS}^{\mathrm{miss}}_{x(y)} (\Sigma E_{\mathrm{T}})$, as the transverse momentum of the individual jets rises and the number of contributing jets slowly increases. The expected $\mathrm {RMS}^{\mathrm{miss}}_{x(y)} (\Sigma E_{\mathrm{T}}) \propto \sqrt{\Sigma E_{\mathrm{T}}}$ scaling driven by the jet-$p_{\mathrm{T}}$ resolution [44] therefore dominates $\mathrm {RMS}^{\mathrm{miss}}_{x(y)}$ at these higher $\Sigma E_{\mathrm{T}}$. The MC predictions for $\mathrm {RMS}^{\mathrm{miss}}_{x(y)} (\Sigma E_{\mathrm{T}})$ agree with the data within a few percent and well within the total uncertainties of this measurement. A tendency for slightly poorer resolution in MC simulations is observed, in particular for $\Sigma E_{\mathrm{T}} > 200\,{\text {Ge}\text {V}}$.

Any contribution from pile-up to $\mathrm {RMS}^{\mathrm{miss}}_{x(y)}$ is expected to be associated with the jets. While dedicated corrections applied to the jets largely suppress pile-up contributions in the jet response, residual irreducible fluctuations introduced into the calorimeter signals by pile-up lead to a degradation of the jet energy resolution and thus poorer resolution in the jet-$p_{\mathrm{T}}$ measurement. The dependence of $\mathrm {RMS}^{\mathrm{miss}}_{x(y)}$ on the pile-up activity measured by $N_{\mathrm{PV}}$ is shown in Fig. 7b. Data show a less steep slope of $\mathrm {RMS}^{\mathrm{miss}}_{x(y)} (N_{\mathrm{PV}})$ than MC simulations, but with about 10% worse resolution in the low pile-up region of $N_{\mathrm{PV}} \lesssim 5$. The resolution in data is better than in MC simulations by about 10% for the region of higher pile-up activity at $N_{\mathrm{PV}} \approx 20$.

The differences between data and MC simulations seen in $\mathrm {RMS}^{\mathrm{miss}}_{x(y)} (\Sigma E_{\mathrm{T}})$ for the inclusive $Z \rightarrow \mu \mu$ sample can be further analysed by splitting the sample according to the value of $N_{\mathrm{jet}}$. Figure 8a shows the dependence of $\mathrm {RMS}^{\mathrm{miss}}_{x(y)}$ on $\Sigma E_{\mathrm{T}}$ for $Z \rightarrow \mu \mu$ events with $N_{\mathrm{jet}} = 0$. The dominant source of fluctuations other than the muon-$p_{\mathrm{T}}$ resolution is in this case introduced by the incomplete reconstruction of the hadronic recoil. These fluctuations increase with increasing $p_{\mathrm{T}}^{Z}$, which in turn means higher overall event activity measured by $\Sigma E_{\mathrm{T}}$. For this sample $\mathrm {RMS}^{\mathrm{miss}}_{x(y)}$ in data compares well to MC simulations, at a level of a few percent, without any observed dependence on $\Sigma E_{\mathrm{T}}$.

The exclusive $N_{\mathrm{jet}} \ge 1$ samples extracted from $Z \rightarrow \mu \mu$ data and MC simulations show the expected $\mathrm {RMS}^{\mathrm{miss}}_{x(y)} \propto \sqrt{\Sigma E_{\mathrm{T}}}$ scaling in Fig. 8b. The resolution in data is well represented by MC simulations, at the level of a few percent. The slightly better resolution observed in data with increasing $\Sigma E_{\mathrm{T}}$ follows the trend observed in Fig. 7a. The similar trends are expected as this kinematic region is largely affected by the jet contribution.

The dependence of $\mathrm {RMS}^{\mathrm{miss}}_{x(y)}$ on $N_{\mathrm{PV}}$ shown in Fig. 8c indicates that the $E_{\mathrm{T}}^{\mathrm{miss}}$ resolution is basically independent of pile-up, for the $N_{\mathrm{jet}} = 0$ sample. This is expected from the exclusive $E_{\mathrm{T}}^{\mathrm{miss}}$ composition comprising the (track-based) $E_{\mathrm{T}}^{{\text {miss}},\mu }$ and $E_{\mathrm{T}}^{{\text {miss}},{\text {soft}}}$ terms only. Data and MC simulations compare well within a few percent, and without any observable dependence on $N_{\mathrm{PV}}$. Figure 8d shows the $N_{\mathrm{PV}}$ dependence of $\mathrm {RMS}^{\mathrm{miss}}_{x(y)}$ for the $N_{\mathrm{jet}} \ge 1$ sample. Comparing this result to Fig. 7b confirms that all pile-up dependence of the $E_{\mathrm{T}}^{\mathrm{miss}}$ resolution is arising from the jet term. Both trend and magnitude of the data-to-MC comparison follow the observation from the inclusive analysis.

#### $E_{\mathrm{T}}^{\mathrm{miss}}$ resolution in final states with neutrinos

**Fig. 9:**
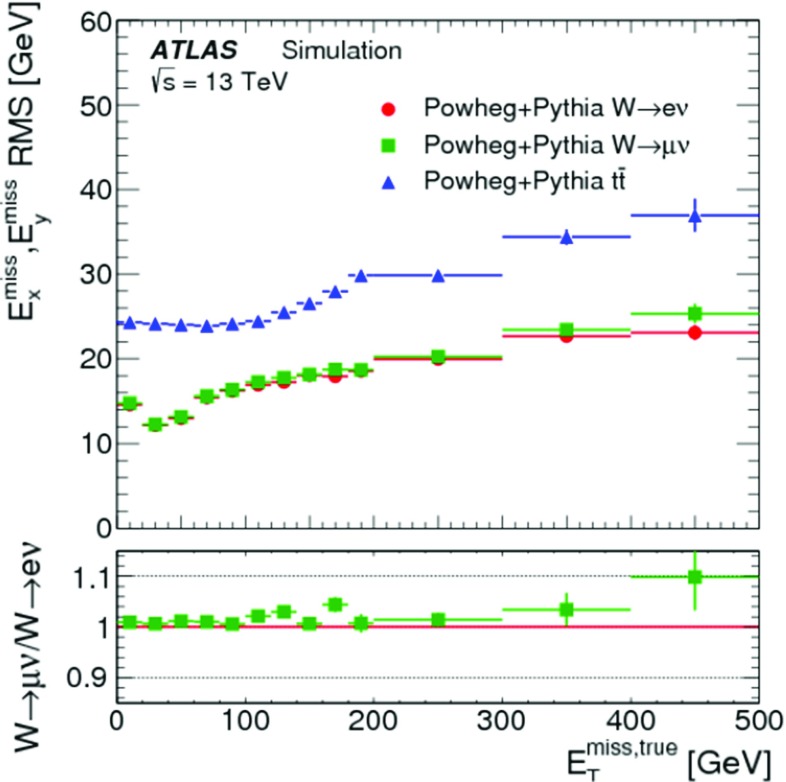
The $E_{\mathrm{T}}^{\mathrm{miss}}$ resolution measured by $\mathrm {RMS}^{\mathrm{miss}}_{x(y)}$ as a function of the true missing transverse momentum $E_{\mathrm{T}}^{{\text {miss}},\text {true}}$ for the $W \rightarrow e\nu$, $W \rightarrow \mu \nu$, and $t\bar{t}$ samples from MC simulations

The $E_{\mathrm{T}}^{\mathrm{miss}}$ resolution for final states with $E_{\mathrm{T}}^{{\text {miss}},\text {true}} > 0$ is measured by $\mathrm {RMS}^{\mathrm{miss}}_{x(y)}$ according to Eq. (12) and evaluated using dedicated inclusive $W \rightarrow e\nu$ and $W \rightarrow \mu \nu$ samples from MC simulations, and the inclusive $t\bar{t}$ MC sample defined in Sect. 5.3. For these samples, $\mathrm {RMS}^{\mathrm{miss}}_{x(y)}$ can be determined as a function of $E_{\mathrm{T}}^{{\text {miss}},\text {true}} = p_{\mathrm{T}}^{\nu }$. The dedicated $W \rightarrow e\nu$ and $W \rightarrow \mu \nu$ samples are obtained with an event selection based on the description in Sect. 5.2, but omitting both the $E_{\mathrm{T}}^{\mathrm{miss}}$-based and the $m_{\mathrm{T}}$-based selections.

Figure 9 shows $\mathrm {RMS}^{\mathrm{miss}}_{x(y)}$ evaluated as a function of $E_{\mathrm{T}}^{{\text {miss}},\text {true}}$ for these samples. The universality of the response to the hadronic recoil observed in Fig. 6, together with the different but subdominant contributions from the $p_{\mathrm{T}}$ resolutions of the electrons and muons, yield a very similar $E_{\mathrm{T}}^{\mathrm{miss}}$ resolution for $W \rightarrow e\nu$ and $W \rightarrow \mu \nu$ final states. Generally, poorer resolution is observed in $t\bar{t}$ final states. The deviation from the expected $\mathrm {RMS}^{\mathrm{miss}}_{x(y)} \propto \sqrt{(E_{\mathrm{T}}^{{\text {miss}},\text {true}})}$ scaling behaviour for $W \rightarrow \ell \nu$ at lower $E_{\mathrm{T}}^{{\text {miss}},\text {true}}$ reflects the kinematic features of the $W$ boson and its decay. Events with low $p_{\mathrm{T}}^{W}$, and therefore small hadronic recoil, lie predominantly in the region $25\,{\text {Ge}\text {V}} \lesssim p_{\mathrm{T}}^{\nu } \lesssim 50\,{\text {Ge}\text {V}}$. Since the hadronic recoil is generally the poorly measured component of an event and the reconstructed $E_{\mathrm{T}}^{\mathrm{miss}}$ is dominated by the lepton $p_{\mathrm{T}}$ in this region, the $E_{\mathrm{T}}^{\mathrm{miss}}$ resolution tends to be better here than for events with larger hadronic recoil populating $p_{\mathrm{T}}^{\nu } \lesssim 25\,{\text {Ge}\text {V}}$ and $p_{\mathrm{T}}^{\nu } \gtrsim 50\,{\text {Ge}\text {V}}$.

### $E_{\mathrm{T}}^{\mathrm{miss}}$ tails

Large reconstructed $E_{\mathrm{T}}^{\mathrm{miss}}$ is an indicator for the production of (potentially new) undetectable particles, but can also be generated by detector problems and/or poor reconstruction of the objects used for its reconstruction. Enhanced tails in the distribution of the $\mathbf E _{\mathrm{T}}^{{\text {miss}}}$ components for final states with well-known expectation values for $E_{\mathrm{T}}^{\mathrm{miss}}$ are indicative of such inefficiencies.

Non-Gaussian shapes in the distribution arise from a combination of object selection inefficiencies and potentially non-Gaussian resolutions of the $E_{\mathrm{T}}^{\mathrm{miss}}$ constituents. Even for a well-defined final state, event-by-event fluctuations in terms of which particles, jets, and soft tracks enter the $E_{\mathrm{T}}^{\mathrm{miss}}$ reconstruction, and with which $p_{\mathrm{T}}$, lead to deviations from a normally distributed ($E_{x}^{{\text {miss}}}$,$E_{y}^{{\text {miss}}}$) response.

**Fig. 10:**
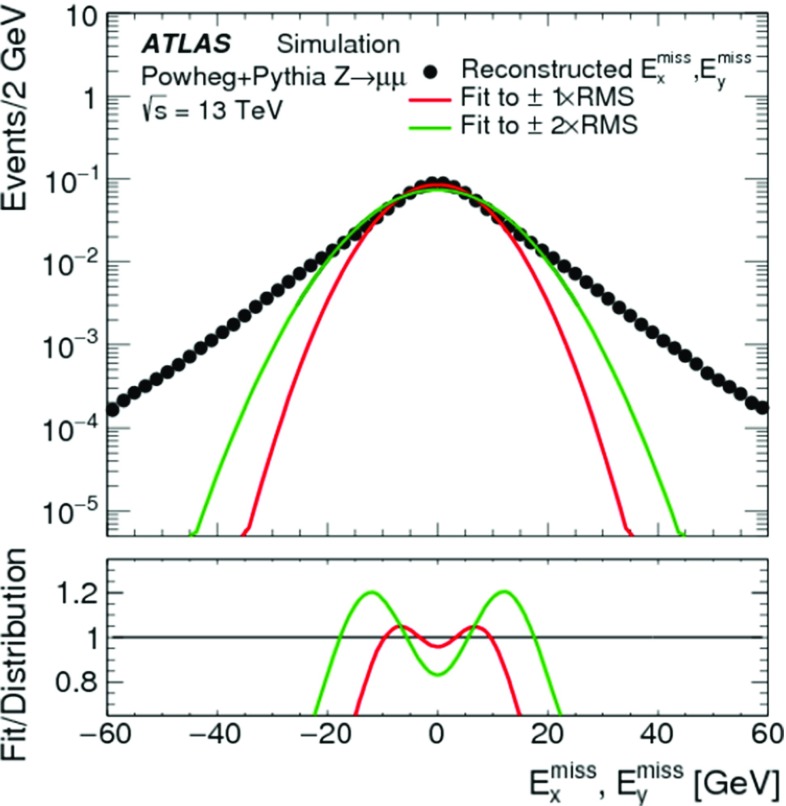
The combined distribution of $E_{x}^{{\text {miss}}}$ and $E_{y}^{{\text {miss}}}$ for an inclusive $Z \rightarrow \mu \mu$ from simulation. Gaussian fits limited to the $\pm 1\times \text {RMS}$ and $\pm 2\times \text {RMS}$ ranges around the centre of the distribution are shown, together with the respective differences between the fitted functions and the actual distribution

Figure 10 shows the combined ($E_{x}^{{\text {miss}}}$,$E_{y}^{{\text {miss}}}$) distribution for the inclusive $Z \rightarrow \mu \mu$ sample from MC simulations. To illustrate its symmetric nature and its deviation from a normal distribution in particular with respect to the tails, Gaussian functions are fitted to two limited ranges around the centre of the distribution, $\pm 1\times \text {RMS}$ and $\pm 2\times \text {RMS}$. The differences between these functions and the data distribution (lower panel of Fig. 10) indicate a more peaked shape around the most probable value for $E_{x(y)}^{{\text {miss}}}$ with near exponential slopes. The result of this comparison supports the choice of $\mathrm {RMS}^{\mathrm{miss}}_{x(y)}$ defined in Eq. (12) in Sect. 6.2.3 for the determination of the $E_{\mathrm{T}}^{\mathrm{miss}}$ resolution, rather than using any of the widths measured by fitting Gauss functions in selected ranges of the distribution.

The tails in this shape are reflected in the distribution of $E_{\mathrm{T}}^{\mathrm{miss}}$ itself and can be estimated by measuring the fraction of events with $E_{\mathrm{T}}^{\mathrm{miss}} > E_{\mathrm{T}}^{{\text {miss}},\text {threshold}}$,

13 $$\begin{aligned}&f_{\mathrm{tail}} = \dfrac{1}{\mathcal {H}}{\int _{E_{\mathrm{T}}^{{\text {miss}},\text {threshold}}}^{\infty } h(E_{\mathrm{T}}^{\mathrm{miss}}) \text {d}E_{\mathrm{T}}^{\mathrm{miss}}}, \nonumber \\&\quad \text {with }\mathcal {H} = \int _{0}^{\infty } h(E_{\mathrm{T}}^{\mathrm{miss}}) \text {d}E_{\mathrm{T}}^{\mathrm{miss}}. \end{aligned}$$

Here $h(E_{\mathrm{T}}^{\mathrm{miss}})$ is the $E_{\mathrm{T}}^{\mathrm{miss}}$ distribution for a given event sample, and $E_{\mathrm{T}}^{{\text {miss}},\text {threshold}}$ is a threshold set to estimate tails. Any decrease of $f_{\mathrm{tail}}$ at a fixed integral $\mathcal {H}$ indicates an improvement of the $E_{\mathrm{T}}^{\mathrm{miss}}$ resolution, and is more sensitive to particular improvements than e.g. $\mathrm {RMS}^{\mathrm{miss}}_{x(y)}$. For example, improving the $E_{\mathrm{T}}^{{\text {miss}},{\text {soft}}}$ reconstruction by rejecting ID tracks from the hard-scatter vertex with poor reconstruction quality yields a significantly smaller $f_{\mathrm{tail}}$ for the same event sample.

**Fig. 11:**
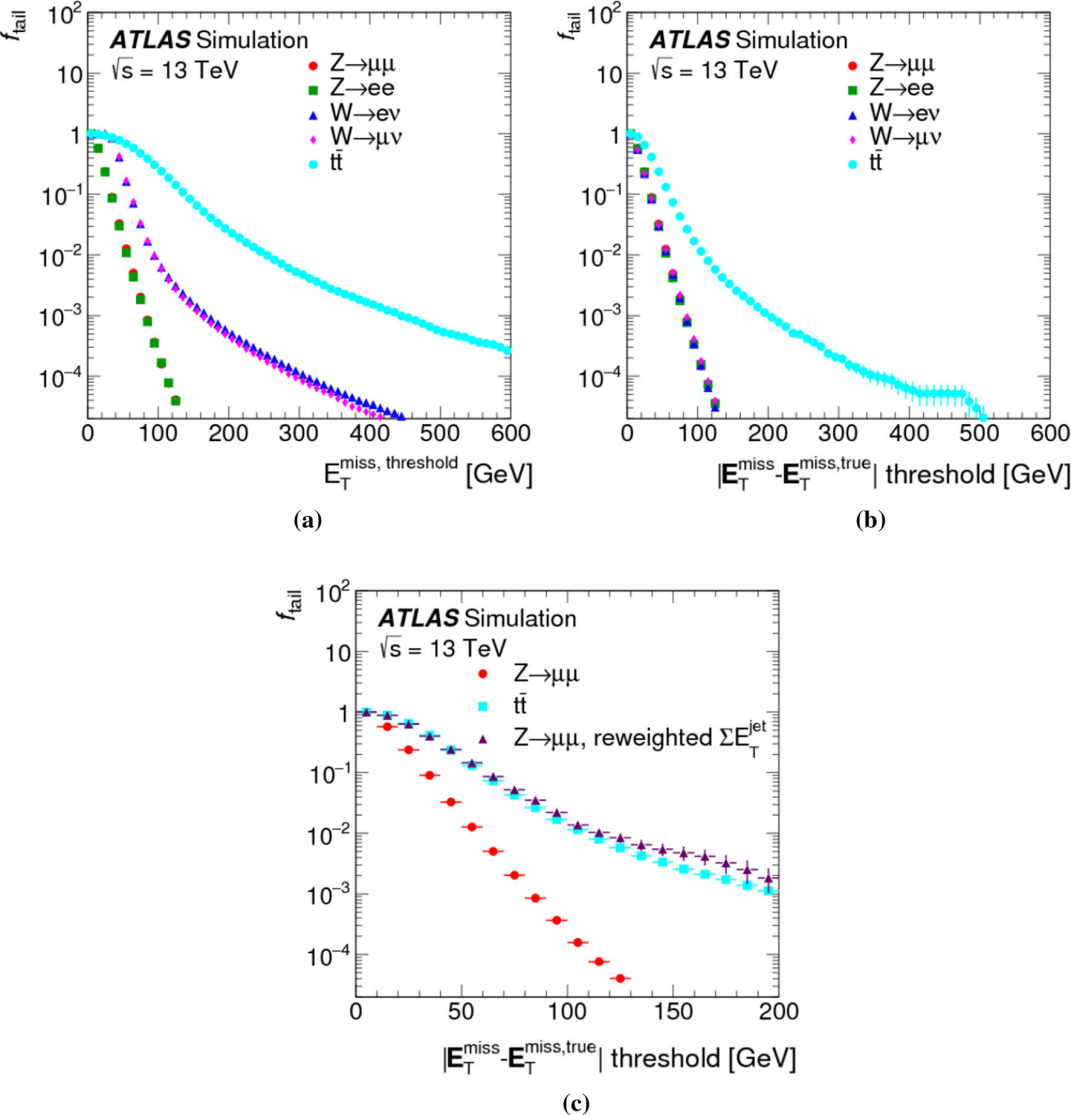
In **a** the integral tail fraction $f_{\mathrm{tail}}$ given in Eq. (13) is shown as a function of the integration threshold $E_{\mathrm{T}}^{{\text {miss}},\text {threshold}}$, for MC simulations of $Z \rightarrow \ell \ell$, $W \rightarrow \ell \nu$, and $t\bar{t}$ final states. The tail fraction in terms of a threshold applied to $|\mathbf E _{\mathrm{T}}^{{\text {miss}}}-\mathbf E _{\mathrm{T}}^{{\text {miss}},\text {true}} |$, the distance between the reconstructed ($\mathbf E _{\mathrm{T}}^{{\text {miss}}}$) and the expected ($\mathbf E _{\mathrm{T}}^{{\text {miss}},\text {true}}$ ) vectors, is shown in **b** for all considered final states. The same fraction is shown in **c** for the $E_{\mathrm{T}}^{\mathrm{miss}}$ distributions for $Z \rightarrow \mu \mu$ before and after a reweighting following the $\Sigma E_{\mathrm{T}}$ distribution for $t\bar{t}$ is applied, together with $f_{\mathrm{tail}}$ from the $t\bar{t}$ final state

The tails in the $E_{\mathrm{T}}^{\mathrm{miss}}$ distributions for the final states considered for this study are quantified by the fraction of events above a certain $E_{\mathrm{T}}^{\mathrm{miss}}$ threshold using MC simulations. Figure 11a shows that the $Z \rightarrow \ell \ell$ events ($\ell = e$ or $\ell = \mu$) with $E_{\mathrm{T}}^{{\text {miss}},\text {true}} = 0$ have significantly reduced tails when compared to $W \rightarrow \ell \nu$ and $t\bar{t}$ with this metric, and that the tails do not depend on the lepton flavour. A modification of this metric, taking into account $E_{\mathrm{T}}^{{\text {miss}},\text {true}}$ such that the fraction of events with $|\mathbf E _{\mathrm{T}}^{{\text {miss}}}- \mathbf E _{\mathrm{T}}^{{\text {miss}},\text {true}} |$ above a given threshold is determined, shows the universality of the hadronic recoil in $Z \rightarrow \ell \ell$ and $W \rightarrow \ell \nu$, as can be seen in Fig. 11b.

Another finding of this study is that the tail in the $$|\mathbf E _{\mathrm{T}}^{{\text {miss}}}-\mathbf E _{\mathrm{T}}^{{\text {miss}},\text {true}} |$$ distribution for the higher $$\Sigma E_{\mathrm{T}}^{\text {jet}}$$$$t\bar{t}$$ sample is considerably larger than for the low-$$\Sigma E_{\mathrm{T}}^{\text {jet}}$$ samples with $$Z \rightarrow \ell \ell $$ or $$W \rightarrow \ell \nu $$ final states. As can be seen in Fig. 11c, the tails are much more consistent between $$Z \rightarrow \mu \mu $$ and $$t\bar{t}$$ samples when the distribution for the $$Z \rightarrow \mu \mu $$ sample is reweighted such that it follows the same $$\Sigma E_{\mathrm{T}}^{\text {jet}}$$ distribution as the $$t\bar{t}$$ sample. The enhanced tails are thus likely introduced by the jet response and multiplicity, which has a residual sensitivity to pile-up.

## Systematic uncertainties

The systematic uncertainties associated with the measurement of $E_{\mathrm{T}}^{\mathrm{miss}}$ are provided for the response ($E_{\mathrm{T}}^{\mathrm{miss}}$ scale) as well as for the resolution. They depend on the composition of the hard terms and on the magnitude of the corresponding soft term. As the hard-term composition is generally defined by optimisations implemented in the context of a given analysis, the contributions of the $E_{\mathrm{T}}^{\mathrm{miss}}$ terms need to be extracted from the scale and resolution uncertainties for the individual contributing objects comprising electrons, photons, muons, $\tau \text {-leptons}$, and jets. In the corresponding propagations, correlations between systematic uncertainties for the same type of object are typically taken into account. However, it is assumed that systematic uncertainties of the different object types entering $E_{\mathrm{T}}^{\mathrm{miss}}$ reconstruction are uncorrelated. The determination of the $E_{\mathrm{T}}^{\mathrm{miss}}$ scale and resolution uncertainties arising from the soft term $E_{\mathrm{T}}^{{\text {miss}},{\text {soft}}}$ is described in this section.

### Methodology

The extraction of the systematic uncertainties for the reconstructed $E_{\mathrm{T}}^{\mathrm{miss}}$ is based on data-to-MC comparisons of spectra of observables measuring the contribution of $E_{\mathrm{T}}^{{\text {miss}},{\text {soft}}}$ to the overall $E_{\mathrm{T}}^{\mathrm{miss}}$.

#### Observables

The vector sum of the transverse momentum vectors of all particles and jets emerging from a hard-scatter interaction ($\mathbf p _{\mathrm{T}}^{\text {HS}}$) is given byHere $\mathbf p _{\mathrm{T}}^{\nu }$ generally represents the transverse momenta of non-observable particles, which are summed up to form $\mathbf p _{\mathrm{T}}^{\text {inv}}$. All other transverse momenta are carried by particles that are observable in principle, and sum up to $\mathbf p _{\mathrm{T}}^{\text {obs}}$. Momentum conservation dictates $p_{\mathrm{T}}^{\text {HS}} = |\mathbf p _{\mathrm{T}}^{\text {HS}} | = 0$.

Due to detector acceptance limitations and inefficiencies in hard-object reconstruction and calibration, and all other effects discussed in Sect. 3, only a proxy ($\mathbf p _{\mathrm{T}}^{{\text {hard}}}$) for the observable-particle contribution $\mathbf p _{\mathrm{T}}^{\text {obs}}$ can be measured. The reconstructed hard final-state objects entering $E_{\mathrm{T}}^{\mathrm{miss}}$ as described in Sect. 3.2 are used to measure $\mathbf p _{\mathrm{T}}^{{\text {hard}}}$ as

$$\begin{aligned} \mathbf p _{\mathrm{T}}^{{\text {hard}}}= & {} \sum _{\begin{array}{c} \text {contributing} \\ \text {electrons} \end{array}} \mathbf p _{\mathrm{T}}^{e} + \sum _{\begin{array}{c} \text {contributing} \\ \text {photons} \end{array}} \mathbf p _{\mathrm{T}}^{\gamma } + \sum _{\begin{array}{c} \text {contributing} \\ \tau \text {-leptons} \end{array}} \mathbf p _{\mathrm{T}}^{\tau _{\mathrm{had}}} \\&+\, \sum _{\begin{array}{c} \text {contributing} \\ \text {muons} \end{array}} \mathbf p _{\mathrm{T}}^{\mu } + \sum _{\begin{array}{c} \text {contributing} \\ \text {jets} \end{array}} \mathbf p _{\mathrm{T}}^{\text {jet}}. \end{aligned}$$

The expectation is that $p_{\mathrm{T}}^{{\text {hard}}} = |\mathbf p _{\mathrm{T}}^{{\text {hard}}} | > 0$ and $\mathbf p _{\mathrm{T}}^{{\text {hard}}} \ne \mathbf p _{\mathrm{T}}^{\text {obs}}$. Adding $\mathbf p _{\mathrm{T}}^{{\text {soft}}} = -\mathbf E _{\mathrm{T}}^{{\text {miss}},\text {soft}}$, with $\mathbf E _{\mathrm{T}}^{{\text {miss}},\text {soft}}$ defined in Eq. (6), to $\mathbf p _{\mathrm{T}}^{{\text {hard}}}$ yields an improved estimate of the net transverse momentum carried by undetectable particles, as some of the experimental inefficiencies are mitigated.^8^

**Fig. 12:**
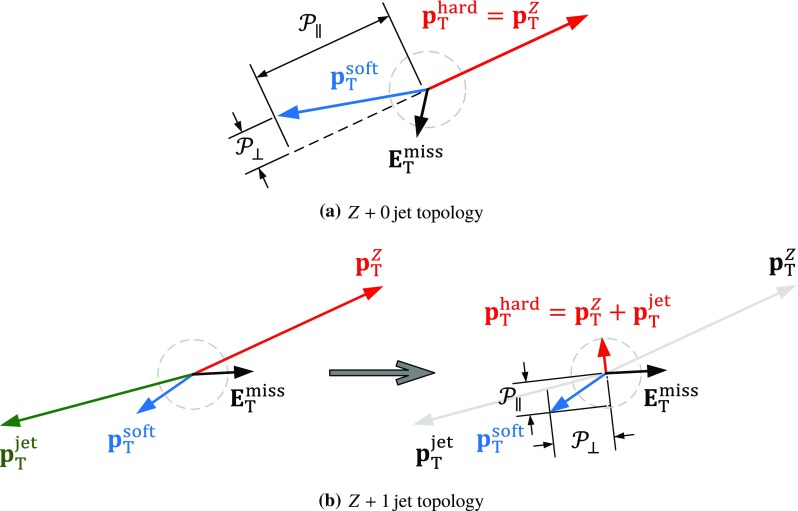
Schematic view of the parallel ($\mathcal {P} _{\parallel }$) and perpendicular ($\mathcal {P} _{\!\perp }$) projections of $\mathbf p _{\mathrm{T}}^{{\text {soft}}}$ on $\mathbf p _{\mathrm{T}}^{{\text {hard}}}$ for $Z \rightarrow \mu \mu$ events without genuine $E_{\mathrm{T}}^{\mathrm{miss}}$, for **a** a final state without any jets and **b** a final state with one jet. The expectation values for a perfect $E_{\mathrm{T}}^{\mathrm{miss}}$ reconstruction are $\text {E}[\mathcal {P} _{\parallel } ] = p_{\mathrm{T}}^{Z}$ for $N_{\mathrm{jet}} = 0$ and $\text {E}[\mathcal {P} _{\parallel } ] = p_{\mathrm{T}}^{{\text {hard}}}$ for $N_{\mathrm{jet}} \ge 1$, with $\text {E}[\mathcal {P} _{\!\perp } ] = 0$ in all cases. **a** $Z + 0\,\text {jet}$ topology. **b** $Z + 1\,\text {jet}$ topology

In the $Z \rightarrow \mu \mu$ final state without genuine missing transverse momentum the expectation is that $\mathbf E _{\mathrm{T}}^{{\text {miss}}} = -(\mathbf p _{\mathrm{T}}^{{\text {hard}}} + \mathbf p _{\mathrm{T}}^{{\text {soft}}}) = \mathbf {0}$. While this expectation does not hold due to the experimental inefficiencies, it nevertheless raises the expectation that for events without jets $\mathbf p _{\mathrm{T}}^{{\text {soft}}}$ points into the direction of the hadronic recoil, i.e. opposite to $\mathbf p _{\mathrm{T}}^{{\text {hard}}}$ in the transverse-momentum plane. The deviation from this expectation is measured in terms of the parallel ($\mathcal {P} _{\parallel }$) and perpendicular ($\mathcal {P} _{\!\perp }$) projections of $\mathbf p _{\mathrm{T}}^{{\text {soft}}}$ onto $\mathbf p _{\mathrm{T}}^{{\text {hard}}}$. Figure 12 schematically shows these projections for $Z + 0$-jet and $Z + 1$-jet topologies.

The average $\langle \mathcal {P} _{\parallel } \rangle$ in a given bin *k* of phase space defined by $p_{\mathrm{T}}^{{\text {hard}}}$ measures the $E_{\mathrm{T}}^{{\text {miss}},{\text {soft}}}$ response, with $\langle \mathcal {P} _{\parallel } \rangle = \langle p_{\mathrm{T}}^{{\text {hard}}} \rangle _{k}$ indicating a perfect response in this bin. The $E_{\mathrm{T}}^{\mathrm{miss}}$ resolution contribution from $E_{\mathrm{T}}^{{\text {miss}},{\text {soft}}}$ reconstruction is measured by two components, the fluctuations in response ($\mathrm {RMS}^{2}_{\!\parallel }$) and the fluctuations of the (transverse) angular deflection around the $\mathbf p _{\mathrm{T}}^{{\text {hard}}}$ axis, measured by $\mathrm {RMS}^{2}_{\!\perp }$. These fluctuations are expressed in terms of variances, with

$$\begin{aligned} \mathrm {RMS}^{2}_{\!\parallel } = \left\langle (\mathcal {P} _{\parallel })^{2}\right\rangle - \left\langle \mathcal {P} _{\parallel } \right\rangle ^{2} \quad \text {and}\quad \mathrm {RMS}^{2}_{\!\perp } = \left\langle (\mathcal {P} _{\!\perp })^{2}\right\rangle . \end{aligned}$$

#### Procedures

The extraction of the systematic uncertainties introduced into the $E_{\mathrm{T}}^{\mathrm{miss}}$ measurement by the $E_{\mathrm{T}}^{{\text {miss}},{\text {soft}}}$ term is based on data-to-MC-simulations comparisons of $\langle \mathcal {P} _{\parallel } \rangle (p_{\mathrm{T}}^{{\text {hard}}})$ for the response, and of $\mathrm {RMS}^{2}_{\!\parallel } (p_{\mathrm{T}}^{{\text {hard}}})$ and $\mathrm {RMS}^{2}_{\!\perp } (p_{\mathrm{T}}^{{\text {hard}}})$ for the resolution. Alternative MC samples are considered, with variations of either the event generator or the detector simulation (description and shower models). For the highest impact of $E_{\mathrm{T}}^{{\text {miss}},{\text {soft}}}$ on $E_{\mathrm{T}}^{\mathrm{miss}}$, the exclusive $Z \rightarrow \mu \mu$ selection with $N_{\mathrm{jet}} = 0$ is the basis for the determination of the systematic uncertainty components for both data and all MC simulations. In this case, the only hard contribution is from the reconstructed $Z$ boson, i.e. $\mathbf p _{\mathrm{T}}^{{\text {hard}}} = \mathbf p _{\mathrm{T}}^{Z}$ as shown in Fig. 12a.

**Fig. 13:**
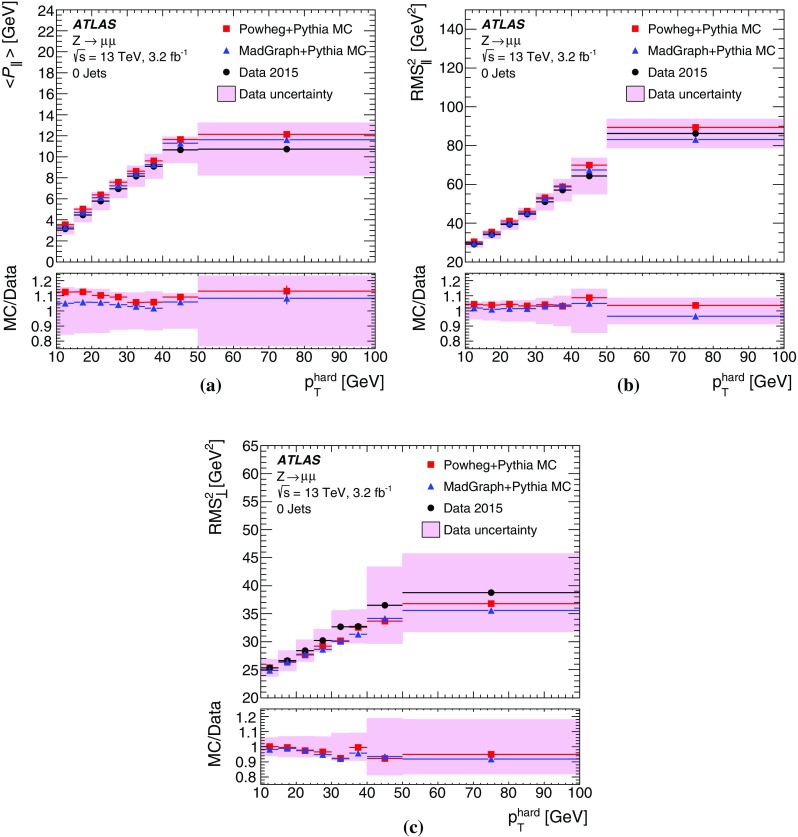
The **a** average value of the longitudinal projection $\langle \mathcal {P} _{\parallel } \rangle$ and the **b** variance $\mathrm {RMS}^{2}_{\!\parallel }$ of the longitudinal projection $\mathcal {P} _{\parallel }$ of $\mathbf p _{\mathrm{T}}^{{\text {soft}}}$ onto $\mathbf p _{\mathrm{T}}^{{\text {hard}}}$ for $Z \rightarrow \mu \mu$ event with $N_{\mathrm{jet}} = 0$, for data and two different MC simulations, shown as a function of $p_{\mathrm{T}}^{{\text {hard}}}$. The variance $\mathrm {RMS}^{2}_{\!\perp }$ of the perpendicular projection $\mathcal {P} _{\!\perp }$ is shown in **c** for the same event samples. The shaded band indicates the systematic uncertainties derived as described in the text

The uncertainties are determined by comparing $\mathcal {P} _{\parallel }$ and $\mathcal {P} _{\!\perp }$ spectra from data and MC simulations, in bins of $p_{\mathrm{T}}^{{\text {hard}}}$. For $\mathcal {P} _{\parallel }$, the smearing of the response and the width both yield scale and (longitudinal) resolution offsets. In the case of $\mathcal {P} _{\!\perp }$, only smearing of the width is applied to provide transverse resolution offsets. These fitted offsets, determined for the various MC configurations, provide the systematic uncertainties with respect to a specific MC modelling configuration. In practice, to account for the resolution offsets, Gaussian smearing is applied in simulation to the longitudinal and transverse components of $\mathbf E _{\mathrm{T}}^{{\text {miss}},\text {soft}}$ relative to the direction of $\mathbf p _{\mathrm{T}}^{{\text {hard}}}$. To account for differences in response between data and simulation, the longitudinal component of $\mathbf E _{\mathrm{T}}^{{\text {miss}},\text {soft}}$ is scaled up and down to give an uncertainty band.

In order to generate the required number of simulated events, some analyses in ATLAS may have to use the fast detector simulation ATLFAST2 [38, 45] for the calorimeter response. It employs parameterisations for electromagnetic and hadronic showers, instead of the explicit simulation of the particle tracking through matter and the energy-loss mechanisms in a detailed detector geometry. An additional uncertainty is assigned to effects introduced by ATLFAST2. This uncertainty contribution only needs to be considered in analyses using this fast simulation, and does not apply for the results presented in this paper. In analyses where it is applicable, it is added in quadrature to the standard uncertainties.

### Systematic uncertainties in $E_{\mathrm{T}}^{\mathrm{miss}}$ response and resolution

The result for the systematic uncertainty of the $E_{\mathrm{T}}^{\mathrm{miss}}$ scale, determined as discussed in the previous section, is summarised in Fig. 13. The average longitudinal projection of $\mathbf p _{\mathrm{T}}^{{\text {soft}}}$ onto $\mathbf p _{\mathrm{T}}^{{\text {hard}}}$, $\langle \mathcal {P} _{\parallel } \rangle$, as a function of $p_{\mathrm{T}}^{{\text {hard}}}$ is shown in Fig. 13a which compares data to both the standard Powheg +Pythia8-based simulations and the alternative MC simulation employing MadGraph, as described in Sect. 4.2. All MC simulation results are expected to have $\langle \mathcal {P} _{\parallel } \rangle _{\mathrm{MC}}$ within the uncertainties of the data. The lower panel of Fig. 13a confirms that the ratio $\langle \mathcal {P} _{\parallel } \rangle _{\mathrm{MC}}/\langle \mathcal {P} _{\parallel } \rangle _{\mathrm{data}}$ lies within the systematic uncertainty band over the full $p_{\mathrm{T}}^{{\text {hard}}}$ range.

The systematic uncertainty for the $E_{\mathrm{T}}^{\mathrm{miss}}$ resolution is extracted from the variances of the parallel ($\mathrm {RMS}^{2}_{\!\parallel }$) and perpendicular ($\mathrm {RMS}^{2}_{\!\perp }$) projections of $\mathbf E _{\mathrm{T}}^{{\text {miss}}}$ onto $\mathbf p _{\mathrm{T}}^{{\text {hard}}}$ defined in Sect. 7.1.2. Figure 13b shows the $p_{\mathrm{T}}^{{\text {hard}}}$ dependence of $\mathrm {RMS}^{2}_{\!\parallel }$ measured for the exclusive $Z \rightarrow \mu \mu$ sample ($N_{\mathrm{jet}} = 0$) in data and two MC simulations. The variances $(\mathrm {RMS}^{2}_{\!\parallel })_{\mathrm{MC}}$ calculated for both sets of simulations agree within the systematic uncertainties of $(\mathrm {RMS}^{2}_{\!\parallel })_{\mathrm{data}}$ with the data, as illustrated in the lower panel of the figure, where the ratio $(\mathrm {RMS}^{2}_{\!\parallel })_{\mathrm{MC}}/(\mathrm {RMS}^{2}_{\!\parallel })_{\mathrm{data}}$ is shown as a function of $p_{\mathrm{T}}^{{\text {hard}}}$. The results of the evaluation of the variances $\mathrm {RMS}^{2}_{\!\perp }$ of the perpendicular projections as a function of $p_{\mathrm{T}}^{{\text {hard}}}$ are shown in Fig. 13c, together with the resulting $p_{\mathrm{T}}^{{\text {hard}}}$ dependence of the ratio $(\mathrm {RMS}^{2}_{\!\perp })_{\mathrm{MC}}/(\mathrm {RMS}^{2}_{\!\perp })_{\mathrm{data}}$. The systematic uncertainties of the data cover all differences to MC simulations.

## Missing transverse momentum reconstruction variants

### Calorimeter-based $E_{\mathrm{T}}^{\mathrm{miss}}$

The $E_{\mathrm{T}}^{\mathrm{miss}}$ soft term from the calorimeter $E_{\mathrm{T}}^{{\text {miss}},{\text {soft}},\text {calo}}$ is reconstructed from topo-clusters. As discussed in Ref. [6], each topo-cluster provides a basic EM scale signal as well as a calibrated signal reconstructed using local cell weighting (LCW), and $E_{\mathrm{T}}^{{\text {miss}},{\text {soft}},\text {calo}}$ is calculated from topo-clusters calibrated at the LCW scale. Only topo-clusters with a calibrated energy $E_{{\text {clus}}}^{\text {LCW}} > 0$, not contributing to the reconstruction of the hard objects used to calculate the hard term given in Eq. (6), are considered for $E_{\mathrm{T}}^{{\text {miss}},{\text {soft}},\text {calo}}$. In addition, topo-clusters that are formed at the same location as the hard object signals are not considered for $E_{\mathrm{T}}^{{\text {miss}},{\text {soft}},\text {calo}}$ even if their signals are not directly contributing to the reconstruction of the hard objects. The fully reconstructed $E_{\mathrm{T}}^{\mathrm{miss}}$ using $E_{\mathrm{T}}^{{\text {miss}},{\text {soft}},\text {calo}}$ is $E_{\mathrm{T}}^{{\text {miss}},\text {calo}}$.

Compared to the reference $E_{\mathrm{T}}^{\mathrm{miss}}$ and $\Sigma E_{\mathrm{T}}$, $E_{\mathrm{T}}^{{\text {miss}},\text {calo}}$ and $\Sigma E_{\mathrm{T}}^{\text {calo}}$ have an enhanced dependence on pile-up, mostly introduced by the soft term. To partly compensate for the irreducible contribution of $p_{\mathrm{T}}$-flow reconstructed from topo-clusters generated by pile-up to $E_{\mathrm{T}}^{{\text {miss}},\text {calo}}$, a modified jet selection and ambiguity resolution is applied in their reconstruction. The considered jets are reconstructed following the prescription in Sect. 3.3.5, and required to have a fully calibrated $p_{\mathrm{T}} > 20\,{\text {Ge}\text {V}}$. The contribution of these jets to $E_{\mathrm{T}}^{{\text {miss}},\text {calo}}$ and $\Sigma E_{\mathrm{T}}^{\text {calo}}$, defined in terms of momentum components $(p_{x},p_{y})$, depends on the overlap with already accepted reconstructed particles,

14 $$\begin{aligned} (p_{x},p_{y}) = \left\{ \begin{array}{lll} (0,0) &{} \kappa _{E} \ge 50\% &{} \text {(large overlap)} \\ (1-\kappa _{E})\times (p_{x}^{\text {jet}},p_{y}^{\text {jet}}) &{} \kappa _{E} < 50\% &{} \text {(small or no overlap)} \\ \end{array} \right. . \end{aligned}$$

The overlap fraction $\kappa _{E}$ is given in Eq. (8). Jets with $\kappa _{E} \ge 50\%$ are not used at all. The JVT-based tagging of non-pile-up jets is omitted. It is found that this strategy reduces the fluctuations in the $E_{\mathrm{T}}^{{\text {miss}},\text {calo}}$ reconstruction. The transverse momentum contribution of groups of clusters representing a jet-like $p_{\mathrm{T}}$-flow e.g. from pile-up in a given direction that are not reconstructed and calibrated as a jet, or do not pass the jet-$p_{\mathrm{T}}$ threshold applied in $E_{\mathrm{T}}^{\mathrm{miss}}$ reconstruction, is reduced if all jets and jet fragments, including those from pile-up, are included.

### $E_{\mathrm{T}}^{\mathrm{miss}}$ from tracks

The reference track-based soft term $E_{\mathrm{T}}^{{\text {miss}},{\text {soft}}}$ is largely insensitive to pile-up, as indicated by the dependence of the $E_{\mathrm{T}}^{\mathrm{miss}}$ resolution $\mathrm {RMS}^{\mathrm{miss}}_{x(y)}$ on $N_{\mathrm{PV}}$ in the exclusive $Z \rightarrow \mu \mu$ sample ($N_{\mathrm{jet}} = 0$) shown in Fig. 7c. As discussed in Sect. 6.2.4 and from the comparison of Fig. 7c, d, the pile-up dependence of $\mathrm {RMS}^{\mathrm{miss}}_{x(y)}$ in the inclusive $Z \rightarrow \mu \mu$ sample is largely introduced by the jet contribution. This contribution suffers from (1) the lack of pile-up suppression for forward jets with $|\eta | > 2.4$, (2) any inefficiency connected with the JVT-based tagging, and (3) irreducible pile-up-induced fluctuations in the calorimeter jet signals. Using a representation of $E_{\mathrm{T}}^{\mathrm{miss}}$ employing only reconstructed ID tracks from the primary vertex increases stability against pile-up as long as the tracking and vertex resolution is not affected by it. In this representation ($p_{\mathrm{T}}^{\mathrm{miss}}$) all jets and reconstructed particles are ignored, i.e. the $p_{\mathrm{T}}^{\mathrm{miss}}$ reconstruction does not include any calorimeter or MS signals. The $p_{\mathrm{T}}^{\mathrm{miss}}$ resolution is then inherently immune to pile-up, while the $p_{\mathrm{T}}^{\mathrm{miss}}$ response is low as all neutral $p_{\mathrm{T}}$-flow in $|\eta | < 2.5$ as well as all $p_{\mathrm{T}}$-flow outside of this region is excluded.

### Performance evaluations for $E_{\mathrm{T}}^{\mathrm{miss}}$ variants

The main motivation to study $E_{\mathrm{T}}^{\mathrm{miss}}$-reconstruction variants is to improve some combination of the $E_{\mathrm{T}}^{\mathrm{miss}}$ resolution, scale, and stability against pile-up. As with the composition of objects entering $E_{\mathrm{T}}^{\mathrm{miss}}$ reconstruction in general, the particular choice of variant used for a given analysis strongly depends on the performance requirements for this analysis. The comparison of both the resolution and response of $E_{\mathrm{T}}^{{\text {miss}},\text {calo}}$ and $p_{\mathrm{T}}^{\mathrm{miss}}$ to the corresponding measurements using the reference $E_{\mathrm{T}}^{\mathrm{miss}}$ illustrates their principal features for the $Z \rightarrow \mu \mu$ and $t\bar{t}$ production final state.

**Fig. 14:**
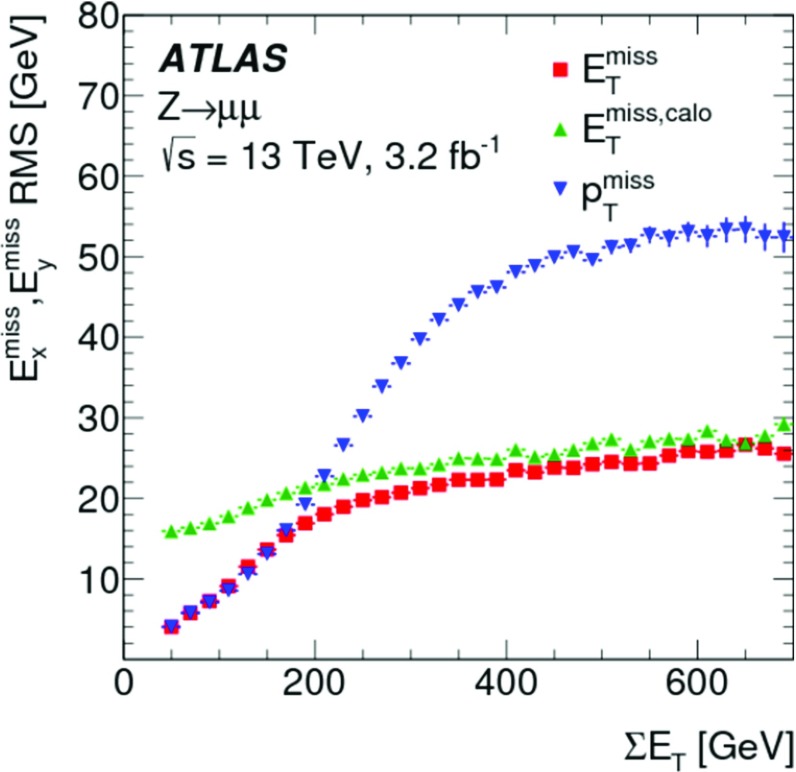
Comparison of the reference $E_{\mathrm{T}}^{\mathrm{miss}}$ resolution with the resolutions of the track-only-based variant $p_{\mathrm{T}}^{\mathrm{miss}}$ described in Sect. 8.2, and the reconstruction variant $E_{\mathrm{T}}^{{\text {miss}},\text {calo}}$ employing a calorimeter-based soft term, as discussed in Sect. 8.1. The resolutions are determined as described in Sect. 6.2.3 and shown as a function of the $\Sigma E_{\mathrm{T}}$. For consistency, for all three variants, the $\Sigma E_{\mathrm{T}}$ value is taken from $E_{\mathrm{T}}^{\mathrm{miss}}$

**Fig. 15:**
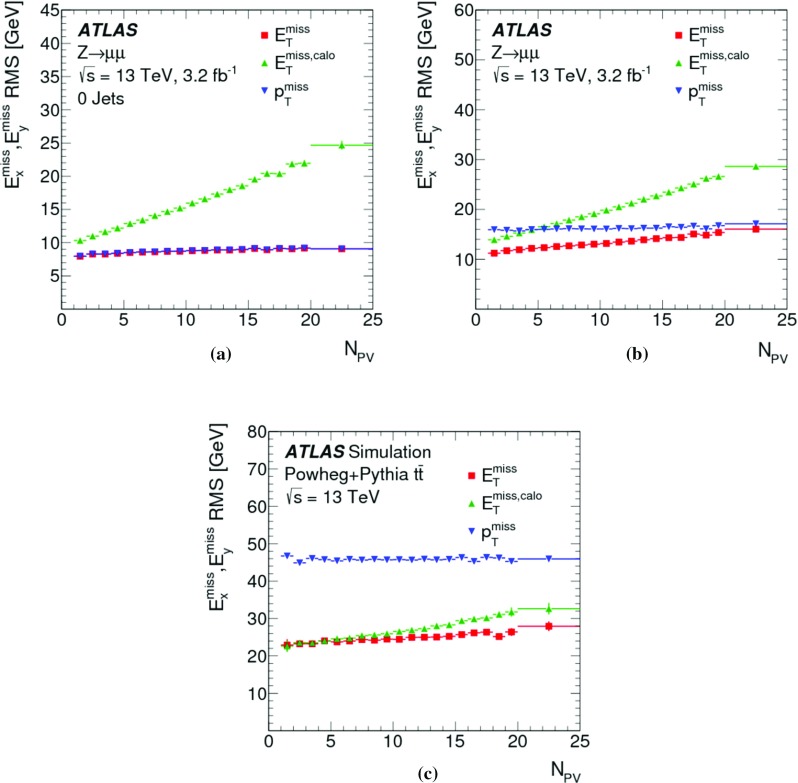
Comparison of the reference $E_{\mathrm{T}}^{\mathrm{miss}}$ resolution with the resolutions of the track-only-based variant $p_{\mathrm{T}}^{\mathrm{miss}}$ described in Sect. 8.2, and the reconstruction variant $E_{\mathrm{T}}^{{\text {miss}},\text {calo}}$ employing a calorimeter-based soft term, as discussed in Sect. 8.1. The resolutions are determined as described in Sect. 6.2.3 and shown as a function of the pile-up activity measured in terms of the number of reconstructed vertices $N_{\mathrm{PV}}$ for **a** an exclusive $Z \rightarrow \mu \mu$ sample without jets with $p_{\mathrm{T}} > 20\,{\text {Ge}\text {V}}$ and **b** an inclusive $Z \rightarrow \mu \mu$ sample, both selected from data. In **c**, the resolution of the $E_{\mathrm{T}}^{\mathrm{miss}}$ reconstruction-variants in a final state with significant jet activity and $p_{\mathrm{T}}^{\nu } > 0$ is compared using MC simulations of $t\bar{t}$ production

#### Comparisons of $E_{\mathrm{T}}^{\mathrm{miss}}$ resolution

Figure 14 compares the $E_{\mathrm{T}}^{{\text {miss}},\text {calo}}$ and $p_{\mathrm{T}}^{\mathrm{miss}}$ resolutions with the one obtained from the reference $E_{\mathrm{T}}^{\mathrm{miss}}$, for the inclusive $Z \rightarrow \mu \mu$ sample in data. Each is shown as a function of $\Sigma E_{\mathrm{T}}$ corresponding to the reference $E_{\mathrm{T}}^{\mathrm{miss}}$, giving an estimate of the total hard-scatter activity. The low-$\Sigma E_{\mathrm{T}}$ region is dominated by events with $N_{\mathrm{jet}} = 0$, where the contribution of $E_{\mathrm{T}}^{{\text {miss}},{\text {soft}},\text {calo}}$ in $E_{\mathrm{T}}^{{\text {miss}},\text {calo}}$ yields a poorer resolution than for $E_{\mathrm{T}}^{\mathrm{miss}}$, and where $E_{\mathrm{T}}^{\mathrm{miss}}$ and $p_{\mathrm{T}}^{\mathrm{miss}}$ have identical performance. The high-$\Sigma E_{\mathrm{T}}$ region is dominated by events with higher jet multiplicity, where $p_{\mathrm{T}}^{\mathrm{miss}}$ resolution is degraded relative to the reference $E_{\mathrm{T}}^{\mathrm{miss}}$ by the incomplete measurement of jets.

Figure 15a compares the $E_{\mathrm{T}}^{{\text {miss}},\text {calo}}$ and $p_{\mathrm{T}}^{\mathrm{miss}}$ resolution as functions of the pile-up activity measured by $N_{\mathrm{PV}}$, with the one obtained from the reference $E_{\mathrm{T}}^{\mathrm{miss}}$ for the exclusive $Z \rightarrow \mu \mu$ samples with $N_{\mathrm{jet}} = 0$ in data. The $E_{\mathrm{T}}^{{\text {miss}},\text {calo}}$ resolution is dominated by pile-up and shows significantly degraded performance relative to $p_{\mathrm{T}}^{\mathrm{miss}}$ and the reference $E_{\mathrm{T}}^{\mathrm{miss}}$. The exclusive use of only tracks from the hard-scatter vertex for both $p_{\mathrm{T}}^{\mathrm{miss}}$ and $E_{\mathrm{T}}^{\mathrm{miss}}$ yields the same stability against pile-up.

In events with jet activity, the degraded $p_{\mathrm{T}}^{\mathrm{miss}}$ resolution is observable, especially outside the region of highest pile-up activity, as seen in Fig. 15b for the $E_{\mathrm{T}}^{\mathrm{miss}}$ resolution obtained with the inclusive $Z \rightarrow \mu \mu$ sample in data for $N_{\mathrm{PV}} \lesssim 15$. This is even more obvious in final states with relatively high jet multiplicity and genuine missing transverse momentum, like for the $t\bar{t}$-production sample from MC simulations. As shown in Fig. 15c for this final state, both the reference $E_{\mathrm{T}}^{\mathrm{miss}}$ and the calorimeter-based $E_{\mathrm{T}}^{{\text {miss}},\text {calo}}$ have a significantly better resolution than $p_{\mathrm{T}}^{\mathrm{miss}}$, at the price of some sensitivity to pile-up, which is absent for $p_{\mathrm{T}}^{\mathrm{miss}}$. The $N_{\mathrm{PV}}$ dependence of the resolution is enhanced in $E_{\mathrm{T}}^{{\text {miss}},\text {calo}}$, due to the increased contribution from soft calorimeter signals without pile-up suppression at higher $N_{\mathrm{PV}}$.

#### Comparisons of $E_{\mathrm{T}}^{\mathrm{miss}}$ scale

**Fig. 16:**
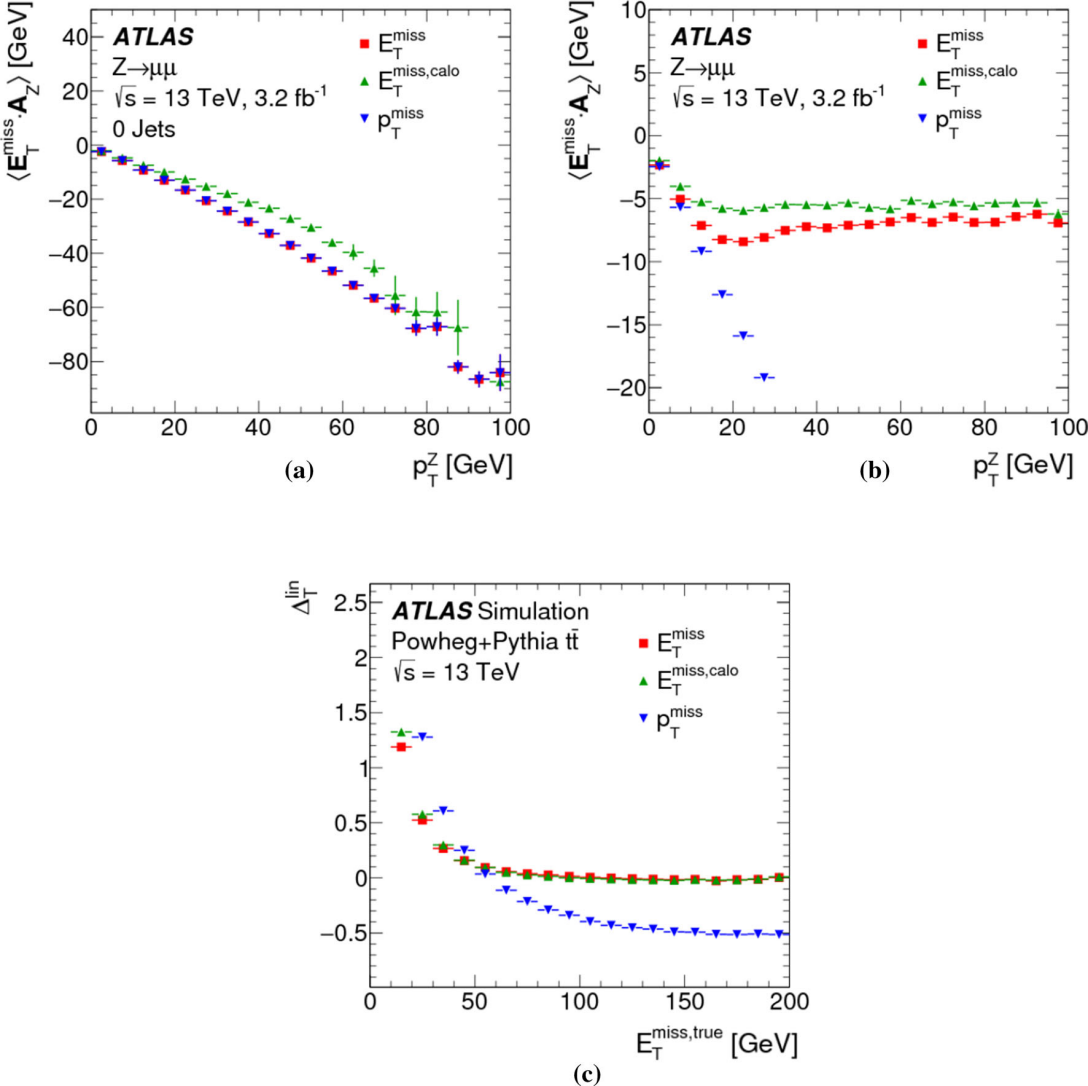
Comparison of the reference $E_{\mathrm{T}}^{\mathrm{miss}}$, the calorimeter-based $E_{\mathrm{T}}^{{\text {miss}},\text {calo}}$ and track-only-based $p_{\mathrm{T}}^{\mathrm{miss}}$ response in an **a** exclusive and an **b** inclusive $Z \rightarrow \mu \mu$ sample from data. The projections of the respective $\mathbf E _{\mathrm{T}}^{{\text {miss}}}$, $\mathbf E _{\mathrm{T}}^{{\text {miss}},\text {calo}}$, and $\mathbf p _{\mathrm{T}}^{\mathrm{miss}}$ onto the direction of $\mathbf p _{\mathrm{T}}^{Z}$, calculated according to Eqs. (9) and (10), are shown as a function of $p_{\mathrm{T}}^{Z}$. In **c**, the linearity of the reference $E_{\mathrm{T}}^{\mathrm{miss}}$, $E_{\mathrm{T}}^{{\text {miss}},\text {calo}}$, and $p_{\mathrm{T}}^{\mathrm{miss}}$ scales, calculated according to Eq. (11), is shown as a function of the true $E_{\mathrm{T}}^{{\text {miss}},\text {true}}$ for the $t\bar{t}$-production MC simulation sample

Following the description in Sect. 6.2.1, the $E_{\mathrm{T}}^{\mathrm{miss}}$ response is evaluated for the reference $E_{\mathrm{T}}^{\mathrm{miss}}$, $E_{\mathrm{T}}^{{\text {miss}},\text {calo}}$, and $p_{\mathrm{T}}^{\mathrm{miss}}$ using the respective projections of $\mathbf E _{\mathrm{T}}^{{\text {miss}}}$, $\mathbf E _{\mathrm{T}}^{{\text {miss}},\text {calo}}$, and $\mathbf p _{\mathrm{T}}^{\mathrm{miss}}$ onto the direction of $\mathbf p _{\mathrm{T}}^{Z}$, according to Eqs. (9) and (10). Figure 16a shows the average projection as a function of $p_{\mathrm{T}}^{Z}$ for the exclusive $Z \rightarrow \mu \mu$ sample with $N_{\mathrm{jet}} = 0$ in data. Both $E_{\mathrm{T}}^{\mathrm{miss}}$ and $p_{\mathrm{T}}^{\mathrm{miss}}$ show the same increasingly incomplete reconstruction of the hadronic recoil in this sample for rising $p_{\mathrm{T}}^{Z}$. This reconstruction is slightly improved for $E_{\mathrm{T}}^{{\text {miss}},\text {calo}}$, but still insufficient at higher $p_{\mathrm{T}}^{Z}$.

In the inclusive $Z \rightarrow \mu \mu$ sample, shown in Fig. 16b, the indication at lower $p_{\mathrm{T}}^{Z}$ is that $E_{\mathrm{T}}^{{\text {miss}},\text {calo}}$ has a higher response and thus a better representation of the hadronic recoil, due to the more complete $E_{\mathrm{T}}^{{\text {miss}},{\text {soft}}}$ reconstruction and the lack of a JVT-tagging requirement. This effect is partly due to the observation bias in the response introduced by the relatively poor $E_{\mathrm{T}}^{{\text {miss}},\text {calo}}$ resolution, as discussed in Sect. 3.1. Both $E_{\mathrm{T}}^{\mathrm{miss}}$ and $E_{\mathrm{T}}^{{\text {miss}},\text {calo}}$ show comparable response for $p_{\mathrm{T}}^{Z} \gtrsim 60\,{\text {Ge}\text {V}}$, owing to the JVT cut-off at $60\,{\text {Ge}\text {V}}$. The slightly larger $E_{\mathrm{T}}^{{\text {miss}},\text {calo}}$ response of about $1\,{\text {Ge}\text {V}}$ reflects the contribution from neutral signals to the soft term. The degraded response associated with $p_{\mathrm{T}}^{\mathrm{miss}}$ related to the exclusion of hard objects is clearly visible in this figure.

Figure 16c shows the linearity of the various $E_{\mathrm{T}}^{\mathrm{miss}}$ reconstruction approaches as a function of $E_{\mathrm{T}}^{{\text {miss}},\text {true}}$ for the $t\bar{t}$-production sample from MC simulations. Beyond $E_{\mathrm{T}}^{{\text {miss}},\text {true}} \approx 60\,{\text {Ge}\text {V}}$ both the reference $E_{\mathrm{T}}^{\mathrm{miss}}$ and $E_{\mathrm{T}}^{{\text {miss}},\text {calo}}$ show the same good linearity, while the lack of a jet contribution to $p_{\mathrm{T}}^{\mathrm{miss}}$ shows a loss of response up to about 50% at higher $E_{\mathrm{T}}^{{\text {miss}},\text {true}}$. The overestimation of $E_{\mathrm{T}}^{{\text {miss}},\text {true}}$ by all three reconstruction variants at lower $E_{\mathrm{T}}^{{\text {miss}},\text {true}}$ reflects the observation bias in the response introduced by the resolution. The poorer resolution associated with $p_{\mathrm{T}}^{\mathrm{miss}}$ observed in Fig. 15c for this sample leads to a faster rise of the response with decreasing $E_{\mathrm{T}}^{{\text {miss}},\text {true}}$ than for the reference $E_{\mathrm{T}}^{\mathrm{miss}}$ and $E_{\mathrm{T}}^{{\text {miss}},\text {calo}}$, which show a very similar dependence on $E_{\mathrm{T}}^{{\text {miss}},\text {true}}$.

#### Summary of performance

Both $E_{\mathrm{T}}^{{\text {miss}},\text {calo}}$ and $p_{\mathrm{T}}^{\mathrm{miss}}$ offer alternative measures for $E_{\mathrm{T}}^{\mathrm{miss}}$. The calorimeter-based $E_{\mathrm{T}}^{{\text {miss}},\text {calo}}$ uses topo-clusters calibrated at the LCW scale for the soft term, which are neither part of the signal nor otherwise overlapping with the signals of other hard objects contributing to $E_{\mathrm{T}}^{\mathrm{miss}}$. This introduces a pile-up dependence into $E_{\mathrm{T}}^{{\text {miss}},\text {calo}}$, due to the lack of pile-up suppression of calorimeter signals outside of reconstructed hard objects. It features a slightly modified jet contribution without the JVT-based selection used in case of the reference $E_{\mathrm{T}}^{\mathrm{miss}}$ reconstruction, to allow the cancellation of jet-like $p_{\mathrm{T}}$-flow from pile-up in $E_{\mathrm{T}}^{{\text {miss}},{\text {soft}},\text {calo}}$ by pile-up jets in its hard term. The $E_{\mathrm{T}}^{{\text {miss}},\text {calo}}$ response in the inclusive $Z \rightarrow \mu \mu$ sample is better than the reference $E_{\mathrm{T}}^{\mathrm{miss}}$ response, in particular in the region of small hadronic recoil ($p_{\mathrm{T}}^{Z} \lesssim 20\,{\text {Ge}\text {V}}$). It is comparable to the reference in $t\bar{t}$ final states. The observed $\mathrm {RMS}^{\mathrm{miss}}_{x(y)}$, in particular in $Z \rightarrow \mu \mu$ without jets, is significantly more affected by pile-up than is the reference $E_{\mathrm{T}}^{\mathrm{miss}}$ or the track-only-based $p_{\mathrm{T}}^{\mathrm{miss}}$. In final states with a considerable number of jets, like $t\bar{t}$, $E_{\mathrm{T}}^{{\text {miss}},\text {calo}}$ performs nearly as well as the reference $E_{\mathrm{T}}^{\mathrm{miss}}$, with a slight degradation of the $E_{\mathrm{T}}^{\mathrm{miss}}$ resolution at highest pile-up activities. This variant is useful for physics analyses least sensitive to the soft-term contribution to $E_{\mathrm{T}}^{\mathrm{miss}}$ resolution but requiring a linear $E_{\mathrm{T}}^{\mathrm{miss}}$ response.

The track-only-based $p_{\mathrm{T}}^{\mathrm{miss}}$ displays a degraded response for the inclusive $Z \rightarrow \mu \mu$ sample, which is expected from the exclusive use of hard-scatter-vertex tracks. As expected, resolution is not affected by pile-up in the considered final states, but is poorer than, or at most comparable to, the reference $E_{\mathrm{T}}^{\mathrm{miss}}$ algorithm. Nevertheless, $p_{\mathrm{T}}^{\mathrm{miss}}$ provides a stable observable for event and phase-space selections in analyses sensitive to $E_{\mathrm{T}}^{\mathrm{miss}}$ resolution.

## Conclusion

The performance and features of the missing transverse momentum reconstruction in $pp$ collision data at the LHC, acquired in 2015 with the ATLAS detector at $\sqrt{s} = 13\,{\text {Te}\text {V}}$ and corresponding to about $3.2~\hbox {fb}^{-1}$, are evaluated for selected event samples with ($W \rightarrow e\nu$, $W \rightarrow \mu \nu$, $t\bar{t}$) and without ($Z \rightarrow \mu \mu$) genuine $E_{\mathrm{T}}^{\mathrm{miss}}$. The comparison of the data from the detector with the corresponding MC simulations generally yields good agreement in the covered phase space. The systematic uncertainty contribution from the soft event to the reconstructed $E_{\mathrm{T}}^{\mathrm{miss}}$ is determined with $Z \rightarrow \mu \mu$ final states without jets. It is calculated from the data-to-MC-simulations comparison of the parallel and perpendicular projections of the missing transverse momentum vector $\mathbf E _{\mathrm{T}}^{{\text {miss}}}$ onto the vector sum of the transverse momenta of the hard objects $\mathbf p _{\mathrm{T}}^{{\text {hard}}}$. The parallel projections yield the uncertainty of the $E_{\mathrm{T}}^{\mathrm{miss}}$ scale, evaluated as a function of the total transverse momentum of the hard objects ($p_{\mathrm {T}}^{\mathrm {hard}}$). The widths of the distributions of the parallel and perpendicular projections yield the respective systematic uncertainties of the $E_{\mathrm{T}}^{\mathrm{miss}}$ resolution. Simulation tends to underestimate the perpendicular resolution and overestimate the scale and parallel resolution, in each case differing from data by at most 10%.

The performance evaluation of $E_{\mathrm{T}}^{\mathrm{miss}}$ response and resolution for the inclusive $Z \rightarrow \mu \mu$ sample shows that data and MC simulations agree within the systematic uncertainties. The $E_{\mathrm{T}}^{\mathrm{miss}}$ response shows an underestimation of the soft contributions to $E_{\mathrm{T}}^{\mathrm{miss}}$. A degradation of the $E_{\mathrm{T}}^{\mathrm{miss}}$ resolution is observed for increasing $\Sigma E_{\mathrm{T}}$ and $N_{\mathrm{PV}}$, due to pile-up and detector resolution effects. Additional performance measures considered in these studies include the estimate of tails in the $E_{\mathrm{T}}^{\mathrm{miss}}$ distribution. As expected from the universality of the hadronic recoil, the integral tail fraction of the $E_{\mathrm{T}}^{\mathrm{miss}}$ distribution is identical for inclusive $Z$ and $W$ boson production, independent of the leptonic decay mode. The $t\bar{t}$ final states feature a higher jet multiplicity and show larger tails reflecting a higher sensitivity to residual pile-up surviving in the jet contribution to $E_{\mathrm{T}}^{\mathrm{miss}}$, in terms of the inclusion of pile-up jets as well as the increased fluctuations of the jet response introduced by pile-up.

From the performance studies presented in this paper, the object-based $E_{\mathrm{T}}^{\mathrm{miss}}$ reconstruction in ATLAS, which was developed for LHC $\text {Run}\,1$ and used in a large number of physics analyses, can be used with the discussed refinements and adjustments for $\text {Run}\,2$ as well.

## Figures and Tables

**Fig. 17:**
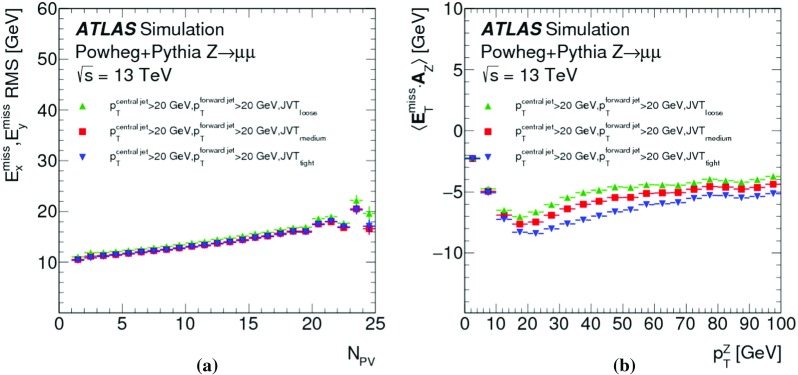
$E_{\mathrm{T}}^{\mathrm{miss}}$ resolution and response for the different choices of JVT discussed in the text, as measured for $Z \rightarrow \mu \mu$ events in MC simulation. The **a** $E_{\mathrm{T}}^{\mathrm{miss}}$ resolution is shown as function of the pile-up activity measured by the number of primary vertices $N_{\mathrm{PV}}$, and the **b** $E_{\mathrm{T}}^{\mathrm{miss}}$ response is shown as function of the transverse momentum $p_{\mathrm{T}}^{Z}$ of the $Z$ boson. A global selection of $p_{\mathrm{T}} > 20\,{\text {Ge}\text {V}}$ is applied to the transverse momentum of jets within $|\eta | < 2.4$ ($p_{\mathrm{T}}^{\text {central jet}}$) and for forward jets with $|\eta | \ge 2.4$ ($p_{\mathrm{T}}^{\text {forward jet}}$)

**Fig. 18:**
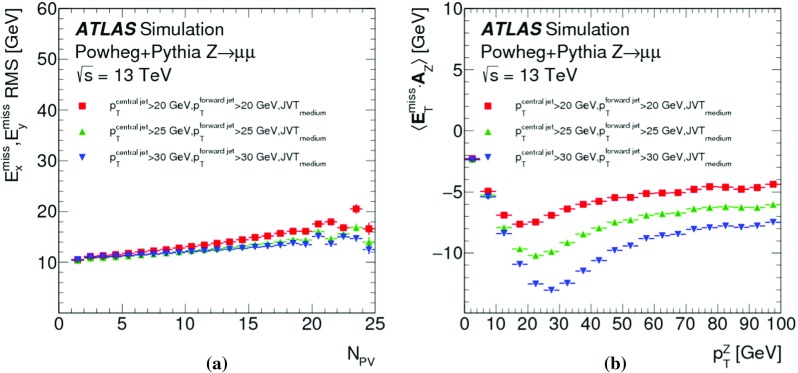
$E_{\mathrm{T}}^{\mathrm{miss}}$ resolution and scale for different global jet-$p_{\mathrm{T}}$ thresholds, for $Z \rightarrow \mu \mu$ events in MC simulation. The **a** $E_{\mathrm{T}}^{\mathrm{miss}}$ resolution is shown as function of the pile-up activity measured by the number of primary vertices $N_{\mathrm{PV}}$, and the **b** $E_{\mathrm{T}}^{\mathrm{miss}}$ response is shown as function of the transverse momentum $p_{\mathrm{T}}^{Z}$ of the $Z$ boson. The same respective thresholds are applied to the transverse momentum of jets within $|\eta | < 2.4$ ($p_{\mathrm{T}}^{\text {central jet}}$) and for forward jets with $|\eta | \ge 2.4$ ($p_{\mathrm{T}}^{\text {forward jet}}$), with $p_{\mathrm{T}}^{\text {central jet}}, p_{\mathrm{T}}^{\text {forward jet}} > \{ 20, 25, 30 \}\,{\text {Ge}\text {V}}$

**Fig. 19:**
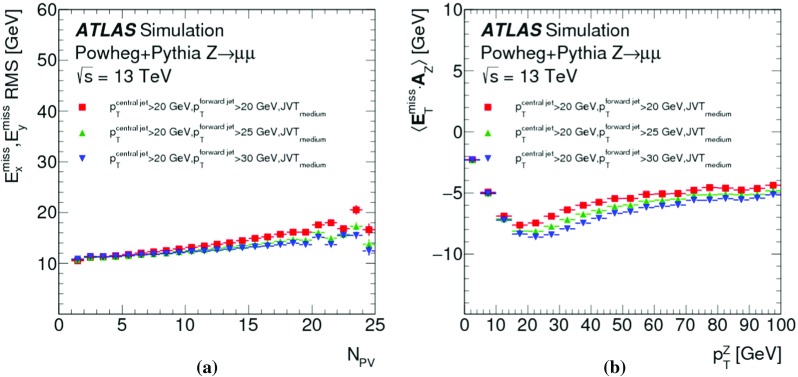
$E_{\mathrm{T}}^{\mathrm{miss}}$ resolution and scale for different regional jet-$p_{\mathrm{T}}$ thresholds, for $Z \rightarrow \mu \mu$ events in MC simulation. The **a** $E_{\mathrm{T}}^{\mathrm{miss}}$ resolution is shown as function of the pile-up activity measured by the number of primary vertices $N_{\mathrm{PV}}$, and the **b** $E_{\mathrm{T}}^{\mathrm{miss}}$ response is shown as function of the transverse momentum $p_{\mathrm{T}}^{Z}$ of the $Z$ boson. The same threshold is applied to the transverse momentum of jets within $|\eta | < 2.4$ ($p_{\mathrm{T}}^{\text {central jet}} > 20\,{\text {Ge}\text {V}}$), while for forward jets with $|\eta | \ge 2.4$ threshold variations ($p_{\mathrm{T}}^{\text {forward jet}} > \{20, 25,30\}\,{\text {Ge}\text {V}}$) are studied

## Appendix A: Glossary of terms

In this paper several acronyms and qualifiers are used to describe the reconstruction of $E_{\mathrm{T}}^{\mathrm{miss}}$ and related observables. This brief glossary of terms is intended to help with the nomenclature. All terms should be interpreted in the context of $E_{\mathrm{T}}^{\mathrm{miss}}$ reconstruction and may have other interpretations in other contexts.

- ${{\varvec{E}}}_\mathbf{T }^\mathbf{miss }$ **reconstruction**: Using this nomenclature usually encompasses the reconstruction of a set of observables comprising the missing transverse momentum vector $\mathbf E _{\mathrm{T}}^{{\text {miss}}}$, its components $E_{x(y)}^{{\text {miss}}}$, and its absolute value $E_{\mathrm{T}}^{\mathrm{miss}}$. In addition, the scalar sum $\Sigma E_{\mathrm{T}}$ of the $p_{\mathrm{T}}$ of all kinematic objects contributing to $\mathbf E _{\mathrm{T}}^{{\text {miss}}}$ is calculated. The calculation of these variables is described in detail in Eqs. (1)–(4) in Sect. 3.1.
- **Hard scatter** and **primary vertices**: The hardest $pp$ interaction in a given event is referred to as the *hard scatter*. It is normally associated with a reconstructed *hard-scatter vertex*, which is considered the hardest vertex among all reconstructed *primary vertices* in this event. The hard-scatter vertex is defined as the one with the largest sum of $p_{\mathrm{T}}^{2}$ of tracks associated with it. The other primary vertices are assumed to be produced by in-time pile-up interactions. The variable $N_{\mathrm{PV}}$ denotes the number of reconstructed primary collision vertices in the event.
- **Hard event** and **hard term**: The reconstruction of *hard objects* includes individual particles such as electrons, photons, muons and $\tau \text {-leptons}$, and jets. In all cases the final objects are characterised by a kinematic threshold and reconstruction quality requirements. Both the reconstructed charged-particle tracks from the ID and topo-clusters from the calorimeter are used as the input signals for these objects. In the context of $E_{\mathrm{T}}^{\mathrm{miss}}$ reconstruction, the use of the same detector signals by different hard objects is excluded, see details in Sect. 3. The finally accepted hard event objects give rise to the *hard term* in $E_{\mathrm{T}}^{\mathrm{miss}}$ reconstruction.
- **Soft event** and **soft term**: All detector signals recorded for one triggered event and not used by the hard objects discussed above can be considered as *soft signals* contributing to $E_{\mathrm{T}}^{\mathrm{miss}}$. They include signals or signal traces from scattered soft particles arising from the underlying event accompanying the hard-scatter interaction, or from statistically completely independent pile-up interactions producing diffuse particle emissions in the same bunch crossing. In addition, signals from particles and jets which do not satisfy the hard-object quality criteria, or are below the kinematic threshold, can be included in the soft event. The reference $E_{\mathrm{T}}^{\mathrm{miss}}$ reconstruction configuration for the results presented in Sects. 6 and 7 uses reconstructed ID tracks from the soft event to form the *soft term* in $E_{\mathrm{T}}^{\mathrm{miss}}$ reconstruction, with the track selection details outlined under priority (6) in Table 1 in Sect. 3. Alternative configurations employ topo-clusters from the calorimeter, see Table 2 in Appendix A.

## Appendix B: Alternative $E_{\mathrm{T}}^{\mathrm{miss}}$ composition

Table 2 summarises the $E_{\mathrm{T}}^{\mathrm{miss}}$ reconstruction configurations employing only ID tracks, or using topo-clusters for the soft term.

**Table 2:** Representations of $E_{\mathrm{T}}^{\mathrm{miss}}$ and $\Sigma E_{\mathrm{T}}$ calculated from (1) reconstructed charged-particle tracks from the ID or (2) using a soft term from topo-clusters in the calorimeter only

$\#$	Objects contributing to $E_{\mathrm{T}}^{\mathrm{miss}}$ and $\Sigma E_{\mathrm{T}}$			
	Type	Selections	Variables	Comments
(1)	ID track	$|\eta | < 2.5$	$p_{\mathrm{T}}^{\mathrm{miss}}$	Charged-particle-based estimators for $E_{\mathrm{T}}^{\mathrm{miss}}$, $\Sigma E_{\mathrm{T}}$ using all reconstructed tracks from the hard-scatter vertex passing requirements for high-quality reconstruction in addition to kinematic selections
		${p_{\mathrm{T}} > 400\,{\text {Me}\text {V}}}$	$\Sigma p_{\mathrm{T}}$	
		$${|d_{0}| < 1.5\,{\text {mm}}}$$		
		$${|z_{0}\sin (\theta )| < 1.5\,{\text {mm}}}$$		
(2)	Topo-cluster	$E_{{\text {clus}}}^{\text {LCW}}$ > 0 (soft term)	$E_{\mathrm{T}}^{{\text {miss}},{\text {soft}},\text {calo}}$	Variant reconstructing $E_{\mathrm{T}}^{{\text {miss}},\text {calo}}$ ($\Sigma E_{\mathrm{T}}^{\text {calo}}$) using a soft term $E_{\mathrm{T}}^{{\text {miss}},{\text {soft}},\text {calo}}$ ($\Sigma E_{\mathrm{T}}^{{\text {soft}},\text {calo}}$) reconstructed from topo-clusters not used by, or not overlapping with, the hard objects used for the hard term composed of items (1)–(5) in Table 1, with the jet selection described in Sect. 8.1 applied
		$$\Sigma E_{\mathrm{T}}^{{\text {soft}},\text {calo}}$$		

## Appendix C: Jet selection

As discussed in Sect. 3.3.5, jets that are not rejected by the signal ambiguity resolution and have $p_{\mathrm{T}} > 60\,{\text {Ge}\text {V}}$ contribute to $E_{\mathrm{T}}^{\mathrm{miss}}$ reconstruction. Jets with less transverse momentum that fall within $|\eta | < 2.4$ are subjected to further selection based on JVT calculated by a track-based jet vertex tagger [16]. Three values for JVT, each representing a different efficiency for the reconstruction of non-pile-up jets, were considered in the course of the optimisation of the JVT-based selection,

- $\text {JVT} _{\mathrm{tight}} > 0.11 \ldots$ tight selection with high pile-up rejection power at lower signal efficiency;
- $\text {JVT} _{\mathrm{medium}} > 0.59 \ldots$ medium selection with good signal efficiency and pile-up rejection power;
- $\text {JVT} _{\mathrm{loose}} > 0.91 \ldots$ loose selection with lower pile-up rejection power and higher signal efficiency.

The effects of these selections on the $E_{\mathrm{T}}^{\mathrm{miss}}$ resolution $\mathrm {RMS}^{\mathrm{miss}}_{x(y)}$ and response are shown in Fig. 17, for $Z \rightarrow \mu \mu$ events in MC simulation. Figure 17a shows that the pile-up dependence of $\mathrm {RMS}^{\mathrm{miss}}_{x(y)}$ is not significantly affected by the choice for JVT. However, the $E_{\mathrm{T}}^{\mathrm{miss}}$ response measured by the projection given in Eq. (10) in Sect. 6.2.1 and evaluated as a function of $p_{\mathrm{T}}^{Z}$ in the same sample, shows significant sensitivity to the choice of JVT, as seen in Fig. 17b.

In addition to the signal ambiguity resolution and the choice for JVT, the contribution from jets in $E_{\mathrm{T}}^{\mathrm{miss}}$ reconstruction is controlled by a kinematic threshold requiring the transverse momentum of the jet to be $p_{\mathrm{T}} > 20\,{\text {Ge}\text {V}}$. The effects of variations of this threshold on $\mathrm {RMS}^{\mathrm{miss}}_{x(y)}$ and the $E_{\mathrm{T}}^{\mathrm{miss}}$ response are shown in Fig. 18. Increasing the threshold to $30\,{\text {Ge}\text {V}}$ for all jets satisfying the $\text {JVT} _{\mathrm{medium}}$ condition reduces the pile-up dependence of the resolution shown in Fig. 17a, but leads to significant loss of $E_{\mathrm{T}}^{\mathrm{miss}}$ response, as seen in Fig. 17b. Depending on the sensitivities observed in a given physics analysis, the $p_{\mathrm{T}}$-threshold choice for the jet contribution to $E_{\mathrm{T}}^{\mathrm{miss}}$ reconstruction needs to be adjusted to meet the required performance.

Extending the $p_{\mathrm{T}}$ threshold studies with the option of regional thresholds yields the performance results presented in Fig. 19. In this case jets within $|\eta | < 2.4$ are subjected to the $p_{\mathrm{T}}^{\text {central jet}} > 20\,{\text {Ge}\text {V}}$ selection, while jets outside of this $\eta$ range are filtered using $p_{\mathrm{T}}^{\text {forward jet}} > \{20, 25, 30\}\,{\text {Ge}\text {V}}$. This leads to the improvements in the pile-up dependence of $\mathrm {RMS}^{\mathrm{miss}}_{x(y)}$ shown in Fig. 19a, which are very similar to the ones observed in Fig. 18a for a global jet-$p_{\mathrm{T}}$ threshold variation. The comparison indicates that the main pile-up contribution to $\mathrm {RMS}^{\mathrm{miss}}_{x(y)}$ is introduced by forward jets, for which no JVT-based pile-up-mitigation is available.

Increasing the $p_{\mathrm{T}}$ threshold only for forward jets reduces the average loss of response observed in case of the global $p_{\mathrm{T}}$ threshold increase. This can be seen by comparing the results shown in Fig. 19b for regional $p_{\mathrm{T}}$ thresholds with the ones shown in Fig. 18b for global thresholds. Like for the JVT threshold selection, the choice of the appropriate global or regional $p_{\mathrm{T}}$-threshold depends on the $E_{\mathrm{T}}^{\mathrm{miss}}$ reconstruction performance required in the context of a given analysis.

## Footnotes

1 ATLAS uses a right-handed coordinate system with its origin at the nominal interaction point (IP) in the centre of the detector and the *z*-axis along the beam pipe. The *x*-axis points from the IP to the centre of the LHC ring, and the *y*-axis points upwards. Cylindrical coordinates $(r,\phi )$ are used in the transverse plane, $\phi$ being the azimuthal angle around the *z*-axis. The pseudorapidity is defined in terms of the polar angle $\theta$ as $\eta = -\ln \tan (\theta /2)$. Angular distance is measured in units of $\Delta R \equiv \sqrt{(\Delta \eta )^{2} + (\Delta \phi )^{2}}$.

2 In this formula the notion of *selected*, which is only applicable to electrons and muons, means that the choice of reconstructed particles is purely given by a set of criteria similar to those given in Sects. 3.3.1 and 3.3.4, respectively, with possible modifications imposed by a given analysis. The notion of *accepted* indicates a modification of the set of initially selected objects imposed by the signal ambiguity resolution.

3 On this scale the energy deposited in the calorimeter by electrons and photons is represented well. The hadron signal at the EM scale is not corrected for the non-compensating signal features of the ATLAS calorimeters.

4 In the calculation of JVT the total amount of $p_{\mathrm{T}}$ carried by tracks from the hard-scatter vertex matched to the given jet is related to the total amount of $p_{\mathrm{T}}$ carried by all matched tracks, among other inputs, to tag jets from the hard-scatter interaction.

5 Version 8.186 was used for all final states generated with Pythia8.

6 In ATLAS stable particles are those with an expected laboratory lifetime $\tau$ corresponding to $c\tau > 10\,{\text {mm}}$.

7 This lower boundary of this range is given by the muon selection with $p_{\mathrm{T}}^{\mu } > 25\,{\text {Ge}\text {V}}$, as described in Sect. 5.1, assuming no other hard-scatter vertex tracks, i.e. $E_{\mathrm{T}}^{{\text {miss}},{\text {soft}}} = 0$. The upper boundary indicates the lower limit of $\Sigma E_{\mathrm{T}}$ to accommodate at least one jet with $p_{\mathrm{T}}^{\text {jet}} > 20\,{\text {Ge}\text {V}}$ in addition the two muons (for the jet selection see Sect. 3.3.5).

8 As discussed in Sect. 3.4, the soft term represents only charged particles with $p_{\mathrm{T}} > 400\,{\text {Me}\text {V}}$ not associated with fully identified and reconstructed particles or jets. Therefore, including $\mathbf p _{\mathrm{T}}^{{\text {soft}}}$ can only recover a part of the actual soft $p_{\mathrm{T}}$-flow of the interaction.
